# Tenuous transcriptional threshold of human sex determination. II. SRY exploits water-mediated clamp at the edge of ambiguity

**DOI:** 10.3389/fendo.2022.1029177

**Published:** 2022-12-02

**Authors:** Joseph D. Racca, Deepak Chatterjee, Yen-Shan Chen, Ratan K. Rai, Yanwu Yang, Millie M. Georgiadis, Elisha Haas, Michael A. Weiss

**Affiliations:** ^1^ Department of Biochemistry and Molecular Biology, Indiana University School of Medicine, Indianapolis, IN, United States; ^2^ Faculty of Life Sciences, Bar Ilan University, Ramat Gan, Israel

**Keywords:** DNA dynamics, nucleic-acid recognition, DNA intercalation, protein hydration, indirect readout

## Abstract

Y-encoded transcription factor SRY initiates male differentiation in therian mammals. This factor contains a high-mobility-group (HMG) box, which mediates sequence-specific DNA binding with sharp DNA bending. A companion article in this issue described sex-reversal mutations at box position 72 (residue 127 in human SRY), invariant as Tyr among mammalian orthologs. Although not contacting DNA, the aromatic ring seals the domain’s minor wing at a solvent-exposed junction with a basic tail. A seeming paradox was posed by the native-like biochemical properties of inherited Swyer variant Y72F: its near-native gene-regulatory activity is consistent with the father’s male development, but at odds with the daughter’s XY female somatic phenotype. Surprisingly, aromatic rings (Y72, F72 or W72) confer higher transcriptional activity than do basic or polar side chains generally observed at solvated DNA interfaces (Arg, Lys, His or Gln). Whereas biophysical studies (time-resolved fluorescence resonance energy transfer and heteronuclear NMR spectroscopy) uncovered only subtle perturbations, dissociation of the Y72F complex was markedly accelerated relative to wild-type. Studies of protein-DNA solvation by molecular-dynamics (MD) simulations of an homologous high-resolution crystal structure (SOX18) suggest that Y72 *para*-OH anchors a network of water molecules at the tail-DNA interface, perturbed in the variant in association with nonlocal conformational fluctuations. Loss of the Y72 anchor among SRY variants presumably “unclamps” its basic tail, leading to (a) rapid DNA dissociation despite native affinity and (b) attenuated transcriptional activity at the edge of sexual ambiguity. Conservation of Y72 suggests that this water-mediated clamp operates generally among SRY and metazoan SOX domains.

## Introduction

**Figure 1:**
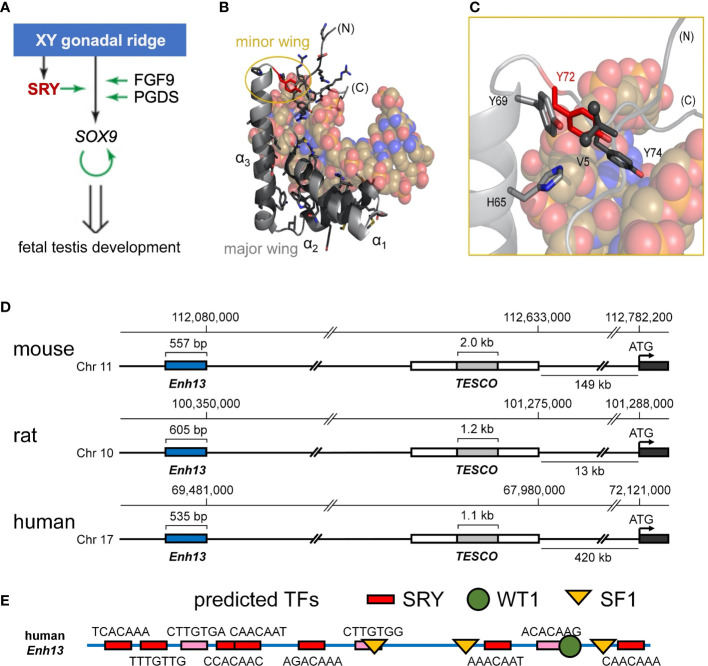
Biological function of Sry and structure of the HMG box. **(A)** Scheme of the initiation of male-sex differentiation in therian mammals. SRY (red) plays a key role in this process by activating expression of *SOX9*, which then autoactivates itself, leading to stage-specific expression of *FGF9* and *PGDS* as indicated by green arrows. **(B)** Ribbon representation of the HMG box of the human SRY HMG box-DNA complex (PDB coordinates 1J46), HMG domain in light gray ribbon and DNA shown as atomic spheres with carbon (C) tan, nitrogen (N) blue, oxygen (O) red, and phosphorus (P) orange). Residues for which clinically relevant variants have been identified are shown in black stick models (Y72 in red). The major wing is formed by the confluence of the three helices in the L-shaped domain (3). The minor wing is formed upon DNA binding (gold circle). **(C)** Expanded view of the minor wing and the DNA-dependent hydrophobic mini-core (4). Residues of the hydrophobic mini-core are shown as stick models (methyl groups of the N-terminal Val 5 are shown as spheres); Y72F is shown in red. **(D)** Chromosomal locations of *S9* upstream enhancer elements for rat, human, and mouse chromosomes. *TESCO* [testis enhancer sequence core (5)] is in gray, further upstream is Enhancer 13 (*Enh13* (6),) region in blue. **(E)** Identification of consensus transcription factor binding elements within the 535 bp of the human *Enh13* region. Binding site elements were identified using PROMO (7, 8).

Male development in the embryogenesis of therian mammals is initiated by stage-specific expression of a Y-encoded transcription factor (TF) in the bipotential gonadal ridge (1). Encoded by single-copy gene *SRY* (*s*ex-determining *r*egion of the *Y* chromosome) (2), this protein (designated SRY^1^) activates a male transcriptional program in a gonadal supporting-cell lineage (**Figure 1A**). Ensuing differentiation of fetal Sertoli cells (9) initiates testicular morphogenesis, a complex developmental program that includes differentiation of Leydig cells, formation of seminiferous cords and in-migration of germ cells within a non-specific mesenchyme, bounded by coelomic epithelium and developing vasculature (10). Fetal hormonal secretions—anti-Müllerian hormone/Müllerian inhibiting substance (AMH/MIS) and testosterone—respectively direct regression of female primordia (11) and external virilization (12) (**Supplemental Figure S1A**). Underlying molecular mechanisms have been informed by genotype-phenotype relationships observed among diverse clinical syndromes, collectively designated *differences* (or *disorders*) *of sexual differentiation* (DSD) (13). This and our companion study (14) pertain to Swyer syndrome: 46, XY gonadal dysgenesis due to mutations in SRY. Clinical features include female somatic phenotype with primary amenorrhea (15–17).

Human SRY is a protein of 204 residues containing a central DNA-bending motif (residues 56-141) (18). The latter, a member of the high-mobility-group (HMG) box superfamily (19), recognizes a short DNA target site [core element 5’-ATTGTT-3’ and complement (20, 21)]. The SRY HMG box defines a prototype for an extensive family of homologous domains, designated SOX (SRY-related HMG box; **Supplemental Figure S2A**) characteristic of a broadly conserved family of metazoan TFs (22). SRY domain-DNA complexes (3, 23) and SOX domain-DNA complexes (24–27) are remarkable for insertion of an L-shaped α-helical motif (4) within an expanded and underwound DNA minor groove, leading to sharp DNA bending (angles 60-80°) (3). A distinctive feature of SRY/SOX HMG boxes add closed parenthesis (relative to the LEF/TCF-1 family (**Supplemental Figure S2C**)) (28) and structure-specific HMG boxes (19, 29)) is its C-terminal basic tail, which contains a nuclear localization signal (NLS) (30, 31) and contributes to DNA recognition (32). Partial truncation of the tail preserves specific affinity (dissociation constant K_d_) with compensating acceleration of both association and dissociation (33). Such kinetic compensation in general reflects off-setting perturbations in rate constants (*i.e*., numerator and denominator in equation K_d_ = *k*_off_/*k*_on_). Cancellation is formally possible because association rates of SRY/SOX domains are slower than diffusion-controlled (34), presumably due to kinetic barriers imposed by structural reorganization of both macromolecular components (4).

Swyer mutations in SRY cluster within its HMG box and tail (**Figure 1B** and **Supplemental Figure S1B**) (35). Such mutations have been observed at the immediate protein-DNA interface (23), within the domain interior (21, 23) and within cellular signals for nucleocytoplasmic trafficking (NLS and nuclear export signal; NES) (30, 31, 36). The present study focuses on a mutational “hot spot” at the box-tail junction, an invariant Tyr at box position 72 (**Figure 1C**, residue 127 in intact human SRY) (15–17). The aromatic side chain occupies an “unremarkable” site at one edge of a hydrophobic “mini-core” [within the box’ minor wing (23)] and projecting into solvent near (but not at) the protein-DNA interface (3). Although potential aromatic-aromatic interactions (37) occur in the minor wing (Y69-Y72; **Figures 1B, C**), it is unusual among protein families for a Tyr to be conserved, *to the exclusion of other aromatic amino acids*, at such a structural environment (37, 38). Whereas among homologous SOX domains, for example, the conserved aromatic residue at box position 69 may be Tyr or His (39), the analogous Tyr→His substitution at box position 72 in SRY markedly impairs specific DNA binding [as monitored by gel mobility-shift assay (14)], which attenuates occupancies of SRY-regulated far- and near-upstream testis-specific enhancer elements of *Sox9*/*SOX9* (14) [**Figure 1D** (40)]. These enhancers, designated *Enh13* and *TESCO* (5, 6), contain several putative sites for sex determination-related transcription factors (**Figure 1E**; for *TESCO* region, see ref (5)). This enigma is deepened by the seeming compatibility of this site, in molecular modeling, with diverse aromatic and non-aromatic substitutions, including polar or basic side chains that (in unrelated systems) are often observed near protein-DNA interfaces (41). The anomalous conservation of Y72 (residue 127 in human SRY) among mammalian Sry orthologs (**Supplemental Figure S2B**; with the exception of W72 in SOX30) suggests a gap in understanding; might this side chain have a cryptic function not fully appreciated in past structural studies?

**Figure 2:**
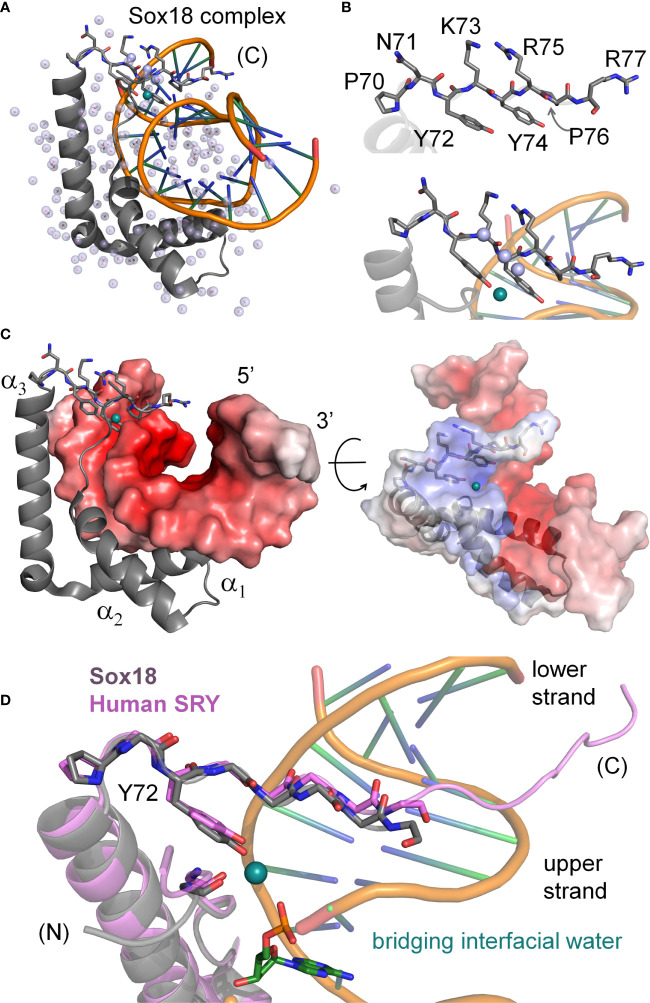
Water-mediated bridge in Sox18 crystal structure. **(A)** The high-resolution structure of SOX18 (gray) bound to DNA (orange spline, blue sticks) is shown as a ribbon rendering with water molecules as light blue spheres (PDB coordinates 4Y60). Residues of the C-terminal tail [(C)] are shown as a stick model. In teal is a bridging water molecule that is hydrogen-bonded to the protein and the DNA in the crystal structure; other waters are shown as pale blue spheres. **(B)** Top, expanded view of the C-terminal tail residues, labeled with consensus HMG numbering scheme. Bottom, view of the Tyr72 and associated teal water at the complex interface. Other waters in pale blue spheres hydrogen bond to the polypeptide main chain NH (amide) and C=O (carbonyl) positions. **(C)** Sox18-DNA complex with helices of the HMG domain labeled and the DNA represented in an electrostatic potential surface map. C-terminal tail residues are shown as sticks and bridging water in teal at the protein-DNA interface. Right, rotation of the complex by ~45° and both protein and DNA shown as semi-transparent electrostatic potential surfaces. HMG ribbon is shown with tail residues as sticks. The bridging water occupancy is at the interface of the protein-DNA complex in the crystal structure. **(D)** Alignment of the C-terminal region Sox18 and human SRY HMG box. The Sox18 domain is in gray and the human HMG domain in light purple as indicated. Consensus positions Pro70 and Tyr72 side chains are shown as sticks as well as the polypeptide chain of the tail. The water-bridge interactions include the *para-*hydroxyl of Tyr72, and oxygen atoms of the DNA phosphate backbone and a carbonyl position of residue 5 in the N-terminal β-strand.

The minor wing of the HMG box is largely disordered in the absence of DNA (**Supplemental Figure S3**) and rearranges upon specific DNA binding (4). Tethering of the N-terminal β-strand and the C-terminal tail is achieved by a single aliphatic side chain (box residue 5) that packs into a hydrophobic pocket created by conserved aromatic residues in the C-terminal end of the domain (23). This is in contrast to the major wing which remains well-ordered independent of DNA binding due to the presence of a major-wing hydrophobic core. The DNA-induced mini-core of the minor wing is composed of the conserved aromatic residues at consensus HMG box positions 65, 69, and 72 (**Supplemental Figure S2**). In the bent-DNA protein complex of human SRY and in homologous structures (3, 23, 25–27, 42–44), the mini-core creates a cavity at the protein-DNA interface. In the high-resolution murine Sox18 crystal structure^2^, interfacial water molecules within this cavity are present (**Figure 2**). The Sox18 crystal structure has 194 crystallographic waters associated with the complex (**Figure 2A** and **Supplemental Table S1**). The HMG tail-DNA interfacial cavity is flanked by a cluster of basic-rich amino acids (**Figure 2B**) and the DNA backbone. These surfaces are complementary in their overall electrostatic nature (**Figure 2C**); the bridging water molecule in the Sox18 complex is located at this interface. On alignment of the NMR structure of the human SRY box-DNA complex (3) with the co-crystal structure of the Sox18 box-DNA complex (25), homologous Tyr72 side chains exhibit similar positioning relative to the cavity, but the respective DNA backbone conformations differ (**Figure 2D** and **Supplemental Figure S4**)^3^. To assess a potential cryptic function for this conserved Tyr residue shared between the hydrophobic mini-core and the interfacial cavity of complex, we have further characterized biochemical functions of the WT and variant Sry domains and performed molecular dynamic (MD) simulations to differentiate between a water-mediated hydrogen bond network at this interface involving the Tyr or if the water observed in the Sox18 crystal structure is due to crystallography conditions or packing of the lattice. A systematic set of MD simulations in the Sox18 context permitted assessment of representative low-activity variants (Ala, Gln, and His) and functional SOX30-related substitution Y72W.

The above questions motivated the present studies. In our companion article (14) we demonstrated that *de novo* Swyer mutations at 127, box position 72 [arising in paternal spermatogenesis (15, 16)] confer marked, yet distinct molecular defects *in vitro* and in cell culture. Although specific DNA bending was generally retained, as probed by permutation gel electrophoresis (PGE) and steady-state fluorescence resonance energy transfer (FRET), Y127H perturbs box-DNA affinity and hence testis-specific enhanceosome occupancy, whereas Y127C destabilizes the intact TF to proteasomal degradation in cells (14). In each case, gene-regulatory activity was impaired in accordance with DSD phenotypes (36, 45). To recapitulate the male switch *in vivo*, our cell-based studies paid particular attention to SRY expression levels (via plasmid dilution or a Tet-*on* system (14, 36)). In these cellular models SRY-directed changes in downstream gene expression resemble results of stage-specific transcriptional profiling of the differentiating gonadal ridge in XY mouse embryos (46). Central to the molecular logic of this male-specific gene-regulatory network (GRN) is SRY-directed transcriptional activation of *SOX9* (**Figure 1A**) (47), an autosomal DSD-associated gene conserved among mammals and more broadly among vertebrates (48). Binding of Sry at enhancer regions upstream of *Sox9* (**Figure 1D**) allow for the enhanceosome formation and upregulation of *Sox9*, whose protein product in turn binds to its own sites in the regions to retain expression. Clinical mutations in SOX9 cause *campomelic dysplasia*, a complex syndrome of abnormalities of bone and cartilage (49) associated with 46, XY gonadal dysgenesis as a tissue-specific phenocopy of Swyer mutations in SRY (50). The present and companion studies (14) thus exploited SRY-directed activation of *SOX9* in appropriate SRY-responsive rat- and human cell lines as a quantitative probe of a male-specific embryonic GRN.

Functional effects of representative amino-acid substitutions at box position 72 (Ala, Cys, Gln, His, Lys, Phe and Trp) were first investigated in an embryonic pre-Sertoli XY cell line and in the isolated HMG box. Following this survey, we focused on inherited Swyer mutation Tyr→Phe (17). Unlike the predominant class of *de novo* mutations (51, 52), this subtle interchange of isosteric aromatic rings is shared by the proband’s father and hence is compatible with either female or male somatic phenotypes. Exemplifying *incomplete penetrance* as often encountered among monogenic endocrine syndromes (53–55), inherited Swyer mutations provide an experimental opportunity to define molecular mechanisms at the borderline of genetic function (36, 56, 57). The divergent developmental outcomes—fertile father and sterile XY daughter (17)—stand in striking contrast to the chemical similarity between Tyr and Phe. Indeed, only mild Y72F-related perturbations were uncovered in our preceding study: the variant HMG box exhibits near-native specific DNA-binding and bending properties in seeming accordance with an isosteric substitution in an “unremarkable” structural site (14). These findings rationalized the unperturbed phenotype of the father, but what of the sex-reversed daughter? To address this salient question, we have deepened our approach through biophysical studies and MD simulations. Biochemical assays were extended to higher-resolution probes of structure and dynamics [heteronuclear NMR spectroscopy, stopped-flow FRET and time-resolved FRET (33, 58, 59)]. Although the structures of the wild-type (WT) and variant box-DNA complexes are similar, these biophysical techniques enabled comparison of non-equilibrium DNA-binding properties: kinetic lifetime of the protein-DNA complex (56, 57, 60) and long-range conformational fluctuations in the sharply bent DNA (33, 56, 61). Further insight was obtained from analysis of homologous SOX-DNA co-crystal structures (25, 26), validated through construction of chimeric “box swap” *SOX18*-*SRY* genes containing a representative SOX HMG box or its Y72F variant. MD simulations of the variant Sox18 box-DNA complexes were undertaken to correlate gene-regulatory function with structure and solvation.

The present studies provide evidence for a novel water-mediated clamp in SRY, conserved among the family of metazoan SOX factors (62) and likely also among a broader metazoan superfamily of architectural TFs (19, 29). This clamp is anchored by the *para*-hydroxyl group of Y72, absent in the F72 variant. “Unclamping” the Y72F box-DNA complex leads to a marked acceleration in its dissociation and subtle perturbation of DNA bending (as suggested by time-resolved FRET), presumably associated with enhanced local conformational fluctuations at the tail-DNA interface [as suggested by Sox18-based MD simulations (63)]. Destabilization of this bound water molecule was also observed in MD simulations of low-activity variants (Tyr→Phe Ala, Gln and His), whereas analogous long-range clamping occurred in high-activity Sox30-related variant Tyr→Trp in accordance with the solution structure of the Lef-1 box-DNA complex (42). The key role of a single water molecule bound by a conserved Tyr side chain near the tail-DNA interface highlights solvation as a critical feature of the specific HMG box. Clinical recognition of Y127F as an inherited Swyer mutation (17) thus illuminates a water-dependent mechanism conserved among mammalian testis-determining factors; this mechanism is likely to extend broadly to metazoan SOX domains (64). Removal of a single atom from SRY (the *para*-oxygen of an invariant Tyr) thus positions a human genetic switch at the knife-edge of sexual ambiguity. Such variable phenotypes both highlight the biophysical importance of water-mediated interactions in protein-DNA recognition (41, 65–68) and honor in the breach the general Waddington principle of developmental canalization (69, 70).

## Materials and methods

### Protein expression and purification

The human SRY HMG box (box residues 2-86; fragment 57-141 of the intact protein) was subcloned in the pTXB1 expression vector (New England Biolabs) following the manufacturer’s protocol. Such cloning creates a chitin-binding intein-fusion protein, amenable to purification using chitin agarose. Protein product is collected after DTT-induced cleavage of the intein, separating the HMG-box domain from the rest of the fusion protein. A second purification step was provided by reverse-phase high-performance liquid chromatography (HPLC). The purified proteins were characterized by liquid-chromatography mass-spectrometry (LCMS). Mutations at box position 72 were made using QUIKCHANGE II XL site-directed mutagenesis kit (Agilent). Correct sequences were verified by Sanger sequencing (Genewiz). For ^15^N-labeled proteins, bacteria were grown in minimal media with ^15^N-ammonium chloride.

### Permutation gel electrophoresis

SRY-directed specific DNA bending was evaluated by permutation gel electrophoresis as described in our companion article in this issue (14). Four technical replicates were in each case obtained. To avoid confounding effects of lane positioning in the gels, each complex was run either on the left side of the gel, on the right side of the gel, or in random order with respect to flexure displacement. Temperature was controlled at 4 °C by a circulating water bath.

### FRET-based DNA-binding and kinetic assays

Steady-state FRET was employed to determine protein-DNA dissociation constants (K_d_) and relative DNA bending of the WT and variant domains. Measurements were made in FRET buffer (10 mM potassium phosphate, 10 mM Tris-HCl, 140 mM KCl, and pH 8.0) at 15 °C and 37 °C using the DNA sequence 5’-TCGGTGATTGTTCAG -3’ (target sequence underlined). For this experiment, the 5’ end of the top strand (15 base pairs; bp) was labeled with fluorescein serving as the donor and the 5’ end of the bottom strand with TAMRA as the acceptor fluorophore; an aliphatic hexanyl linker was used to attach the fluorophores on each strand. Purified labeled DNA oligonucleotides were obtained from GenScript. For relative DNA bending studies, the samples included equimolar concentrations (1 μM) of fluorescently labeled DNA and HMG domains in FRET buffer. For K_d_ determinations, the concentration of wild-type or variant SRY domain was varied while the DNA concentration was held constant at 25 nM. Emission spectra were recorded from 500-650 nm following excitation at 495 nm on a Jasco FP 8300 spectrofluorimeter. Estimates of K_d_ were determined by plotting change in fluorescence intensity at 525 nm against total protein concentration. Data were fit with a single-site ligand-binding model (Equation 1) as described (71) using Origin 8.0 software (OriginLab Corp., Northampton, MA).


(1) $$\Delta F=\Delta F_{o}\left\{0.5(1+S/D_{o}+K_{d}/D_{o})-{\left[0.25{\left(1+S/D_{o}+K_{d}/D_{o}\right)}^{2}-S/D_{o}\right]}^{0.5}\right\}$$

In this formalism ΔF is the change in donor fluorescence observed on addition of the SRY domain relative to the baseline DNA fluorescence; ΔF_0_ is the maximum fluorescence change obtained in a 1:1 protein-DNA complex; K_d_ is the dissociation constant; D_0_ is the concentration of DNA (25 nM); and S is the concentration of SRY domain. Stopped flow FRET (sf-FRET) was used to measure *off*-rate constants (*k*_off_) of WT and variant SRY domains. A dual-syringed stopped flow device was loaded with the specific FRET-labeled DNA-domain complex (1 μM), and the second syringe was loaded with excess unmodified DNA (20 μM). These samples are rapidly mixed in the cell of the fluorimeter; excitation and emission wavelengths same as above are used.

### Circular dichroism spectroscopy

The above 15-bp DNA duplex without fluorescent probes was complexed with WT or variant SRY domains (25 μM each component) in a buffer (10 mM potassium phosphate and 140 mM KCl at pH 7.6). Spectra were acquired using a JASCO J-1500 spectropolarimeter in the near-ultraviolet range 320-250 nanometers at 4 and 15 °C; spectra features in this range are sensitive to DNA structure (72) whereas relative contributions from protein are negligible.

### Structural analysis of homologous structures

The presence of ordered water molecules in crystal structures is highly dependent on the resolution of the structure in the sense that the resolution reflects the level of overall order for the macromolecular components. Atomic *B*-factors in the coordinate files for crystal structures are a relative measure of motion associated with each atom. The absolute B-factor values are generally higher for lower resolution structures, and in protein-DNA complexes, the DNA tends to have somewhat higher temperature factors than the protein component due to its inherent flexibility (**Supplemental Table S1**). Crystal structures are available for DNA-bound Sox18 (25), Sox11 (73), Sox4 (27), Sox9 (74), Sox17 (24), and Sox2 (26). For Sox 18, 11, and 9 the B-factors for the DNA are somewhat higher than for the protein. In both the Sox2 structure, which also includes POU domains bound to DNA, and the Sox4 structure, the B-factors for the HMG box are similar to those of the DNA. The Sox17 structure has unrealistically low B-factors for its resolution. Among these co-crystal structures there is a correlation between lower B-factors and ordered water molecules.

### NMR spectroscopy

2D ^1^H/^15^N heteronuclear single-quantum coherence (HSQC) spectra were acquired at 25°C using a 600 MHz Bruker AVANCE spectrometer equipped with a 5-mm triple-resonance cryoprobe (^1^H, ^13^C, and ^15^ N) and z-axis pulsed gradient. NMR experiments were processed using TOPSPIN. Proteins were dissolved in buffer containing 10 mM potassium phosphate and 50 mM KCl (10% D_2_O, pH 7.4) placed in a 280-μl Shigemi NMR tube.

### Mammalian cell-based assays

Plasmids expressing HA-tagged SRY variants were constructed by QuikChange™ II XL Multi Site-Directed Mutagenesis Kit (Stratagene) from template WT human SRY gene (36). The cloning site encoded an epitope tag in triplicate (derived from hemagglutinin [HA]) to enable Western blotting (WB) and chromatin immunoprecipitation (ChIP) assays. Constructions were verified by DNA sequencing. Synthesized plasmids expressing engineered chimera SRY containing human SOX18 HMG box and its homologous Tyr→Phe mutation were purchased from GenScript. The native SRY HMG box was replaced by the human SOX18 domain in these chimeric variants whereas native N-terminal and C-terminal SRY domains were retained. Expression of HA-tagged SRY constructs and SRY-SOX chimeras was regulated by a viral promoter (derived from cytomegalic virus [CMV]). Constructions were verified by DNA sequencing.

### Cell culture and transient transfections

Rodent CH34 cells (kindly provided by Dr. P.K. Donahoe, Massachusetts General Hospital) (36, 75) and human LNCaP cells (ATCC^®^ CRL-7002™ and ATCC^®^ CRL-1740™, respectively) were cultured in regular Dulbecco’s Modified Eagle Medium (DMEM) containing 5% fetal bovine serum (FBS) at 37 °C under 5% CO_2_ and in Dulbecco’s Modified Eagle Medium (DMEM) medium containing 10% FBS in 5% CO_2_ atmosphere. Transfections were performed using Lipofectamine 3000 as described by the vendor (Invitrogen) for 8 hours (h) in an improved Minimal Essential Medium (Opti-MEM; ThermoFisher).

### Chromatin immunoprecipitation and transcriptional activation assay

Cells transfected with plasmids encoding epitope-tagged WT or variant SRY were fixed in wells by formaldehyde, collected and lysed after quenching the cross-linking reaction. Lysates were sonicated to generate 300-400-bp fragments and immunoprecipitated as described (57). Specific pairs of forward (F) and reverse (R) DNA oligonucleotide primers were employed to probe embryonic testis-specific enhancer elements (*TESCO* (5) and *Enh13* (6, 40, 76); **Figures 1D, E**). Details are described in **Supplemental Information** of the companion study (14). In transient transfections, the SRY expression plasmid was diluted 1:50 with the empty parent plasmid to reduce protein expression to the physiological range (*ca*. 10^3-4^ protein molecules/cell (36)). After the transient transfection, cellular RNA was extracted and converted to cDNA using the vendor’s protocol (BioRad). SRY-mediated transcriptional activation was probed by SOX9/Sox9 mRNA as functional readouts; primer sequences were as described (57, 77). An internal control was provided by the specific 5′‐TATAA DNA‐binding subunit of basal transcription factor TFIID. Data analysis included three technical replicates of each of three biological replicates. Details were provided as **Supplemental Information** in our companion study (14).

### Molecular dynamic studies

A systematic set of MD studies was performed based on the Sox18 box-DNA co-crystal structure (**Supplemental Table S2**). All binding pathways simulations were initiated with pre-equilibrated states of the relevant WT or variant in complex with DNA using deposited mouse Sox18 box-DNA complex^3^ (PDB 4Y60) using the AMBER99SB protein nucleic AMBER94 (78) force field in GROMACS 2019 (79, 80). The related protein-DNA complex was placed in the center of a water box equidistant from all edges and ions added to create a zero net charge. The system should not have any steric clashes or inappropriate geometry. Thus, the structure is relaxed through a process of energy minimization. Each complex was energy minimized, using the steepest descent algorithm, until the Fmax was found to be smaller than 10 kJ.mol^−1^.nm^−1^. All of the covalent bonds were constrained using the Linear Constraint Solver (LINCS) algorithm to maintain constant bond lengths. The long-range electrostatic interactions were treated using the Particle Mesh Ewald (PME) method, and the cut off radii for Coulomb and Van der Waals short-range interactions was set to 1.0 nm. The modified Berendsen (V-rescale) thermostat and Parrinello–Rahman barostat, respectively, were applied to keep the system in stable environmental conditions (310 K, 1 Bar). Finally, uniform molecular dynamic simulations were carried out under the periodic boundary conditions (PBC), set at XYZ coordinates to ensure that the atoms remain inside the simulation box. A starting structure for simulations of the Y72W, Y72A, Y72Q and Y72H complexes was generated from the 80-ns coordinates of the WT Sox18-DNA MD simulation—with rigid-body replacement of the side chain followed by local energy minimization—either retaining the WT solvent coordinates observed at that time point or creating a new box of 50,000 water molecules. Subsequent analyses were performed using GROMACS utilities in PyMol, VMD and USCF Chimera.

## Results

**Figure 3:**
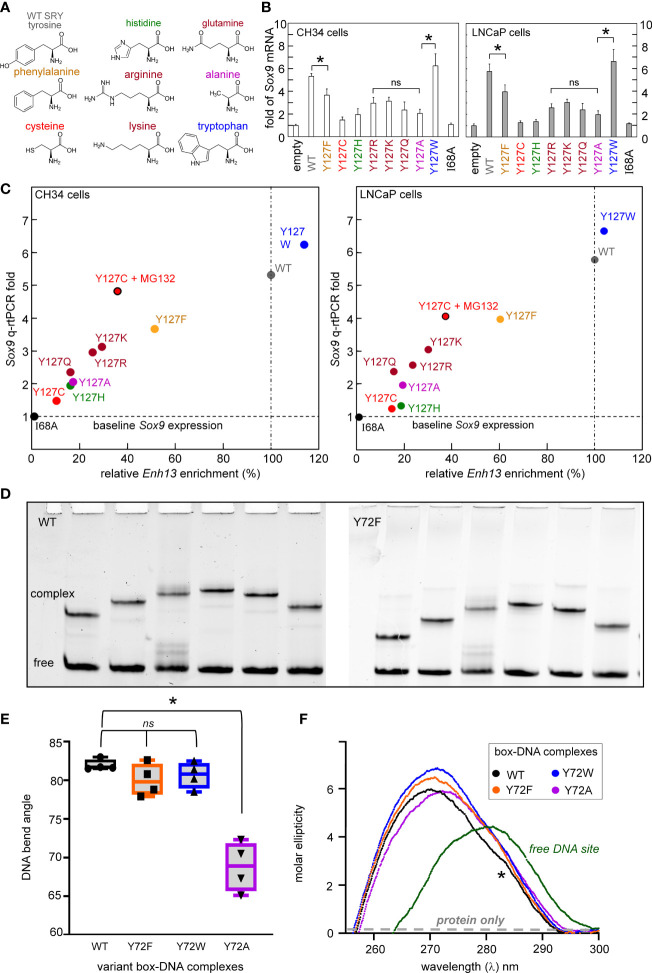
Survey of mutations at box position 72 of human SRY. **(A)** Structures of substituted amino acids. **(B)** Gene-regulatory activities activities of SRY variants tested in rodent pre-Sertoli XY cell line CH34 (left (75);) and human neoplastic XY cell line LNCaP (right (81, 82)): fold-change in *Sox9/SOX9* gene expression provides functional readouts. Statistical analyses: “ns” indicates no significant difference, asterisks indicate pairwise p values< 0.05. **(C)** Relationship between relative SRY enrichment on a testis-specific enhancer element (Enh13) and activation of *Sox9/SOX9* in CH34 (right) and LNCaP (right) cells. **(D)** Representative PGE studies of WT and Y72F SRY domain-DNA complexes. Each lane contains the protein-DNA complex and free DNA. Differences in mobility reflect placement of the 5’-A**TT**GTT-3’ target site within the 150-bp DNA fragment: respective distances in top DNA strand from its 5’-end to the bolded “TT” step are (a) 120 bp, (b) 95 bp, (c) 79 bp, (d) 51 bp, (e) 47 bp and (f) 27 bp. **(E)** Box-dot plot showing inferred DNA bend angles. Results of four technical replicates are shown in each case (for individual analyses of flexure-dependent electrophoretic mobilities, see **Supplement Figure S6**). In each case the solid horizontal line represents the mean bend angle, whereas the standard deviation is shown above and below the boxes. **(F)** Near-UV CD spectra of WT and variant domain-DNA complexes at DNA-sensitive wavelengths 250-320 nm. Spectra of complexes are shown in black (WT), orange (Y72F), blue (Y72W), and purple (Y72A) as indicated in inset; the spectrum of the free DNA is shown in green. WT complex exhibited a slight shoulder in the spectra (asterisk) not observed in variant domains. Contribution of the free protein within this range is minimal (schematic gray dashed line indicates “protein only”; data not shown). Protein-bound spectra each exhibit A-like features in accordance with widening of the DNA minor groove and overall DNA under-winding (see **Supplemental Figure S6**).

Our approach of study integrated biochemical and biophysical assays with MD simulations. We first undertook a functional survey of amino-acid substitutions at box position 72 (**Figure 3A**) as evaluated in gene-regulatory studies of intact human SRY (**Figure 3B**). To this end, SRY-directed transcriptional activation of principal target gene *Sox9/SOX9* was probed by quantitative real-time reverse-transcriptase PCR (qPCR) in rodent XY cell line CH34 and human XY cell line LnCaP (left and right boxes in **Figure 3B**; see companion article and its SI (14)): trends in activity, shown by histogram, are similar in the two cell lines. In each case Y72F reduced activity by *ca.* 30% whereas Y72W enhanced activity by 10-15%; the remaining substitutions reduced activity by *ca.* tenfold (clinical mutations Cys and His (14)) or 15-40% (Ala, Arg, Gln, and Lys). Similar trends were observed in ChIP studies of testis-specific enhancer occupancies upstream of the *Sox9/SOX9* transcriptional start site (**Figure 3C**; enhancer element locations as outlined in **Figure 1D**). In the case of Y72C reduced activity largely reflects accelerated proteosomal degradation in these cell lines (14).

We extended this survey *in vitro* through biochemical analysis of SRY-directed DNA bending by permutation gel electrophoresis (PGE). These studies employed the isolated SRY HMG box and were repeated four times (**Figure 3D**). In accordance with related studies in our companion study (14), the relationship between flexure displacement and electrophoretic mobility (83) in each case implied sharp DNA bending (**Supplemental Figure S6A**). Whereas DNA bend angles were indistinguishable among the WT, Y72F and Y72W domain-DNA complexes (black, orange and blue boxes in **Figure 3E**), Y72A led to a marked reduction in inferred bend angle (purple; from to *ca*. 80 to 70°; **Supplemental Table S3**). Despite similarities in PGE-defined DNA bend angles, near-UV CD studies of free- and bound 15-bp DNA sites (wavelengths 250-300 nm) provided evidence for biophysical differences among bound DNA structures (**Figure 3F**). Contributions to ellipticity by the protein are negligible in this spectral region (highlighted in schematic form by gray dashed line in **Figure 3F**). Whereas as expected, the free DNA exhibits a B-DNA pattern, in each case the bound DNA spectra exhibit A-form features (**Supplemental Figure S6B**) (72). Peak band positions are given in **Supplemental Table S3**. The CD spectrum of the Y72A complex (purple in **Figure 3F**) is least shifted toward a canonical A-DNA profile in accordance with its attenuated DNA bend angle. The spectrum of the WT SRY box-DNA complex (black in **Figure 3F**) exhibits a distinctive shoulder near 283 nm (asterisk). Physical interpretation of relative band heights (Y72W>Y72F>WT) is not well understood.

**Figure 4:**
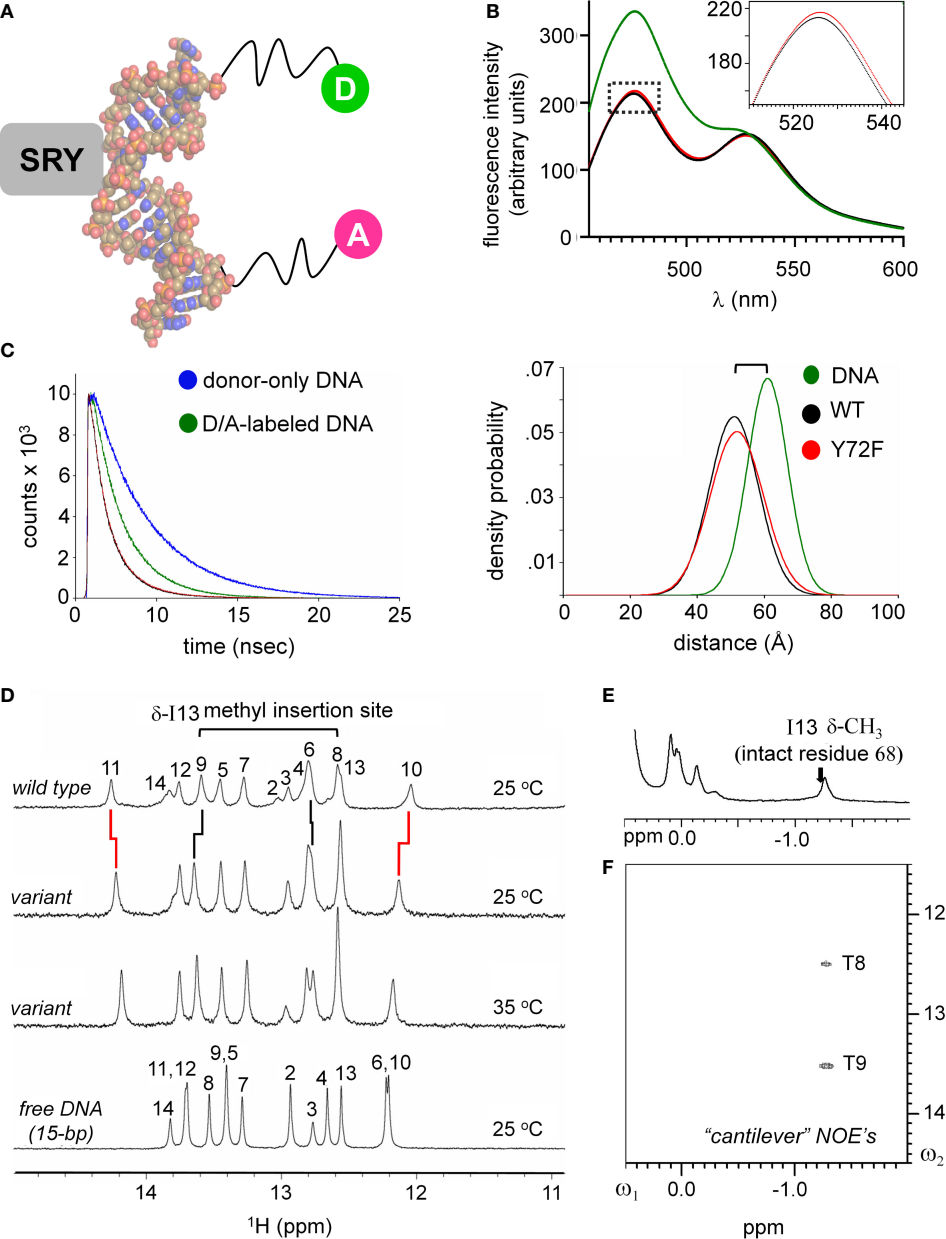
FRET and NMR studies of human SRY HMG-DNA complex. **(A)** Sphere representation of an SRY-directed (gray box) bent DNA structure (25). The FRET fluorophore set used in these experiments are labeled as: donor; light green sphere “D” and acceptor; magenta sphere “A” each are attached to one 5’-end of the complementary DNA strands with a hexynyl-linker (61). **(B)** Steady state FRET spectra of the double-labeled DNA (green), WT complex (black) and the Y72F variant domain (red). Dotted box region is expanded in inset and highlights a subtle difference in the peak maxima of the donor fluorophore for the variant complex. **(C)** Fluorescence decay of the donor bound to the 5’-end of the DNA using an excitation of 490 nm and detected at an emission wavelength of 520 nm. Donor-only trace in blue and the donor/acceptor-double labeled DNA (D/A-DNA) is in green. Fluorescence decay for the WT (black) and Y72F (red) mutant are similar. (Right) Skewed Gaussian models of end-to-end distance distributions comparing free DNA and the two complexes. Binding of the HMG box reduces the end-to-end distribution (black and red). The slight increased width of the mutant complex (red) reflects, primarily, long-range conformational fluctuations. **(D)** ^1^H NMR protein-DNA interactions, assignments are as indicated (numbering scheme; top); spectra of free DNA (bottom), native SRY-DNA complex (top) at 25 °C and variant-DNA complex at 25 and 35 °C (middle). Vertical segments between variant wild-type indicate small differences in chemical shifts. The resonances of base pairs 2 and 14 are broadened due to fraying. Horizontal bracket at top site of side-chain insertion between bp 8 and 9 by “cantilever” residue Ile13. **(E)** Two-dimensional 1H NMR NOEs diagnostic of SRY-DNA intercalation. ^1^H NMR **(E)** and NOESY spectra **(F)** of variant protein-DNA complex showing corresponding intermolecular NOEs involving I13-DNA. The mixing time was in each case 150 ms. Spectra were obtained at protein-DNA stoichiometries 1:1, 25 °C in 10 mM potassium phosphate (pH 7.4) and 50 mM KCl in 90% H_2_O and 10% D_2_O.

Protein-directed bending of a 15-bp DNA duplex (containing central target site 5’-ATTGTT-3’ [upper strand] and complement 5’-AACAAT-3’ [lower strand]) was further monitored by FRET and ^1^H- and ^15^N-NMR spectroscopy (33, 56, 61). These complementary assays respectively provided global (long-range) and local (short-range) biophysical probes. For FRET studies, by 5’-fluorescein (donor; upper strand) and 5’-TAMRA (acceptor; lower strand) were, flexibly attached *via* hexanyl linkers (**Figure 4A**). Given the repeat length of the double helix, choice of a 15-bp DNA site positions the fluorescent donor and acceptor on the same side of the free DNA cylinder; photophysical control studies verified their mobility (61). Specific binding of the SRY HMG box leads to a marked reduction in end-to-end distance, thereby enhancing the FRET signal (**Figure 4B**). This effect (observed as an attenuation of fluorescein emission and enhancement of TAMRA emission) was similar but not identical (inset box in **Figure 4B**) on binding of the WT domain (black spectrum) relative to the Y72F domain (red spectrum). This model enabled measurement of thermodynamic affinities (see our companion study (14)), and fluorescence lifetimes relative to control samples containing only the donor (**Figure 4C**, left) (61). Stopped-flow FRET also enabled measurement of protein-DNA dissociation rates (*k*_off_) (57, 60). The same DNA duplex, without 5’-modification, was used in 1D- and 2D-NMR studies. Local probes were provided by chemical shifts and inter-proton NOEs. MD simulations exploited a related co-crystal structure (Sox18 box-DNA complex (84); **Figure 2**), chosen based on its high resolution, abundance of crystallographic water molecules, homologous protein- and DNA sequences, and amenability to biological validation through analysis of chimeric human SOX18-SRY constructs.

Together, this interdisciplinary strategy sought to illuminate how and why removal of a single atom from SRY (the *para*-oxygen of a conserved Tyr) may initiate a subtle cascade of biophysical perturbations, leading to attenuated biological activity (14) and ultimately to ambiguity in a developmental fate decision (17). This motivation was deepened by the broad conservation of this residue in a metazoan superfamily of architectural transcription factors and hence by the potential generality of our findings (**Supplemental Figure S5**) (85–88).

### Subtle Y72F-associated perturbations in structure and dynamics

An independent probe of DNA bending (conjoined with DNA unwinding) is provided by time resolved-FRET measurement of fluorescent lifetimes. These data enabled gaussian modeling of distances between donor and acceptor in an ensemble (**Supplemental Table S4**)(for review, see (58)). Application to the free 15-bp DNA site, WT SRY box-DNA complex and variant complex yields end-to-end distance distributions (EEDD) as shown in **Figure 4C** (right). The bracket in **Figure 4C** (top right) highlights a shift in peak maxima between the free DNA (61 Å as expected for linker-extended 15-bp B-DNA; green) and the protein-DNA complexes (*ca*. 51 Å; black [WT] and red [Y72F]). This reduction in mean end-to-end distances is in qualitative accordance with the results of PGE. The widths of the respective EEDDs are due to the combined effects of fluorophore-linker flexibility, fraying of the terminal base pairs, and possible variation in DNA trajectory. Because free B-DNA is stiff on the scale of 15 bp (persistence length 45-80 nm [>130 bp] (89)), the baseline width of the free EEDD would be dominated by probe flexibility and terminal bp fraying (61). A slight reduction in energy-transfer efficiency in the Y72F complex relative to WT (from 71.0 to 67.5%) was observed in association with a small shift in inferred peak of the EEDD (from to Å; 51.0 to 51.8 Å; **Supplemental Table S4**). In addition, the protein-DNA complexes exhibited 20-25% broader distributions (as indicated also by reduced peak heights in **Figure 4C**, right; **Supplemental Table S4**), presumably due to variations in extent of DNA bending and unwinding in the ensembles. The breadth of the variant EDDD is slightly wider than that of the WT EDDD, suggesting a subtle increase in nonlocal conformational fluctuations. Such fluctuations would enable longer end-to-end DNA distances to be populated (*i.e*., less bent), thereby rationalizing the slight attenuation of steady-state FRET efficiency observed in the variant complex relative to WT (**Figure 4B** and **Supplemental Table S4**). Although the reduction in energy-transfer efficiency was more statistically robust than the inferred broadening of the distribution, the same trends were observed in all six pairs of experiments (three technical replicates using two independent pairs of samples). Such subtle differences in time-resolved FRET properties, restricted to 5’ end-to-end distance relationships, are consistent with the more marked differences observed in near-UV CD spectra (**Figure 3F** above), which represent an average measure of double-helical geometry (72).

^1^H-NMR spectra of the Watson-Crick imino resonances (hydrogen-bonded NH groups in thymidine [T] and guanine [G] bases) provide local base-pair-specific probes of DNA structure and dynamics (41). Imino chemical shifts in the free DNA and two protein complexes are given in **Supplemental Table S5**. The imino ^1^H-NMR spectrum of the free 15-bp DNA duplex (at bottom in **Figure 4D**) differs markedly from the imino spectrum of the WT SRY domain-DNA complex (at top in **Figure 4D**). Such marked changes in chemical shifts reflect protein-directed reorganization of the bound DNA structure. Protein titration of the WT or Y72F domain is in slow exchange on the time scale of NMR chemical shifts in accordance with the complex lifetimes defined by stopped-flow FRET (below). The imino ^1^H-NMR spectrum of the variant complex is similar but not identical to that of the WT complex (horizontal lines in **Figure 4D**). The largest complexation shifts in each case occur at the site of cantilever insertion (bracket at top in **Figure 4D**). Cantilever insertion is associated with a large upfield shift in the δ-methyl resonance of I13 (residue 68 in intact human SRY) due to the ring currents of the flanking AT base pairs in the interior of the bent DNA site (41). This upfield complexation shift is almost identical in the WT spectrum (arrow in **Figure 4E**) and in the variant spectrum (**Figure 4E**). In each case intermolecular NOEs were observed from the intercalated methyl group to the flanking two thymidine imino protons (**Figure 4F**). Attenuation of ^1^H-NMR imino complexation shifts was observed at base pairs 10 and 11 (**Supplemental Table S5**), far upfield and far downfield in the imino spectra region (broken vertical lines in **Figure 4D**). Similar attenuation was previously reported in comparative ^1^H-NMR studies of *de novo* Swyer variant M9I (residue 64 in intact human SRY) (37), inherited Swyer variant V5L (35) and in a C-terminal truncated domain (28) (**Supplemental Figures S7, S8**). As here, such attenuation was accompanied by EEDD broadening. We thus envision that long-range *global* variation in DNA bend angles due to perturbations of the minor wing or tail of the HMG box—as probed by time-resolved FRET—is generally associated by enhanced *local* conformational averaging of the G10 and T11 imino ^1^H-NMR resonance in the variant complexes.

**Figure 5:**
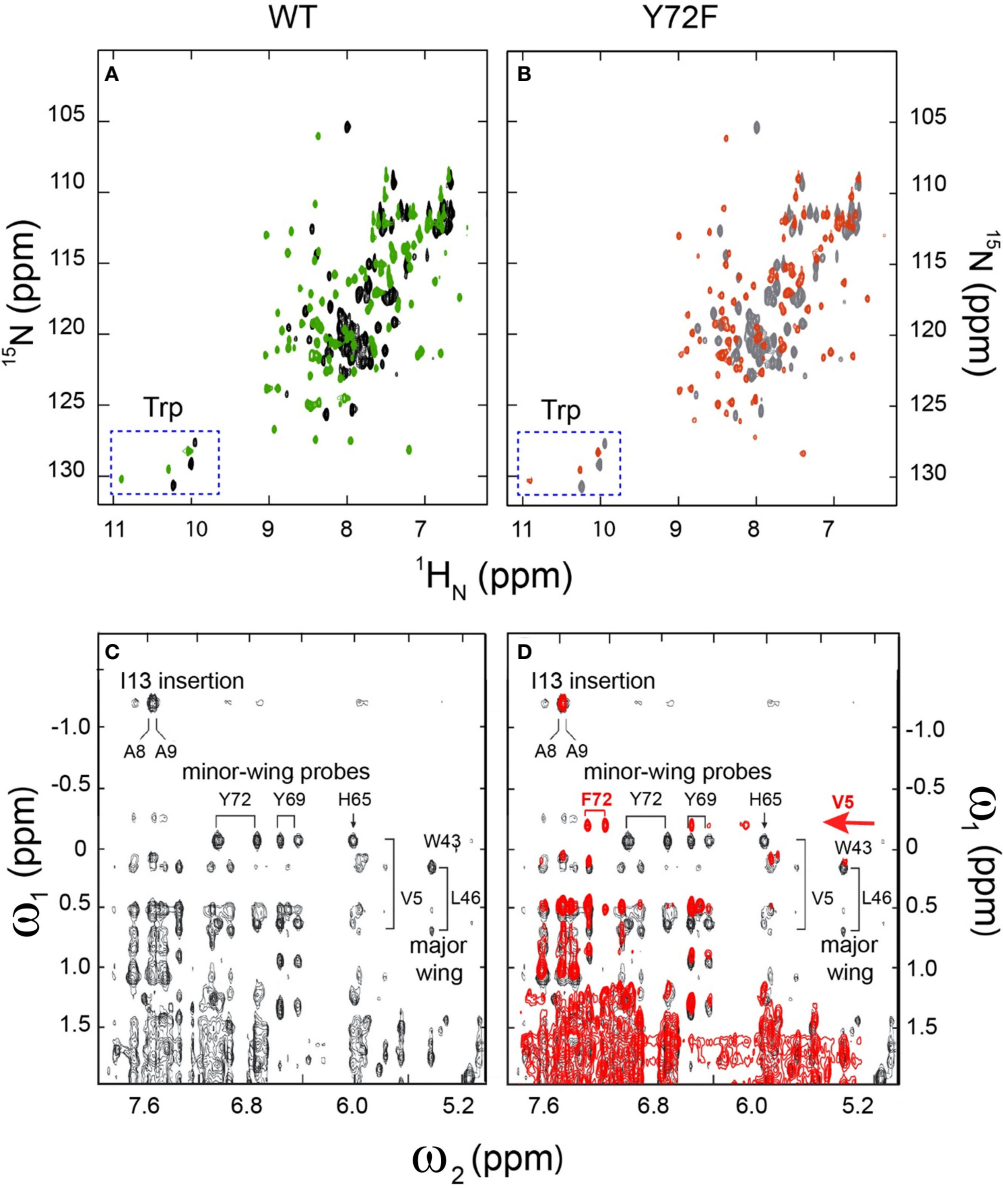
2D ^1^H-^15^N NMR HSQC footprints of free and protein–DNA complexes. **(A)** 2D HSQC spectral overlay of free WT (black), bound WT (green). **(B)** 2D HSQC spectral overlay of free Y72F(gray), bound Y72F(red). Both WT and Y72F undergo significant conformational change after binding. **(C)** NMR features of the DNA-stabilized minor wing, complexation shifts, and NOE contacts reflect the packing of the 1-methyl group of Val-5 within the aromatic rings of His-65, Tyr-69, and Tyr-72. **(D)** 2D NOESY spectral overlay of bound WT (black), bound Y72F(red). Y72F clearly shows the differences compare to WT. The new peak F72 appears in Y72F spectra and is marked in red. All spectra were acquired at 25°C.

Whereas the above probes pertain to DNA, an overview of protein structure was provided by ^1^H-^15^N HSQC spectra; these studies used domains uniformly enriched in ^15^N through biosynthetic expression (see Methods). Whereas the free WT or variant domains exhibit limited ^1^H and ^15^N chemical-shift dispersion [as previously described and attributed to flexibility of the free domains (20, 25)], in each case specific DNA binding leads to a marked enhancement of dispersion in both dimensions (**Figures 5A, B**). The spectra of the two free domains are essentially identical (**Supplemental Figures S9A-C**). The spectra of the two domain-DNA complexes are similar, with subtle changes in chemical shifts (**Supplemental Figures S9D-F**). As expected, the three indole NH resonances of Trp (W15, W43 and W52) exhibit essentially identical chemical shifts and complexation shifts in the WT and variant box-DNA complexes (**Supplemental Figure S10** and **Supplemental Table S6**), providing evidence that the major wing is not significantly perturbed by minor-wing mutation Y72F.

### Marked Y72F-associated perturbations in kinetic stability

**Figure 6:**
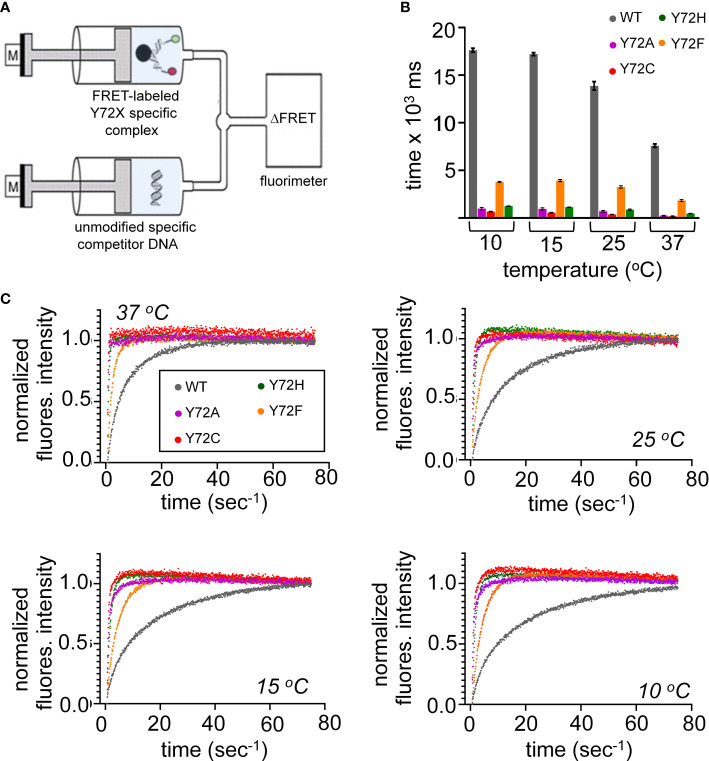
Kinetic measurements of WT and variant HMG domains. **(A)** Schematic of the stopped flow FRET design. One syringe contains the labeled-DNA in complex with WT SRY HMG box or variant, a second syringe contains 20-fold excess of unmodified DNA. Rapid injection of each syringe into a cell allows for kinetic measurements. **(B)** Lifetimes of the bent-DNA-protein complexes. Lifetimes are derived from 1/*k_off_* (see **Supplemental Table S1**) *ca* from measurement at 10, 15, 25, 37 °C (x-axis). **(C)** Representative traces are shown for stopped flow FRET of WT and the variant domains collected at four temperatures indicated in each plot. Values in **Supplemental Table S1**.

Kinetic analysis of bent DNA-protein complex is directly measured using stopped flow FRET; a labeled complex is rapidly mixed with an excess of unmodified DNA **(** **Figure 6A** **)**. Measurement of lifetimes of the Y72 variant complexes reveals that all variant domains have a decreased lifetime, *i.e*., an increase in the off-rate (*k*_off_) at four temperatures (10, 15, 25, 37 °C; histogram in **Figure 6B**). Kinetic data are shown in **Figure 6C** and summarized in **Supplemental Tables S7, S8**. At physiological temperature domains bearing *de novo* mutations (Y72C and Y72H; respective red and green bars in **Figure 6B** and corresponding data points in **Figure 6C**) exhibited such accelerated dissociation relative to WT that *k*_off_ values could not be determined at the time scale of stopped-flow; only an upper limit could be estimated (**Supplemental Table S7**). At 10 °C Y72C and Y72H mutations are respectively associated with 26(± 1.3)- and 14(± 0.38)-fold faster dissociation (**Figure 6B** and lower right panel of **Figure 6C**; **Supplemental Table S7**). That these clinical variants also retained near-native protein-DNA affinities {**Supplemental Figure S11** and **Supplemental Table S6** (14)] indicates marked kinetic compensation between *on*- and *off*-rates. Similar features were observed in control studies of substitution Y72A, not to date identified in a Swyer patient (purple in **Figures 6B, C** **)**. Tryptophan in this position displayed a slight increase in *k*_off_, but to a much lesser extent than all other variants with retained high-affinity DNA binding (**Supplemental Figure S11** and **Supplemental Table S7**).

Inherited Swyer mutation Y72F was observed to hasten protein-DNA dissociation at each temperature tested, but to a lesser extent than the *de novo* mutations (orange bars in **Figure 6B**; **Supplemental Tables S7, S8**). At 37 °C, for example, the *k*_off_ value of the Y72F HMG box was increased by fourfold relative to WT, compensated by a similar increase in *k*_on_. Among this set of clinical mutations, transcriptional potency, least perturbed by box substitution Y27F (clinical mutation Y127F in intact human SRY (14)), thus tracks with the kinetic lifetime of the domain-DNA complex and not its specific DNA affinity (K_d_), as hypothesized as a general mechanistic feature of architectural gene regulation (90) (see Discussion). In particular, the general trend toward kinetic compensation among the present variants, mitigating changes in K_d_, suggests that native docking of the basic tail within an expanded DNA minor groove (3) imposes kinetic barriers to both specific DNA binding and release. Partial truncation of the tail was previously shown to attenuate these barriers (**Supplemental Figure S8**) (33).

### Chimeric “Box Swap” SOX18-SRY constructs

**Figure 7:**
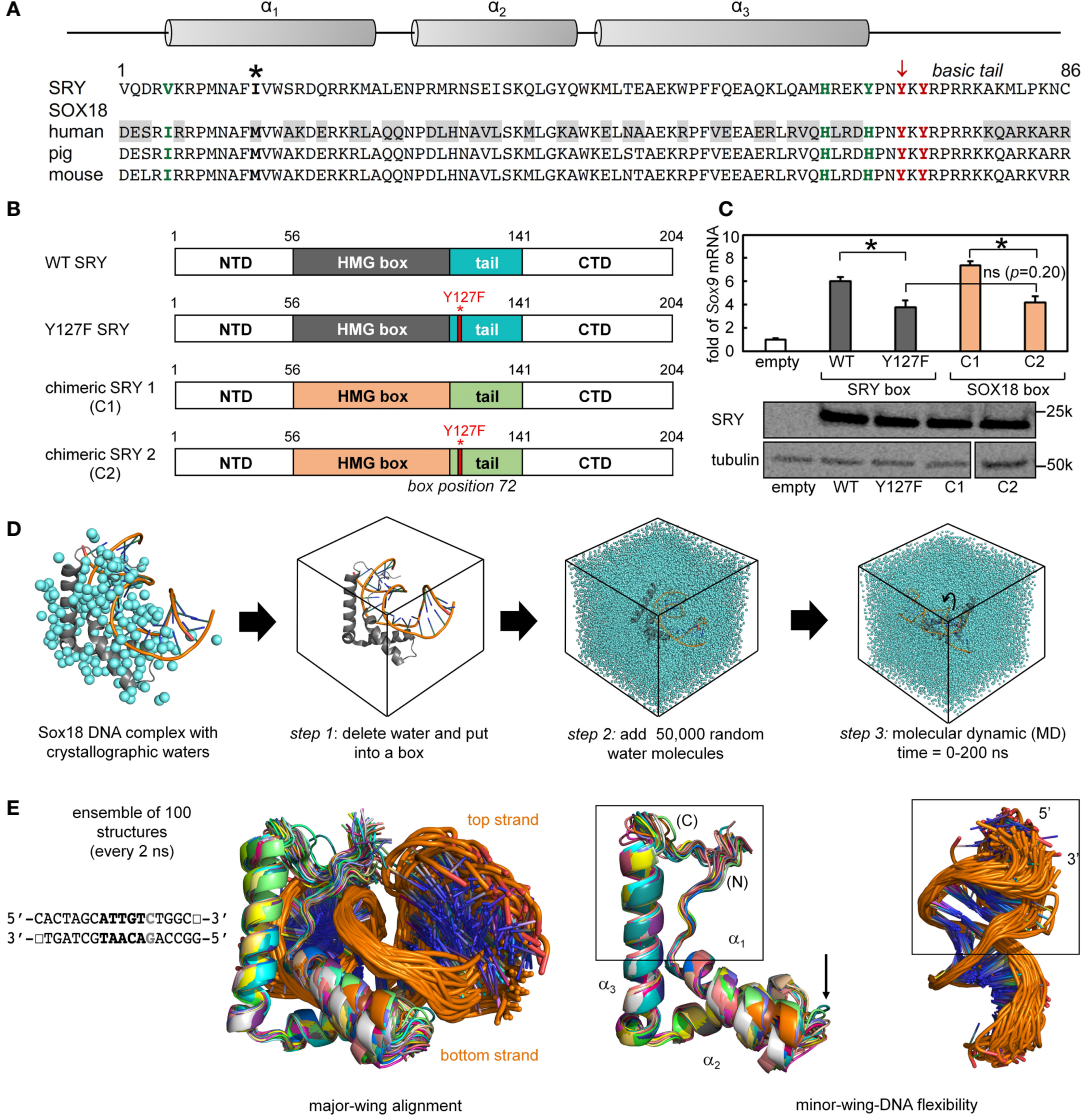
Mutation in position 72 affects the gene regulation function in SOX HMG box. **(A)** Schematic showing the three α-helix motifs in the HMG box domain (upper). Asterisk in sequence alignment indicates the “cantilever” intercalative residue. Bold residues in green and red highlight the important residues in forming the minor wing mini core in the associated sequences of human SRY and SOX18. Tyrosine in position 72 is indicated with an arrow (consensus HMG box number). Gray boxed residues in human SOX18 sequences indicate the residues with significant property changes compared to human SRY. **(B)** Schematic shows the engineered chimeric SRY design. Chimeric SRY 1 (C1) contains the HMG box of human SOX18 and the chimeric SRY 2 (C2) has a Tyr → Phe mutation in box position 72 (position indicated by red bar and full-length numbering 127) as the related clinical mutation in human SRY. **(C)** An SRY chimera (C1 and C2), which is expressed at a similar level to the wild-type protein as assessed by Western blot, activates the *SOX9* gene in CH34 cell line. A *p*<0.05 (Wilcox test) indicates statistical significance for activation (*); (ns) indicates differences between the histogram of Y127F SRY and the chimeric SRY C2 (the *p*=0.20) that are not significant. **(D)** Workflow of molecular dynamic simulation (MD) calculations. Left, Sox18-DNA crystal structure (protein in gray and DNA backbone in orange) with associated crystallographic water are in cyan. Arrows indicate steps in MD calculations, simulations were run for a total of 200 ns. **(E)** Representative ensemble of aligned WT structures (50 in total) over the time course of the MD for SRY bound to the DNA sequence shown. Middle and rightmost panels show isolated protein and DNA ensembles, respectively. Boxed middle panel highlights fluctuations of the minor wing complementary to a region of fluctuations in DNA (boxed in right). Arrow indicates fluctuations of loop 1 in the HMG domain.

To investigate the structural origins of the above biophysical findings, we surveyed the Protein Database (PDB) to obtain a high-resolution co-crystal structure of an homologous SOX HMG box-DNA complex (**Supplemental Table S1**). There are six such structures deposited in the Protein Database with different degrees of resolution and amount of associated crystallographic water molecules (**Supplemental Table S1**). Of the several such structures in the PDB, the highest resolution (1.75 Å) was obtained in crystallographic studies of the Sox18 domain-DNA complex (**Figure 7A**) (25). SRY- and SOX18 HMG-box sequences are aligned in **Figure 7A** with key conserved side chains highlighted in red (tail) or green (minor wing); differences are shaded. An alignment of the human SRY and murine Sox18 HMG boxes is shown in **Figure 2** (above) (**Supplemental Figure S4**). To justify the use of the latter high-resolution structure as a model of the SRY-DNA complex, we first constructed chimeric SRY coding regions in which the SRY HMG box was replaced by the human SOX18 HMG box or its Y72F variant (**Figure 7B**); shown in schematic form are the WT SRY construct (top), Y127F SRY (box position 72), chimera C1 with WT SOX18 HMG box, and chimera C2 with Y127F SOX18 HMG box (bottom).

To evaluate respective gene-regulatory activities, the four constructs were expressed to equivalent levels in rodent CH34 pre-Sertoli cells (14, 75) using a plasmid-dilution protocol to restrict expression level to *ca*. 10^4^ protein molecules per cell (**Supplemental Discussion** in our companion article (14, 36)). A functional read-out was provided by the endogenous *Sox9* autosomal gene, the principle physiologic target of SRY in embryonic differentiation of the bipotential gonadal ridge (42). Quantitative real-time rt-PCR provided an assay for *Sox9* mRNA abundance (**Figure 7C**). Chimera C1 was slightly more active than WT SRY, presumably due to favorable box substitutions at box positions 5 (Val *vs*. Ile) and 13 (Ile *vs*. Met) in accordance with consensus SRY/SOX box sequences (green and bold respectively in **Figure 7A**; see also **Supplemental Figure S2**). Inherited Swyer mutation Y127F (box position 72) causes indistinguishable percent decrements in transcriptional activity in either context. Such corresponding functional perturbations provide evidence that structural relationships in the Sox18 co-crystal structure are pertinent to SRY and its mechanism of transcriptional regulation.

### MD simulations predict Y72-anchored water molecule at DNA interface

The co-crystal structure of the Sox18 box-DNA complex contains 194 water molecules, including at a site connecting the *para*-OH of Y72, the carbonyl oxygen (O=C) of I5 (V5 in the SRY domain; **Figure 7A**) and a DNA phosphodiester group (**Figure 2D**). This water molecule is well positioned to accept a hydrogen bond from the Y72 side chain and donate hydrogen bonds to I5 O=C and the DNA backbone; the latter bifurcates to engage a non-bridging oxygen in the lower strand (3’- TGATCGTAACpAGACCGG-5’ [bold]; inset at left in **Figure 2E**) and the adjoining 3’-ester oxygen in the CpA step (**Supplemental Table S9**). To simulate the solvation of the Sox18 box-DNA complex and its dynamic reorganization, a three-step protocol was implemented as illustrated in **Figure 7D**: *step 1*, removal of the crystallographic waters to leave an unsolvated WT protein-DNA complex; *step 2*, immersion of the complex in a box of 50,000 simulated water molecules (91); and *step 3*, 200-ns MD simulations of the solvated system with periodic boundary conditions. The MD simulations employed GROMACS 2018 (92); (website http://www.gromacs.org). Simulations were repeated for a Y72F variant (obtained by rigid-body deletion of the *para*-oxygen atom of Y72), the free WT domain and the free DNA site. Although the protein-DNA complex undergoes overall rotation and translation in the course of the simulation (curved arrow at right in **Figure 7D**), the L-shaped α-helical structure of the HMG box was well maintained (central two panels in **Figure 7E**). Alignment of a representative ensemble of time points according to “hydrophobic wedge” of major-wing residues in close contact with the DNA (residues M9, F12, M13, and W43) and its core “aromatic anchor” (W15 (60)) revealed greater conformational variability in the minor wing (box in central image in **Figure 7E**) and in the turn between helices α_1_ and α_2_ (arrow). RMSDs are provided in **Supplemental Figure S12**. Conformational variations were also observed in the DNA strands away from the protein interface (at right in **Figure 7E**), including near the distal tail of the HMG box (box).

**Figure 8:**
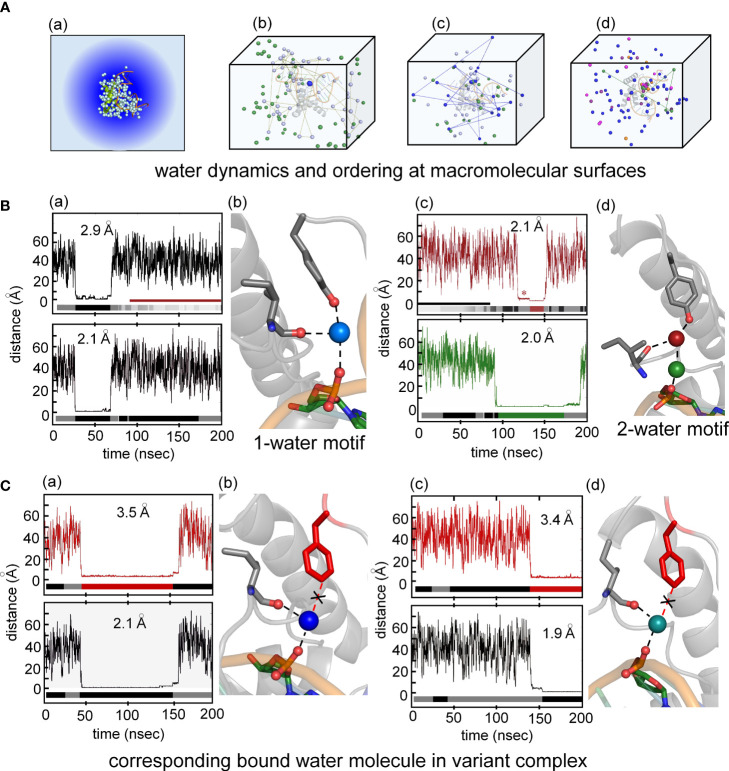
Water-mediated hydrogen at the protein-DNA interface near position 72. **(A)** (a) Crystal structure of the Sox18-DNA complex with associated waters (pale cyan spheres) represented in a water box with spheres of hydration. (b) the positions occupied by three water molecules during MD simulation of 200 ns for Sox18-WT to illustrate motion of bulk water (green and pale cyan). Motion of one of the bulk waters (pale cyan) traced with connecting broken lines. The bridging water between hydroxyl group of Tyr72 and phosphate of DNA tracked in blue color during single water-mediated hydrogen bond between Ile5-Tyr72-DNA. (c) Transition of one water to two water-mediated hydrogen bond involving the backbone carbonyl of Ile5-Tyr72-DNA for Sox18-DNA complex. The bridging single water (blue) in exchange with bulk water (indicated by the connected lines of its trajectory after leaving its bridging position). Over the course of time, another water (green) is accommodated between the interface of Ile5-Tyr72-DNA. (d) Two water-mediated hydrogen bonds, involving water at Ile5-Tyr72 and DNA-water (green). The protein associated waters at the interface during this phase exchange (represented by two different waters entering and leaving the site, magenta and orange). **(B)** (a) Course of a water during the single water-mediated hydrogen bond for Sox18-DNA held by the hydroxyl of TyrY72, the lower plateau is the residency time for this water. Distances shown are for the water hydrogen atom to the acceptor oxygen atom. Water “barcode” showed as alternating colored (black and gray) represent different water molecules that occupy this site at the interface. “Barcode” highlights the single water-mediated hydrogen bond and two water-mediated hydrogen bond phase depicted by the maroon bar. Bottom panel represent trajectory of the same water (top panel) from the DNA where the bridging interaction at the interface. A similar “barcode” for water molecules at the DNA site are shown below the trace. (b) Structural representation of a single water-mediated hydrogen bond at interface of Ile5-Tyr72-DNA of Sox18-DNA complex. The protein and DNA oxygen atoms that form the bridging hydrogen bonds are shows as spheres. The average distance between the water hydrogen atom and respective oxygen atom in each plot is shown for the plateau highlighted. (c) Two water-mediated hydrogen bond at the interface of Ile5-Tyr72-DNA for Sox18-DNA. The Tyr and Ile associated water is in maroon and the DNA water in green. The trajectories of these waters are shown in the plots along with the corresponding “water barcodes.” The asterisk highlights distances close to the *para*-hydroxyl but not yet hydrogen bonded. **(C)** (a) Trajectory plots of a water for the Y72F variant Sox18 complex. The plateau indicates a “long-lived” water close to the *para*-carbon (C_ζ_) of the Phe side chain. The “barcode” below the plot indicates water molecules that are found at this site relative to the Phe side chain for the duration of the simulation. The average distance of the *para*-carbon to the water for the plateau is listed. (b) Plot of the same water molecule as in (a) with respect to the DNA. The average distance of this water to the oxygen atom to its DNA hydrogen binding partner is listed. (b) Structural model of Y72F variant Sox18 complex. The Phe side chain is in red, the Ile side chain and the DNA base that are able to form hydrogen bonds to the water are shown as sticks. (c and d) Plots and models similar to (a and b), highlighting that a single-water motif for the variant simulation is observed through the entire 200 ns.

Particular attention was paid to the dynamics of water molecules in the bulk solvent and at macromolecular interfaces in accordance with general models of hydration shells (panel (a) in **Figure 8A**) (93). Trajectories of individual water molecules thus demonstrate transitions from rapid nonlocal dynamics in bulk solvent (on the picosecond time scale) to occupancy of binding sites at the surface of the protein or DNA (on the nanosecond time scale) followed by release to the bulk solvent. Examples of three such transitions are shown in panels b-d in **Figure 8A**. This protocol enabled assessment of the Y72-related water binding site (**Figure 8B**) and its counterpart in the Y72F simulation (**Figure 8C**). Remarkably, starting from a box of 50,000 random water molecules, long-lived occupancy of the Y72-related site began within 10 ns. In the WT simulation two water-mediated motifs bridge the *para*-OH of Y72 to the DNA backbone. The first (designated the “1-water motif”) recapitulates the single crystallographic water molecule (blue) engaged in a network of hydrogen bonds involving Y72, I5 and the CpA phosphodiester group (panels a and b in **Figure 8B**). The second (“2-water motif”) utilizes a pair of adjacent water molecules (maroon and green) to make an analogous bridge (panels c and d in **Figure 8B**). In panels a and c (**Figure 8B**) trajectories are illustrated for individual water molecules leaving bulk solvent (irregular distances from 20-60 Å at top), occupying the Y72-related site for 15-100 ns and then rejoining the bulk. Beneath each trajectory is a gray-black “bar code” representing changes in the identity of the occupying water molecule; seven different water molecules thus occupied the Y72-related bridging site in the course of the 200-ns WT simulation. In the 2-water motif, the specific water molecule in the lower site can transition to the upper site (red asterisk in upper panel of **Figure 8B(c)**) and then exit to bulk solvent.

Surprisingly, MD simulation of the Y72F box-DNA complex preserved essential features of the protein-DNA interface. In particular the variant trajectory exhibited native-like minor-wing core packing, extending even to long-lived occupancy of an analogous water-binding site as defined by hydrogen-bond donors I5 C=O and the CpA phosphodiester group (*i.e*., in the absence of the *para*-OH donor; **Figure 8C**). A partial water-mediated bridge was thus maintained between the DNA backbone and the minor wing’s N-terminal β-strand—but not to the C-terminal basic tail (black “X” in **Figure 8C(b)**). Only a corresponding 1-water motif was observed. Respective durations of occupancy by individual water molecules are similar to that in the WT simulation (bar codes in panels a and c of **Figure 8**). The position of the bound water molecule in the variant site is shifted by *ca*. 1.3 Å relative to its position in the WT simulation. On alignment of respective major wings, the mean position of the *para*-carbon of F72 is shifted by 3.1 Å relative to the *para*-carbon of Y72. In an effort to correlate stable occupancy of bridging water molecule with relative gene-regulatory activities (**Figure 1A**), we extended these simulations to a set of low-activity variants (Tyr→Ala, Gln and His; **Supplemental Table S2**). The variant side chains in each case only short-lived neighboring water molecules were observed (i.e., on a subnanosecond time scale; see **Supplemental Figure S13** and Discussion below). Such structure-activity relationships suggest that the precise positioning of the side-chain hydrogen-bond donor (enforced by side-chain structure; **Figure 2D**) is critical to formation of a water-mediated clamp.

**Figure 9:**
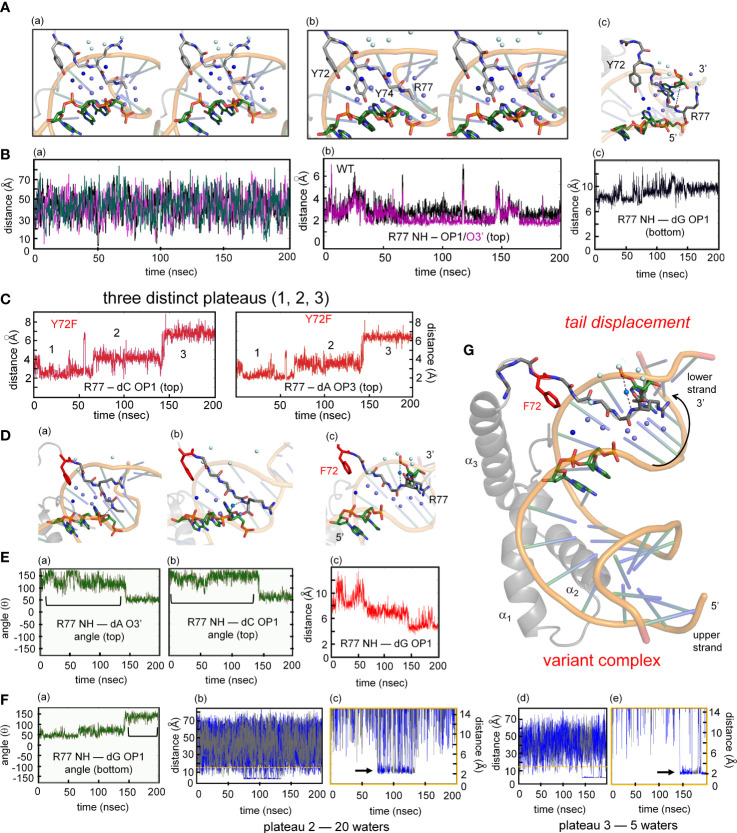
MD simulations of protein-DNA interfaces. **(A)** (a) Stereoview of the water network of WT SOX18-DNA complex during single water-mediated hydrogen-bond motif with the longest occupancy time in dark blue. The Tyr72-DNA bridging water is in dark blue, other interfacial waters in light blue and the bulk water in pale cyan. (b) Stereoview of the tail highlighting the Y72 and the side chain of Y74. The tyrosine at position forms a hydrogen bond with the nucleobase. (c) Structural view of the two water-mediated hydrogen-bond motif and associated interfacial waters at the end of the molecular dynamic simulation. The broken line represents the distance between Arg77 amide to an oxygen atom of a cytosine phosphate on the lower strand (chain C C7 in Sox18 box-DNA crystal structure). **(B)** (a) The course of five water molecule close to Arg77 amide at different time points during the MD simulation. Each of the water molecules show Brownian motion with very short occupancy times. (b) Trajectory of the WT Arg77 amide to oxygen atoms on a cytosine phosphate represented in purple and black. (c) Trajectory of WT Sox18 Arg77 amide to guanosine (chain B G14) of DNA. **(C)** Course of Arg77 amide and OP1 and O3’ with stepwise three distinct distance profile. **(D)** (a-c) Representative structural views of the variant-DNA complex from each of the distinct three distance phase of Arg77 amide and DNA. Long-lived water is observed (dark blue) and associate interfacial waters are in depicted in light blue and bulk water in pale cyan in step 1 (a), step 2 (b) and step 3 (c). The broken line represents a direct hydrogen bond between the amide of Arg77 and an oxygen of the DNA backbone. In panel (b) a water mediates the interaction between the amide of R77 and C7 (chain C), in (c) the water-mediated hydrogen bond between the amide of R77 has switched to G14 (Chain B). Panels a-c is representation of the diffusion process of bulk water at the interface in variant. **(E)** (a-b) Hydrogen-bond angle between Arg77 amide-O3’(a) and OP1(b) over the course of MD simulation for variant complex. Bracketed regions represent appropriate angles for hydrogen bond formation. (c) Trajectory of the amide of Arg77 to OP1 of a bottom strand guanosine indicating the shift of the tail from the top strand towards the bottom strand. **(F)** (a) Angle profile for amide Arg77 and OP1 of the guanosine with bracket indicates an appropriate angle for hydrogen bond. (b-e) Trajectories of the different water molecules that mediate hydrogen bond between amide of Arg77 to the DNA at plateau 2 (b) and plateau 3 (d) with expansion of respective plateau 2 (c) and 3 (e). Black arrow in each expanded panel highlights bridging water between R77 and the DNA, alternating blue and gray colors represent a different water that participates in bridging interaction. **(G)** Structure of the variant Sox18-DNA complex at the end of the MD time scale. The extended water network is shown with the same blue hue scheme. Positioning of the tail, especially the tail residue R77 has switched to the opposite strand (lower) indicated by a curved arrow. The water-mediated hydrogen bonding of R77 to the upper strand is shown as dashed lines.

The MD simulations thus pose a seeming paradox. Although accelerated dissociation of the variant box-DNA complex is its most prominent experimental feature (as uncovered by stopped-flow FRET), what atomic-level mechanisms might underlie this salient finding? Toward the end of the simulation the last tail residue (R77) of the variant complex was observed to completely detach from its original position and form a water-mediated hydrogen bond to the opposite strand (arrow in **Figure 9G**). This coincided with more water molecules present at the protein tail-DNA interface (**Figures 9D, G**) compared to the wild type. We speculate this is an initial step in protein disassociation from the DNA. The present MD simulations are limited in duration (200 ns) and so cannot capture protein-DNA dissociation and rebinding on the time scale of 0.1-10 seconds. Detailed analyses of transmitted perturbations in the simulated structure and solvation of the F72-associated basic tail are, nonetheless, feasible as presented in the Discussion (**Figure 9**; below). Although speculative (and so not presented here as Results), these aspects of the MD simulations define a potential pathway between *loss of the Y72 para-hydroxyl group* and *detachment of the tail*. Intended as a working hypothesis, this proposal may guide design of future experiments.

Although for technical reasons our MD simulations exploited an homologous high-resolution co-crystal structure (25), control simulation of the solution structure of the SRY box-DNA complex (3) suggests the generality of our results. Differences in DNA structure in the NMR-based structure—especially in the conformation of the phosphodiester group nearest Y72 (**Supplemental Figure S14**)—led to no Y72-mediated anchoring of a DNA-linked water molecule for the first 160 ns of the MD trajectory. However, by this simulated time point the SRY-bound DNA fortuitously reorganized to provide a favorable orientation of the critical phosphodiester group. For the final 40 ns of the 200-ns simulation, the *para*-hydroxyl group of Y72 then stabilized a water molecule linked to DNA and the carbonyl group of V5 on the nanosecond time scale (bar code in **Supplemental Figure S14**). This one-water motif in the extrapolated SRY-DNA complex is essentially identical to the corresponding feature of the Sox18 box-DNA complex *in crystallo* (25) and in the solvated MD trajectory.

## Discussion

Hierarchical gene regulation, a general feature of metazoan development, is remarkable for its robustness (94). Designated Waddington’s Principle (WP) (69, 95, 96) (see *Box 1* in preceding article in this issue (14)), such robustness is associated with canalized pathways of morphogenesis, ordinarily ensuring functional outcomes despite genetic variation and environmental fluctuations. The molecular-genetic origins of WP may reflect the set points of individual regulatory steps or the overall topology of regulatory networks, in either case buffering phenotypes to incremental changes in component biochemical parameters (97). Vertebrate sex determination honors WP in the breach, as evidenced by the paradoxical fluidity among sex-determining mechanisms within individual genera (98, 99). Inherited Swyer syndrome provides a model of a non-canalized pathway of human organogenesis (36, 56, 100). The tenuous transcriptional threshold of mammalian sex determination, as inferred from studies of Swyer syndrome and intersexual mouse phenotypes (101), defines molecular mechanisms at the borderline of embryonic Sertoli-cell specification.

SRY and its orthologues provide the testis-determining factor only in therian mammals (9), despite broad conservation of downstream sex-determining genes and hormonal mechanisms in vertebrates (44). Emergence of evolutionary novelty at the “top” of regulatory hierarchies (also observed among invertebrates (102)) suggests that sex-determining pathways grow backwards, *i.e*., from bottom to top (103, 104). Rapid innovation is enabled by co-option of regulatory genes from other signaling systems and by degeneration of non-recombining sex chromosomes (105, 106). Such co-option is rapid on an evolutionary time scale (107, 108).

Swyer syndrome arises from mutations in the *SRY* gene (100, 109, 110). The majority of clinical mutations occur *de novo* in paternal spermatogenesis and are often associated with marked perturbations in the specific DNA-binding properties of the HMG box (60, 109). An example is provided by substitution of an aliphatic “cantilever” side chain (I68; box position 13) by a short, polar side chain (Thr) unable to insert between DNA base pairs (**Supplemental Figure S15A**) (21, 111, 112). Inherited Swyer syndrome is by contrast rare, having been observed to date in only seven families globally (**Supplemental Table S10**). Their pedigrees provide models of a developmental switch poised at the edge of ambiguity: a variant TF compatible with either male or female somatic phenotypes. Although it is not known whether the variable developmental outcome is determined by genetic context (*e.g*., modifier genes differing between the father and mother) or due to stochastic gene expression (113), each such family defines an experiment of nature in principle probing molecular mechanisms at the borderline of SRY function (36, 56, 57). In previous studies we have characterized inherited Swyer mutations partially impairing either nuclear import or nuclear export (V60L and I90M, respectively (36); **Supplemental Figure S15B**), together establishing that nucleocytoplasmic shuttling is required for robust operation of the male switch in gonadogenesis (34). Such shuttling enables native phosphorylation of a putative protein-kinase A (PKA) site N-terminal to the HMG box (36). Yet another inherited mutation [F109S; box position 54 (57)] destabilizes the protein’s hydrophobic core (**Supplemental Figure S15C**), leading to proteosomal degradation and in turn enabling an estimate of the threshold number of TF molecules per pre-Sertoli cell necessary to assure testis formation (36).

### Inherited mutation Y72F preserves native-like structure

In this and the preceding article in this collection (a special Research Topic in Structural and Molecular Endocrinology) (14), we have focused on Swyer mutations at a seemingly “unremarkable” site in the HMG box: the junction between its minor wing and basic tail (15–17). Tyr is invariant at this site (box position 72) among mammalian Sry orthologues and broadly conserved among SOX factors (35). Two *de novo* mutations (Y127H and Y127C in intact human SRY) were found, as expected given the clinical context, to markedly impair SRY-directed transcriptional activation of principal downstream target gene *SOX9* in cellular models (14). Respective mechanisms nonetheless differed, in one case *via* a direct destabilization of the box-DNA interface (His) and in the other case *via* mutational destabilization of the protein to cellular degradation (Cys). In the latter case, chemical inhibition of the proteasome largely restored transcriptional activation of principal target gene *Sox9* (14) in association with rescue of testis-specific enhancer occupancy (**Figure 3C**). It would be of future interest to investigate structural and cellular mechanisms underlying these respective perturbations

The present study sought to address how and why inherited allele Y127F (box position 72) (14) may be compatible with the divergent phenotypes of the proband (infertile XY female) and her father. We hypothesized that such variable penetrance ultimately reflects perturbed biophysical properties of the variant HMG box. Biochemical studies uncovered only subtle perturbations with retention of native-like specific DNA-binding and bending (14). Although posing a genetic paradox, the apparent absence of biochemical changes is in accordance with the chemical similarity between these isosteric aromatic side chains (**Figure 3A**) and their seeming structural interchangeability at the box-tail junction. In the solution structure of the WT SRY HMG box-DNA complex (3), Y72 contributes to packing within the mini-core of the minor wing (a cluster of side chains including V5, H65 and Y69; respective residues 60, 120 and 124 in intact human SRY). The *para*-hydroxyl group of Y72 lies at a protein surface within a crevice between the basic tail and DNA (**Figure 1B**; **Supplemental Figure S16**). To our knowledge, functional annotation of this conserved side chain and its *para*-hydroxyl group has not previously been described.

The native-like properties of Y127F SRY in cell culture and of the corresponding Y72F HMG box *in vitro* [see companion study (14)] rationalized the father’s phenotype as a fertile male—*but what of the XY daughter?* To address this question, we undertook biophysical studies of the variant domain-DNA complex and extended these studies through MD simulations (63, 114). In accordance with the prior low-resolution CD studies (14), comparative ^1^H-^15^N 2D-HSQC “footprint” NMR spectra of the WT and variant domains are essentially identical (**Figures 5A, B**). Conversely, binding of the WT or variant domain to a specific DNA site led to similar, marked changes in the downfield ^1^H-NMR resonances of DNA imino resonances (56, 115). Subtle nonlocal changes in protein- and DNA chemical shifts were nevertheless observed, providing evidence for transmitted adjustments at the box-DNA interface. Whereas DNA-dependent stabilization of the HMG box is maintained, Y72F causes small distributed changes in secondary chemical shifts in both macromolecules.

### Inherited mutation Y72F preserves near-native DNA bending

Evidence of enhanced nonlocal conformational fluctuations was provided by time-resolved FRET and distance-distribution analysis (33, 56, 58, 61). These studies employed a 15-bp DNA duplex (*ca*. 1.5 double-helical turns) whose respective 5’-ends were flexibly linked to a fluorescent donor or acceptor (**Figure 4A**). Whereas sharp DNA bending was maintained in the variant box-DNA complex in accordance with prior PGE-based estimates of DNA bend angle (14), a slight, yet reproducible broadening was observed in the end-to-end distance distribution (**Figure 4C**, right) in association with reduction in energy-transfer efficiency and slight shift in position of the EEDD peak (**Supplemental Table S3**). The increased width of the time-resolved FRET-based distance distribution in the WT box-DNA complex relative to the free DNA [itself reflecting primarily the flexibility of the hexanyl-linked fluorescent probes (33, 61)] suggests that the native complex exhibits a baseline range of DNA bend angles rather than a single angle. It would be of future interest to consider multi-conformer models of the WT and variant domain-DNA complex (116, 117).

The present analysis was based on a single-population model. We note that the dependence of the probability of excitation energy transfer on the distance between 5’-probes is weaker at distances outside the range [R_0_ ± 0.5R_0_] (where Ro represents the Förster distance). Thus, the present method of EEDD fitting imposed *concurrent changes in long- and short-distance gaussian tails*, an assumption that may miss subtle features of a variant EEDD. Indeed, due to the stiffness of the DNA double helix on a length scale of 15 bp, we imagine that near-conservative mutations in the HMG box (such as Y72F or V5L (56); **Supplemental Figure S7**) would have asymmetric effects on EEDD shape, thereby favoring longer end-to-end distances (i.e., less bent DNA conformations) with little effect on the shortest end-to-end distances (more sharply bent DNA conformations). Asymmetric modeling of such a variant EEDD, considered as a perturbation of the WT distribution, might yield more robust statistical assessment. Such biophysical methods development promises to provide insight into the precision or imprecision of protein-directed DNA bending, which is likely to have biological implications for mechanisms of architectural gene regulation (see below) (118).

### Mutation perturbs kinetics of protein-DNA binding

In contrast to the native-like structure of the Y72F HMG box and its specific DNA complex, a marked acceleration of protein-DNA dissociation was observed in stopped-flow FRET studies (**Figure 6**). Observed *off*-rates and inferred *on*-rates are given in **Supplemental Tables S7**, **S8**. Changes in respective *on*- and *off*-rates largely compensate, giving rise to near-native protein-DNA affinity (14). Similar kinetic perturbations with compensatory changes were observed in FRET-based studies of Swyer mutations V60L and M64I (box positions 5 and 9) (36, 119). The former substitution alters the minor-wing mini-core adjacent to Y72 whereas the latter alters packing of the motif’s “hydrophobic wedge” (14) within the DNA minor groove near Y72. These variant box-DNA complexes also exhibited a slight increase in FRET-derived end-to-end distance distributions in association with subtle attenuation of secondary ^1^H-NMR imino chemical shifts in the bent DNA site (56, 60, 120). The shared features of these variant box-DNA complexes suggest that the *kinetics* of protein-DNA binding is more sensitive to subtle perturbations in steric complementarity than are *thermodynamic* affinities. Native-like affinities in these cases may be maintained through entropy-enthalpy compensation (EEC) (121). Calorimetric evidence for EEC has been described in studies of an homologous SOX domain.

We speculate that subtle enhancement of local and nonlocal conformational fluctuations in the Y72F box-DNA complex (as inferred from time-resolved FRET and NMR studies) is connected to accelerated protein-DNA dissociation. This model posits that conformational fluctuations in the bound state facilitate access to a transition state, enabling more efficient free-energy barrier crossing en route to dissociation. Modest changes in barrier height are sufficient to account for the stopped-flow results: a fourfold increase in *off*-rate at 37 °C predicts a 0.85 kcal/mole reduction in free-energy barrier (ΔΔG*). Because binding of the specific HMG box is slow relative to classical diffusion-limited protein-DNA binding (56), subtle perturbations in the dynamics of the variant box-DNA complex or its solvation (122) may also mitigate kinetic barriers to dissociation. Such considerations motivated the present MD simulations (**Figures 7D, E**, **8**) and their interpretation (**Figure 9** below). We acknowledge that such ground-state perturbations may underestimate the extent of transition-state perturbations.

### MD simulations of tail-DNA interface: water-mediated clamp hypothesis

Because the solution structure of the SRY HMG box-DNA complex lacked definition of bound water molecules, MD simulations were undertaken of a homologous high-resolution co-crystal structure of a representative SOX-box-DNA complex (Sox18) (25). Of deposited SOX-DNA complexes in the PDB, this structure is the highest in resolution (1.75 Å) and contains the largest number of crystallographic water molecules (194 assigned in the electron-density map; see **Supplemental Table S1**). Of particular interest is a water molecule hydrogen-bonded to the *para*-OH group of Y72 as a bridge to (a) the DNA backbone (the phosphodiester group of the CpA step in the lower strand; inset at lower right in **Figure 7E**) and (b) across the minor wing to the carbonyl oxygen (O=C) of I5 (**Figure 2**). An analogous Y72-bridging water molecule occurs in the crystal structures of the SOX11-DNA complex (73) and the SOX2-POU-DNA ternary complex (26) (**Supplemental Figure S17**). Mean hydrogen-bond lengths are given in **Supplemental Table S9**. Our MD simulations of the Sox18 box-DNA complex in a box of 50,000 water molecules (randomly placed; **Figures 7D**, **8A**) demonstrated that a corresponding bridging water molecule quickly occupies this site (within 5.86 ns) and is long lived on the time scale of 10-100 ns (**Figures 8B(a, b)**); an alternative bridge motif comprises two hydrogen-bonded water molecules (**Figures 8B(c, d)**). Surprisingly, a similar but not identical long-lived water binding site at the protein-DNA interface was occupied in the MD simulation of the Y72F box-DNA complex, defined by the three oxygen atoms (the carbonyl oxygen of I5 and two oxygen atoms in the CpA phosphodiester group) in the absence of the *para*-OH of residue 72. Although the aromatic side chains of Y72 and F72 are in similar structural environments, reinforced by corresponding packing of respective minor-wing mini-cores (**Figure 2** and related long-range NOEs in **Figures 5C, D**), a small shift (1.3 Å) was observed in the position of the I5/DNA-bound water molecule.

How might removal of the Y72 *para*-OH lead to accelerated dissociation of the SRY box-DNA complex (**Figure 6B**)? To explore this question within the 200-ns native-state MD simulations, we were guided by a previous comparative study of SRY domains with either an intact tail (box residues 69-85; sequence YPNYKYRPRRKAKMLPK) or truncated tail (residues 69-78; YPNYKYRPRR) (33). Subtle perturbations observed on tail truncation resemble the present findings: (i) accelerated box-DNA dissociation disproportionate to near-native affinity; (ii) analogous changes in ^1^H-NMR imino chemical shifts; and (iii) EEDD broadening in time-resolved FRET analysis of protein-directed DNA bending (**Supplemental Table S3**) (33). Based on this analogy, we undertook an analysis of tail-DNA interactions and solvation in the WT and variant MD simulations. Our analysis had two parts, focusing first on the proximal tail (residues 69-74) and then on the distal tail (residues 75-77; **Figure 9** and **Supplemental Figures S18, S19**). We regard the findings below as motivating a *working hypothesis* (and not as Results) given differences in sequence and length between the SRY tail (17 residues) and SOX18 tail present in the crystallized fragment (HPNYKYRPRR in **Figure 7A**; 10 residues) (25).

The four basic side chains in the SOX18 tail engage in general electrostatic interactions with the DNA minor groove rather than precise charge-stabilized hydrogen bonds to the DNA backbone. Directional short-range interactions are mediated by the side chain of Y74 (proximal tail; **Figure 9A** and **Supplemental Figures S18, S19A, C**) and the main-chain amide group of R77 (distal tail; **Figure 9D** and **Supplemental Figures S19D**). These interactions are direct and not water-mediated. The *para*-OH group of Y74 engages in both base-specific and phosphate-specific hydrogen bonding. Their stability (in the SRY HMG box) led to the unusual ^1^H-NMR observation of a downfield Y74 *para*-OH resonance in slow exchange with the solvent water resonance (33). Although in the WT and variant MD simulations, the I5 side chain exhibits conformational flexibility within the minor-wing (as probed by H69-I5 packing at left in **Supplemental Figure 19B**; representative distances shown at black and red at right), the starting crystallographic position and interactions of the Y74 side chain are consistently maintained in the DNA minor groove (black and red in **Supplemental Figures S18**, **S19C**). Immediate engagement of Y74 at the DNA interface thus anchors the proximal tail irrespective of the *para*-OH of Y72. A striking difference is observed in the distal tail. Whereas the WT trajectory preserves the bifurcated R77 peptide NH-DNA hydrogen bonds (**Figure 8E**), the Y72F trajectory is remarkable for stepwise breakage of this hydrogen bond in association with successive displacement of the distal tail (plateaus 1-3 in **Figure 9C**). Maintenance of the proximal tail and stepwise displacement of the distal tail in the variant MD simulation is shown as plots of respective tail-specific C_α_ distances to the closest DNA phosphodiester group in the starting structure (**Supplemental Figure S20**; the three plateaus shown in panel (d)) correspond to those in **Figure 9C**.

**Figure 10:**
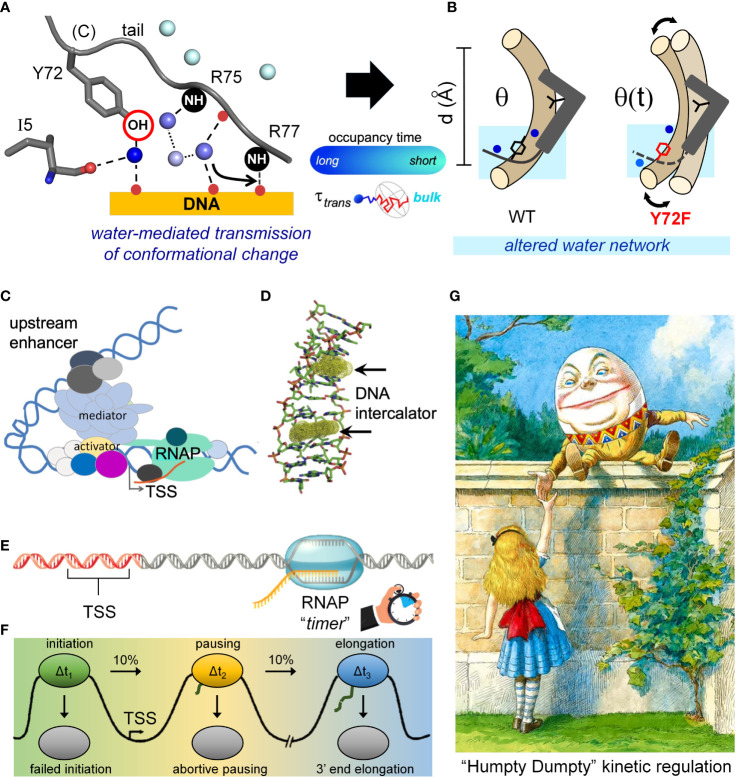
”Humpty Dumpty” kinetic model of transcriptional regulation. **(A)** Schematic diagram of the Tyr72-DNA-water associated network at the interface of the tail of the HMG domain and the DNA. The long-lived bridging water is in dark blue, waters with shorter residency lifetimes are in increasing blue hues and bulk water in pale cyan. Color scale is described by gradient blue bar representing “occupancy time.” The hydroxyl (-OH) is highlighted, oxygen atoms of the DNA and Ile5 that participate in hydrogen bonds are shown as spheres. Peptide amide groups of Arg75 (R75) and Arg77 (R77) are shown as black “NH” spheres and oxygens of the carbonyl and phosphate backbone shown as red circles. Dashed bonds indicate longer occupancy of interaction compared to dotted lines. Arrow indicates site of broken direct protein-DNA interaction in the variant domain by intervening water. Conformational changes in the water network when comparing the WT and variant domains affect dynamic fluctuations of the bent DNA-protein complex (indicated by black large arrow). **(B)** Altered water network in the variant domain leads to partial tail displacement and increased dynamic fluctuations. Static and dynamic models of the protein-DNA complexes. Human HMG domain is represented by gray “L”-shape with intercalative Ile residue side chain indicated. C-terminal tails are shown as solid (WT) and dashed (variant) lines. The bridging waters associated at position 72-DNA interface in each complex are shown in dark blue. Intervening water in the variant complex during partial tail displacement is shown in light blue. **(C)** Model of enhanceosome formation including bending of the DNA by protein mediators. DNA bending by architectural transcription factors upstream of the transcriptional start site (TSS) may facilitate interactions with activator factors which in-turn may recruit and/or stabilize transcriptional machinery for transcription of target genes. **(D)** DNA bending can be achieved by total or partial intercalation of amino side chains between strands of DNA. Depicted are the intercalation sites on the DNA for TATA-box binding protein (TBP), which intercalates a pair of symmetrical Phe side chains (sites are indicated by outlined dots) during DNA bending (123). **(E, F)** Model of RNA polymerase pausing and kinetic regulation of transcription. Proposed are three states of paused RNAP in the metazoan gene regulation. In each phase the estimated amount (10%) of all RNAP-DNA complexes that move into the next phase is shown. Within each stage the duration of the RNAP pausing was estimated and represent Δt_1-3_ of each phase (124). **(G)** Classic tale of Humpty Dumpty, which represents a model in which the formation of a multi-protein transcriptional-related complex and its various paused states in transcriptional initiation regulates gene transcription. As is the case for the variant human SRY, we propose that the variant HMG domain is unable to create kinetically stable enhanceosome complexes to same extent as WT. The inability to form kinetically stable enhanceosome complexes akin to Humpty Dumpty’s inability to be “put back together” after having fallen from the wall [image from (125)].

Displacement of the distal tail in the Y72F MD simulation is associated with a switch in orientation of the R77 main-chain NH group from a *direct hydrogen bond* to the ApC phosphodiester group in the lower strand (WT; **Figure 9A**) to a water-mediated hydrogen bond to the GpG phosphodiester group in the upper strand (variant; **Figure 9D**). Whereas the latter interaction is never observed in the WT simulation (**Figures 9A, B(b)**), stepwise displacement of the distal tail in the Y72F simulation leads to its maintenance in plateau 3 (**Figures 9C, G**). The transition from plateau 1 to plateau 2 breaks the native R77 main-chain NH-DNA hydrogen bond but without long-lived water molecules (**Figure 9D(b)**); the ensuing transition from plateau 2 to plateau 3 enables formation of a long-lived water-mediated hydrogen bond to the opposite DNA strand (**Figures 9E, F (b-e)**). In a 100-ns MD simulation of the Y72H HMG-DNA complex we also observed tail displacement and reorientation of the R77 side chain (**Supplemental Figure S21**). We propose that these alternative tail conformations and tail-DNA interactions accessible to the Y72F domain on the time scale of 0-200 ns, although visualized within an otherwise native-like structure, foreshadow kinetic steps in the pathway of protein-DNA dissociation and an altered diffusion pathway for bulk water (**Figures 9D(a-c), G**). This hypothesis thus envisions that the kinetic instability of the variant complex reflects the transmitted effect of altered tail solvation (**Figure 10A**).

Water-mediated transmission of conformational change in the SRY box-DNA interface relates the dynamics of solvation to the kinetics of protein-DNA dissociation (central panel between **Figures 10A, B**). In the future this model may be tested through rigorous crystallographic and heteronuclear NMR studies. Of compelling interest would be high-resolution co-crystal structures of WT and Y72F SRY box-DNA complexes, optimized in each case to enable visualization of bound water molecules as in the structure of the Sox18 box-DNA complex (25). Such structures would inform how the mutation affects the position, interactions and solvation of the tail, including the position of the predicted bridging water molecule near Y72 or F72 and the interactions of the R77 main-chain NH. Of complementary interest would be isotope-directed NMR studies of interfacial water molecules (126–128). Mutation-associated changes in the dynamics of the protein and DNA may also be probed through comparative analysis of residue-specific and bp-specific residual dipolar couplings (50). The present ^1^H-NMR and time-resolved FRET features of the respective protein-bound DNA sites predict enhanced conformational fluctuations in the variant box-DNA complex (**Figure 10B**), which may be most marked near the tail (dashed line at right).

The water-mediated clamp hypothesis is supported by the correlation between functional studies of SRY variants and tail solvation as observed in a systematic set of MD simulations, also performed in the context of the high-resolution Sox18 box-DNA co-crystal structure (**Supplemental Table S2**) (25). In the case of Y72A, Y72H and Y72Q, marked attenuation of SRY’s gene-regulatory activity (**Figure 3B**) and testis-specific enhancer occupancies (**Figure 3C**) are associated with the absence (Y72A) or subnanosecond exchange (Y72H and Y72Q) of water molecules near the variant side chain. Although the imidazole NH/N atoms of His72 and the carboxamide function of Gln72 can in principle participate in hydrogen bonds to solvent water molecules, in the MD simulations no water-mediated bridge to the DNA or across the minor wing to the carbonyl oxygen of box residue 5 was observed. Y72A, Y72H and Y72Q may also perturb aromatic-aromatic interactions in the native complex, a prominent feature of the minor wing (Y72-Y69) and the tail-DNA interface (Y72-Y74). Because SRY-directed DNA bending is most severely perturbed in the Y72A complex (**Figures 3D, E** **)**, we speculate that such “Ala shaving” disrupts packing of V5 within the mini-core of the minor wing and that such disruption in turn perturbs multiple box-DNA contacts. Although minor-groove deformation by the domain’s hydrophobic wedge (conserved major-wing box residues 9, 12, 13 and 43) may remain nativelike, it would be of future interest to interrogate nonlocal minor-wing-specific NOEs in the Y72A complex. Loss of such contacts would be likely to propagate to perturb packing of Y74 at the DNA surface and disrupt the main-chain hydrogen bond by R77.

The pivotal role of Y72 in organizing the protein-DNA interface was overlooked in a previous MD study of the SRY-DNA complex (129). However serendipitous its present recognition, we anticipate that the Y72-anchored clamp motif will generalize to metazoan SOX domains as a conserved molecular feature of architectural gene regulation (22). In addition, because Y72 is uncommonly substituted by Trp among a specific SOX homologue (SOX30; **Supplemental Figure S2**), we predict that the indole ring may likewise stabilize the structure of the minor wing while its side-chain NH group provides an alternative anchor point for a bridging water molecule. The plausibility of this hypothesis was supported by MD simulation of the Y72W Sox18 box-DNA complex (**Supplemental Figure S22**). This simulation predicts an equilibrium among three modes of binding wherein the indole NH either anchors an homologous single water-mediated clamp (*model 1*), anchors a two-water bridging motif (*model 2*) or directly contacts the carbonyl oxygen of box residue 5 (*model 3*); the latter mode was observed in the solution structure of Lef-1 box-DNA complex with otherwise unrelated tail (42). It would be of future interest to compare the structure and function of Y72W and Y72F SRY variants by NMR or crystallography. Because in the SOX2-POU-DNA ternary complex a neighboring tail residue (D71) contributes to the SRY-POU interface (43), it is possible that Y72W in SOX2 repositions D71, in turn perturbing this interface and hence target gene regulation. Conservation of aromatic side chains at box position 72 in the Lef1/TCF-1 subclass of specific HMG boxes (W>Y to the exclusion of F or H; **Supplemental Figure S2**) suggests extension of this model to an ancestral metazoan superfamily (28). This proposal could be tested through comparative studies of corresponding W72Y and W72F Lef-1 variants (42). We imagine in general that conserved sequence elements in DNA-binding motifs are associated with conserved patterns of functional solvation.

Beyond consideration of box position 72, we envisage that impaired tail-mediated clamping of the SRY HMG box provides a shared biophysical mechanism underlying a diverse class of Swyer mutations (**Supplemental Table S9**). An example is provided by subtle aliphatic substitutions adjoining Y72 in the minor wing: V5L and V5A [inherited Swyer mutations V60L and ovotestis-associated mutation V60A (130–133)]. Biochemical and biophysical studies of the variant HMG boxes and box-DNA complexes demonstrated native-like structure (56, 134) with accelerated protein-DNA dissociation (56). Respective perturbations uncovered by ^1^H-NMR and time-resolved FRET features are similar to those described here, including in pattern of imino chemical shifts and EEDD broadening (**Supplemental Figure S7**). We speculate that the clinical mutations at position 5 alter the position of Y72, in turn perturbing its bridging water molecule and an associated hydrogen-bond network. A more marked perturbation in the position of Y72 and overall tail-DNA clamping would be expected due to *de novo* Swyer mutation P70L (52) and several other clinical mutations as summarized in **Supplemental Table S11**. An analogous biophysical mechanism is likely to underlie diverse clinical phenotypes due to mutations in SOX genes (**Supplemental Table S12**).

It would be of future interest to interrogate the contributions of solvation to SRY box-DNA interactions through the use of isothermal titration calorimetry (ITC) and differential scanning calorimetry (DSC). Elegant such use of these techniques has been described in studies of the SOX5 HMG box by Privalov, Crane-Robinson and coworkers (71, 135). These studies suggested that the enthalpy and entropy of association of this domain (as a minor-groove DNA-motif) are more positive than as typically observed in calorimetric studies of major-groove-binding motifs. Such findings may reflect dehydration and release of counterions on complexation. Comparative ITC studies of WT and variant SRY box-DNA containing mutations in the tail binding might resolve the contributions of changes in solvation enthalpy and solvation entropy (122). Analogous ITC studies of variant zinc fingers have highlighted how formation of mutation-associated packing defects can lead to a solvation-dependent thermodynamic signature (136, 137). These general thermodynamic issues notwithstanding, individual water molecules bridging a specific protein-DNA interface can contribute to sequence recognition beyond the general thermodynamic effects of solvation entropy and solvation enthalpy (41, 65–68). The paradigm of indirect readout of B-DNA, first observed in crystallographic studies of the prokaryotic Trp repressor (65), was generalized to clusters of bound water molecules in homeodomain-DNA complexes (127, 138). Although to our knowledge classical water-mediated interactions have not been described in the bent SRY-DNA complex, this biophysical paradigm would be extended by the proposed kinetic stabilization of the specific complex by a tail-associated water network. Interestingly, uncovering the cryptic function of this network emerged through serendipity: *via* studies of a rare genetic syndrome—due to a subtle inherited mutation at a site of enigmatic conservation—rather than *via* the original structural studies (3, 23).

### Kinetic control of architectural gene regulation

In this and our companion article in this thematic collection (14), we have provided evidence that the transcriptional potency of SRY, as assessed in immortalized cellular models of the embryonic pre-Sertoli cell (36, 57, 75), correlates with the lifetime of the box-DNA complex (*k*_off_) and not its specific affinity (K_d_) (90). We imagine that this lifetime *in vitro* determines in turn the cellular lifetime of one or more SRY-dependent and testis-specific enhanceosomes in far-upstream region of the SOX9 gene (**Figure 1D**) (5, 6, 76). Although functional enhancer DNA elements have been identified (*TES*/*TESCO* and *Enh13*; conserved between rodents and humans), protein components of SRY-dependent enhanceosomes are not well characterized (**Figure 10C**). The putative presence of other developmental TFs, such as WT1 and SF1 (6, 76), has been inferred for neighboring DNA binding sites (**Figure 1E**). The present correlation between stopped-flow FRET-based studies of box-DNA dissociation and target gene-regulatory activity in cellular models suggests that dissociation of SRY (and so relief of a strategic DNA bend (139)) is rate-limiting for disassembly of testis-specific enhanceosomes as multiprotein-DNA complexes. We envision that the cellular lifetime of an enhanceosome determines the burst frequency of transcription (140, 141) through multiple rounds of transcriptional initiation (142). Kinetic control of SRY potency is reminiscent of the correlation between the cytotoxic activity of a DNA intercalating drug and the lifetime of intercalation (**Figure 10D**) (143, 144).

Kinetic regulation of biological processes within cells was first framed in relation to the seeming paradox of high fidelity in processive reactions, such as DNA replication and DNA-dependent RNA transcription (145, 146). At the price of process time and energy consumption, kinetic mechanisms of proofreading could reduce errors below that expected at equilibrium, *i.e*., as naively predicted by the small differences typically observed between correct and competing reactant binding free energies—ideas that have found broad application (147). For example, implicit in the regulation of the complex multi-step process of transcription (formation of a preinitiation complex, initiation, elongation, pausing and termination) are successive clocks, each linked to enzymatic cycles (**Figures 10E, F**) (124). As in enhanceosome assembly and disassembly, each such clock (inset in **Figure 10E**) is coupled to specific multiprotein complexes. Application of this framework to architectural transcription factors would in principle require dissection of their respective enhanceosome components and associated enzymatic activities. The class of enhanceosomes whose overall activity correlates with the lifetime of a single strategic DNA bend, as envisioned for binding of SRY to the testis-specific enhancers of *SOX9* (5, 6, 76), may broadly be illustrated by a “Humpty Dumpty” model whose fall (disassembly) limits mean transcriptional initiation (**Figure 10G**) (125). The lifetime of an SRY-directed enhanceosome presumably also depends on “latching” of the multiprotein-DNA complex by partner proteins [by analogy to the SOX-POU “partner code” (148)] and not solely by the biophysical properties of the HMG box itself.

## Concluding remarks

The growing database of Swyer mutations (61) promises to provide molecular tools to dissect molecular determinants of kinetic stability in the SRY/SOX family of HMG boxes. These tools may in turn define the contribution of such structural elements to tissue-specific enhanceosome assembly and gene regulation. Rare inherited mutations in SRY, compatible with either male or female somatic phenotypes, are of particular interest as examples of a variant genetic switch poised at the edge of developmental ambiguity.

At the 62^nd^ Annual Landau Meeting of Nobel Laureates, D.R. Herschbach shared the parable of “sex and the single methyl group” in relation to Swyer mutations in SRY (149).^4^. The present study and its companion article in this issue (14) highlight this parable: our findings demonstrate the importance of a single atom, an oxygen atom in an invariant Tyr. SRY box substitution Y72F, lacking this atom and hence the anchor of a bridging water network, positions the variant testis-determining factor at the borderline of genetic function. MD simulations suggest that a Y72-anchored water molecule is long-lived on the nanosecond time scale and bridges the protein-DNA interface. MD simulations further suggest that this Y72-bridged water molecule dampens conformational fluctuations at the tail-DNA interface and augments the free-energy barrier to its detachment as a rate-limiting step in protein-DNA dissociation. These biophysical perturbations are associated with partial loss of gene-regulatory activity in a cellular model of testis determination: SRY-directed transcriptional activation of principal target gene *SOX9* (150). The proposed model of water-mediated stabilization of the SRY tail is likely to generalize to a metazoan superfamily of architectural transcription factors. In the future, this model can be tested through comparative studies of wild-type and variant box-DNA complexes by high-resolution X-ray crystallography and isotope-directed NMR studies of long-lived water molecules.

## Data availability statement

The original contributions presented in the study are included in the article/**Supplementary Material**. Further inquiries can be directed to the corresponding authors.

## Author contributions

JR co-designed, performed and interpreted the biochemical and kinetic studies of the SRY domains with purified proteins. JR, DC and MW designed and interpreted the MD studies. Y-SC and MW designed and interpreted the cell-based studies of SOX18-SRY chimeric constructs. DC, RR, YY and MW designed and interpreted the NMR studies. JR and MG undertook a survey of homologous SOX-DNA co-crystal structures. EH designed, performed and interpreted the time-resolved FRET studies of DNA bending. MW conceived the project, integrated the results, provided structural interpretations, and oversaw preparation of the manuscript. MW is the guarantor of this work and, as such, had full access to all the data in the study and takes responsibility for the integrity of the data and the accuracy of the data analysis. All authors contributed to the article and approved the submitted version.

## Funding

This work was supported in part by the INCITE Scholars Program of the Lilly Foundation and the Distinguished Professors Fund of Indiana University (MW).

## Acknowledgments

We thank Drs. P.K. Donahoe (Massachusetts General Hospital and Harvard Medicine School, Boston MA), P. Koopman (University of Queensland, Brisbane AU), R. Lovell-Badge (Francis Crick Institute, London UK), E. Pelosi (University of Queensland, Brisbane AU), K. McElreavey (Pasteur Institute, Paris, FR), N.B. Phillips (Case Western Reserve University [CWRU], Cleveland, OH), D. Schlessinger (National Institute of Aging at the U.S. National Institutes of Health, Baltimore, MD) and D. Wilhelm (University of Melbourne, AU) for helpful discussion; Prof. D.R. Hershbach for sharing his stimulating essays on chemistry and education; Dr. P.K. Donahoe, C.M. Haqq and T.R. Clarke for gift of CH34 cells; and past undergraduate/post-baccalaureate students at CWRU (M. Kesavan, P. Rangan, V. Bhatnagar, D. Leishman, B. O’Rourke, and P. Sequeira) and IU (A. Brabender and T. Tretter) for participation in early stages of this work. EH and MW. thank D. Amir for time-resolved FRET measurements and distance-distribution analysis. MW thanks Prof. M. Karplus and members of the CHARMM community for encouragement. He also acknowledges many stimulating interactions with colleagues at the International SOX Conference series and Vertebrate Sex Determination Conference series. The senior author dedicates this study to the memory of the late Prof. E.M. Purcell for his inspiring vision of “widely applied physics” in biology.

## Conflict of interest

The authors declare that the research was conducted in the absence of any commercial or financial relationships that could be construed as a potential conflict of interest.

## Publisher’s note

All claims expressed in this article are solely those of the authors and do not necessarily represent those of their affiliated organizations, or those of the publisher, the editors and the reviewers. Any product that may be evaluated in this article, or claim that may be made by its manufacturer, is not guaranteed or endorsed by the publisher.

## Footnotes

1 Genes are designated in italics, and proteins without italics. Primate genes and proteins are capitalized in full (e.g., genes SRY and SOX9; proteins SRY and SOX9), and other mammalian proteins and genes only initial capitalization (e.g., genes Sry and Sox9; proteins Sry and Sox9). Amino acids are abbreviated by standard one or three letter codes (e.g., Y72 or Tyr72).

2 Although not expressed in the embryonic gonadal ridge or fetal Sertoli cells, the autosomal gene Sox18 regulates angiogenesis, lymphangiogenesis and hair f ollicle differentiation (25). In murine embryogenesis Sox18 is expressed at 9 days post coitum in endothelial cells to induce lymphatic endothelial cell fate. Human SOX18 is of translationa interest in relation to tumor-associated neo-lymphangiogenesis as a mechanism of metastasis.

3 The solution structure of the SRY box-DNA complex (3) was not employed in the MD simulations due to differences in DNA conformation relative to SOX-DNA co-crystal structures.

4 To convey the beauty of chemistry, Prof. Hershbach highlighted the critical importance of the δ-methyl group of SRY box residue I13, which inserts between AT base pairs (5’ATTGTT-3’ and complement (115)) as a cantilever to disrupt base stacking at a site of sharp DNA bending (21, 112). “As a graduate student, more than 50 years ago,” he shared, “I heard Isidor Rabi [Nobel Laureate in Physics 1944 for observation of NMR in molecular beams] make a fervent appeal to cultivate common ground, shared by science and liberal arts. Ever since, in my own teaching, I’ve strived to do what he urged … Scientists could contribute to humanities courses what I call ‘parables’; stories that convey both a few basic facts and, more important, perspectives and modes of scientific thinking that transcend any particulars... Here is a sample parable: ‘Sex and the Single Methyl Group.’ For its first few weeks in the womb, the sex of a human fetus cannot be distinguished, whether it has XX or XY chromosomes. During about the seventh week, a genetically programmed switch kicks in. For a normal XY fetus, some already developing female structures then fade away and male structures begin to form. In rare cases (about one per 50,000 to 100,000), however, the switch is defective; the XY fetus continues on to become a woman, with external anatomy correct in all respects, although she is infertile.” By analogy, the present study extends this parable to “sex and the single water molecule.” Prof. Hershbach’s essay is available at https://faculty.chemistry.harvard.edu/files/dudleyherschbach/files/seeding.pdf?m=1363957634.
