# Supplementary material for: Tenuous transcriptional threshold of human sex determination. II. SRY exploits water-mediated clamp at the edge of ambiguity
Source: Front Endocrinol (Lausanne). 2022 Dec 2;13:1029177. doi: 10.3389/fendo.2022.1029177 (PMC9771472; doi:10.3389/fendo.2022.1029177)
Supplement: Supplementary file 1 [file DataSheet_1.pdf]

Supplemental Information

for

Tenuous Transcriptional Threshold of Human Sex Determination. II.

SRY Exploits Water-Mediated Clamp at Edge of Ambiguity

Joseph D. Racca, Deepak, Chatterjee, Yen-Shan Chen, Ratan Rai, Yanwu Yang, Millie M. Georgiadis, Elisha Haas, & Michael A. Weiss

Purpose of Supplement

This Supplement, containing 22 figures and 12 tables, describes the use of NMR and molecular dynamic protocols to investigate the role of water at the interface of protein-nucleic acid complexes. Seminal publications are outlined as a primer to highlight the application of NMR techniques to the study of water molecules bound at protein surfaces and protein-DNA interfaces.

The Supplemental Figures pertain to biological background and sequence conservation, additional molecular structures, compilation of permutation-gel electrophoresis data, canonical B-DNA and A-DNA CD spectra, summary key prior studies of the SRY tail and Swyer mutations in SRY, additional biophysical studies of the Y72F box-DNA complex (NMR and FRET), details regarding MD simulations, summary of related clinical mutations in SRY, structural analysis of SOX-DNA co-crystal structures, and electrostatic surfaces at the box-DNA interface.

Supplemental Tables provide respective summaries of SOX-DNA co-crystal structures, the present set of molecular dynamics (MD) simulations, the various biophysical analyses (fluorescence-based and NMR), and clinical mutations in SRY and homologous SOX domains.

Table of Contents

Purpose of Supplement 1
Table of Contents 1
Residue Numbering 3
NMR Detection of Bound Water 3
Figure S1 5
Figure S2 6
Figure S3 7

Figure S4 8
Figure S5 9
Figure S6 10
Figure S7 11
Figure S8 12
Figure S9 13
Figure S10 14
Figure S11 15
Figure S12 16
Figure S13 17
Figure S14 18
Figure S15 19
Figure S16 20
Figure S17 21
Figure S18 22
Figure S19 23
Figure S20 24
Figure S21 25
Figure S22 26
Table S1 27
Table S2 28
Table S3 29
Table S4 30
Table S5 31
Table S6 32
Table S7 33
Table S8 34
Table S9 35
Table S10 36
Table S11 37
Table S12 38
Footnote 40
Supplemental References 41

Residue Numbering. Clinical mutations in human SRY are ordinarily given in relation to residue numbers in the full-length protein. For clarity, consensus positions in the HMG box are also given; e.g., Y127 in full-length SRY is residue 72 in an HMG box consensus (1).

NMR detection of bound water

In favorable cases NMR spectroscopy can provide evidence for bound water molecules at specific sites in proteins or at protein-ligand interfaces. The behavior of a water molecule can be investigated by looking at NMR properties reflecting in exchange processes at the micro-to millisecond timescale as in principle probed by four different nuclei: these are the three isotopes of hydrogen: proton (^{1}H), deuterium (^{2}H), tritium (^{3}H), and oxygen ^{17}O. Of these, the most widely and extensively studied nucleus is ^{1}H; only limited applications of ^{2}H and ^{17}O NMR studies of water have been described. Hydration waters that exchange with the bulk water on a time scale of seconds was deduced from ^{18}O tracer experiment (2). The dispersion of the water ^{1}H longitudinal relaxation rate address micro-to-millisecond residence time (3).

NMR is sensitive to exchange of water molecules in and out of a specific hydration site and can within certain ranges be used for quantitative measurements of exchange lifetimes (4). NMR experiments for the detection of intermolecular NOEs between a proton in a protein (or within DNA) with water include: (a) Water suppression using pair of spin-lock pulses, (b) Watergate and Diffusion filter; (c) Selective water excitation and Dipolar field effects also employed for NOEs between water and biomacromolecules (5). The sign of the NOE cross relaxation rate changes for water residence times in the range 0.1-1ns. Thus, NOE measurements provide a tool to distinguish "slow" and "fast" water molecules on this time scale.

In the original studies of the homeodomain-DNA complex by Wuthrich and colleagues (4, 6, 7), experimental evidence for interfacial water molecules was indirect: cavities and crevices at or near the protein-DNA recognition surfaces. Interpretation of these gaps was enabled by MD simulations. This example motivated efforts to develop and apply direct NMR methods to detect bridging water molecules. Clore *et al* subsequently reported that NOEs between protein protons and surface hydration water can be quenched when the effective correlation time for positional rearrangement of the water protons (relative to the protein surface)—determined either by chemical exchange or by independent rotational motions of the water molecules—is much shorter than the rotational correlation time of the protein (8).

NOEs and ROEs between bound water and protein protons attached to ^{13}C or ^{15}N can be observed by recording ^{12}C-filtered two-dimensional (2D) H_2O-ROE/NOE-^{1}H-^{13}C or H_2O-ROE/NOE- ^{1}H-^{15}N heteronuclear single quantum coherence (HSQC) spectra (9). Molecular dynamics simulations were found to be critical to the interpretation of NOE data in a zinc finger-DNA complex (10). Only 6 protons failed to show intermolecular NOEs to solvent showed nearby long-resident water molecules in the MD simulations.

The effect of internal hydration on protein structure and stability were investigated by Brunne *et al.* (6). The single interior hydration water molecule located in the loop of BPTI replaced by the serine hydroxyl group in the mutant BPTI (variant G36S). The slightly reduced stability of mutant can be accounted by the loss of a hydrogen bond due to the fact that a hydroxyl moiety can donate

only one hydrogen atom to potential acceptor atoms, whereas a water molecule can donate two hydrogen atoms. MD simulations predicted a residence time of the water molecule within BPTI in the range 19-200 picoseconds (ps), with no correlation between solvent-accessible residue and their location (11). Thus, interaction between solute-water, water-water and entropic effect is more favorable than charged or non-polar solute atom. Interior hydration water molecules exchange with the bulk water on a millisecond time scale or faster, specifically between 200 μs to 1 ns for BPTI as calculated by NMR (4). MD calculation of Antennapedia homeodomain-DNA complex suggest residence time for interior waters are on the nanosecond time scale (~600 ps) which is lower end of the range determined by NMR (100 times longer than the lifetime of a surface water molecule (12)).

Pertinent to studies of mutations at protein-DNA interfaces, the NMR-derived solution structure of a variant complex (a mutant Antennapedia homeodomain-DNA complex with Cys39 replaced by Ser) provided a foundation for MD simulations in a water bath (see below), which suggested that the mutation affects the pattern of bound water molecules at the variant interface (12).

In addition to isolated proteins and the protein-DNA systems highlighted in this study, applications have also been reported to water channels (13), water-filled silica nanopores (14), and mineral-water interface (15). The changes in water-fiber interactions, for example, could be probed by various NMR relaxation parameters, double-quantum filtered (DQF), and 1D and 2D translational-diffusion experiments (16). A restricted diffusion and anisotropy of water self-diffusion could be studied by one- and two-dimensional pulse-field-gradient NMR (17).

MD simulation (500 ps) of human SRY-DNA predict only one water mediated hydrogen bond between SRY and DNA; *i.e.*, Asn10-C4 and T14 along with other 5 salt bridge (18). Thus, it is hydrophobic interaction which facilitate protein-DNA binding. In the present case, we can see more than one water mediated hydrogen bond between SRY-DNA. The possible reason for this difference is duration of MD calculation.

Future direction of the human SRY-DNA complex will be to explore the idea of detecting Tyr72 bound water. Selective ^{13}C-labeling of Tyr72 might be useful to observe ^{13}C-edited NOE's to water held at the interface. In contrast, ^{13}C-labeled Tyr74 will lack such NOE. In a variant domain-DNA complex, a ^{13}C-edited NOE for Phe72 with water might not be observable or very weak as the distance would be close to 4 Å.

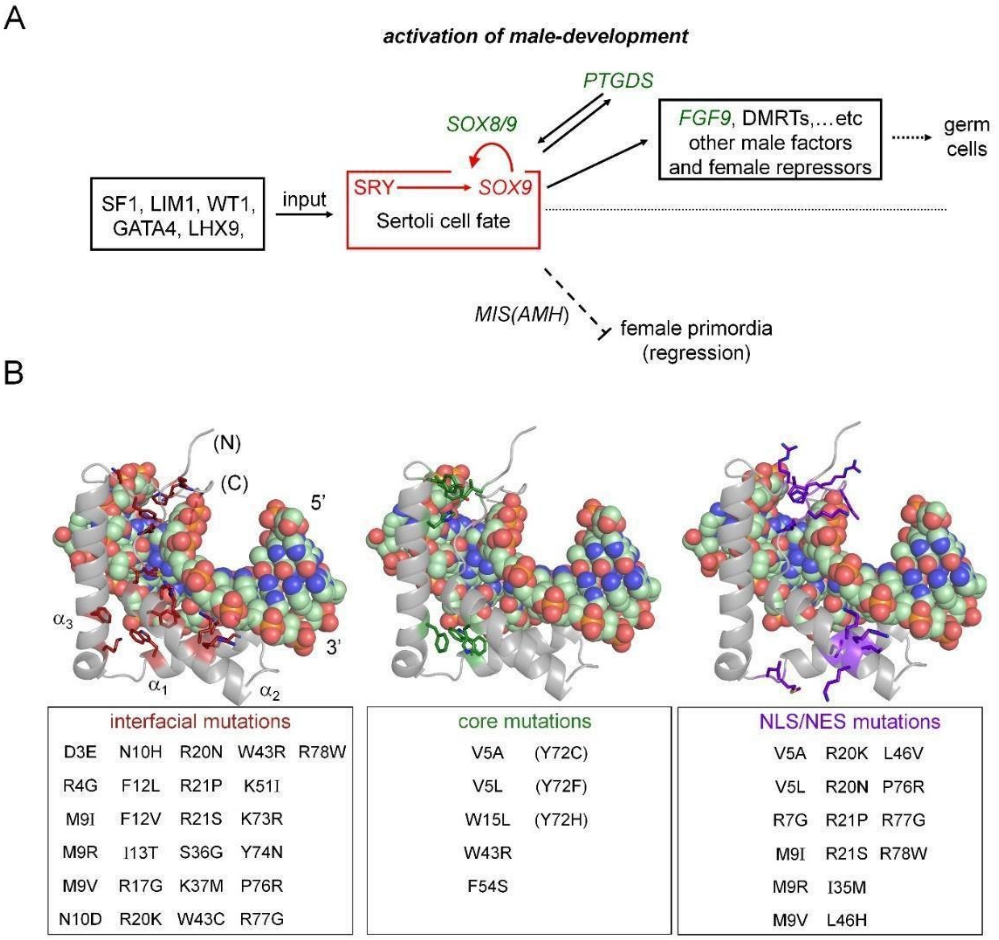

Figure S1 │ SRY-regulated gene-regulatory network (GRN), structure of SRY HMG box and clinical mutations. **(A)** SRY-dependent testis determination pathway. Red box highlights central SRY→SOX9 axis ((19, 20)). At left are genetic inputs and right are genetic outputs. To effect gonadal-ridge differentiation, SOX9 activates a male-specific GRN, in turn directing Müllerian-duct regression (dashed ⊥; Müllerian inhibiting-substance MIS; also designated anti-Müllerian hormone (AMH)) (21) while inhibiting the granulosa-cell fate ((19, 21). **(B)** SRY HMG box-DNA complex, box in gray and DNA shown as spheres. Terminal ends of the HMG domain labeled (N) and (C), helices 1-3 are designated as α and the 5'- and 3'-ends of the DNA are noted. Sites of clinical mutations shown as sticks in each category of mutations. Left, mutations that localize to the protein-DNA interface (maroon) positions are listed in the box below. Middle, mutations that localize to either the major wing hydrophobic core or a DNA-dependent mini-core (22). Mutations in parenthesis at position Tyr 72 are the site of investigation in this and our companion paper (23). Right, mutations that affect proper nuclear localization (24, 25) or nucleo-cytoplasmic shuttling of human SRY (26). Human SRY HMG box-DNA structure PDB entry 1J46 (1).

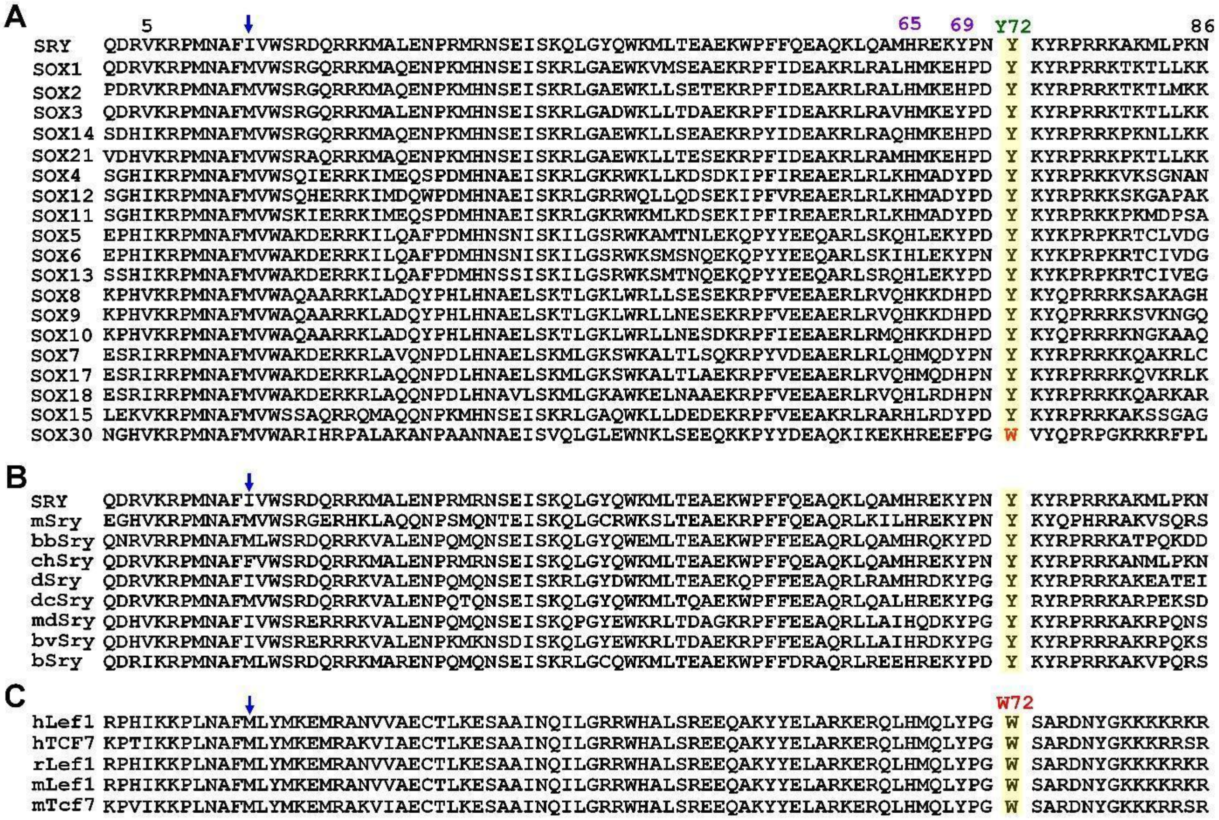

Figure S2 │Sequence alignment of the human SRY HMG domain and related transcription HMG domains. **(A)** Human SRY at the top is aligned with HMG domains from human SOX factors. Position 5 is noted (consensus HMG numbering), blue arrow indicates the site of side chain "cantilever" responsible for partial intercalation into the DNA. Aromatic residues of the hydrophobic mini-core are in purple and green (consensus numbering); Tyr 72 is highlighted as the residue of interest In this study. **(B)** Conservation of Y72 is shown among various mammalian Sry HMG domains (m=mouse, bb=brown bear, ch=chimpanzee, d=dolphin, md=mule deer, bv=bovine, b=bat). **(C)** Related HMG domains from Lef-1 (lymphoid enhancer factor-1) and TCF (T-cell factor). Homologous consensus position 72 is highlighted and conserved in this family as tryptophan. (Codes: h=human, m=mouse, r=rat,)

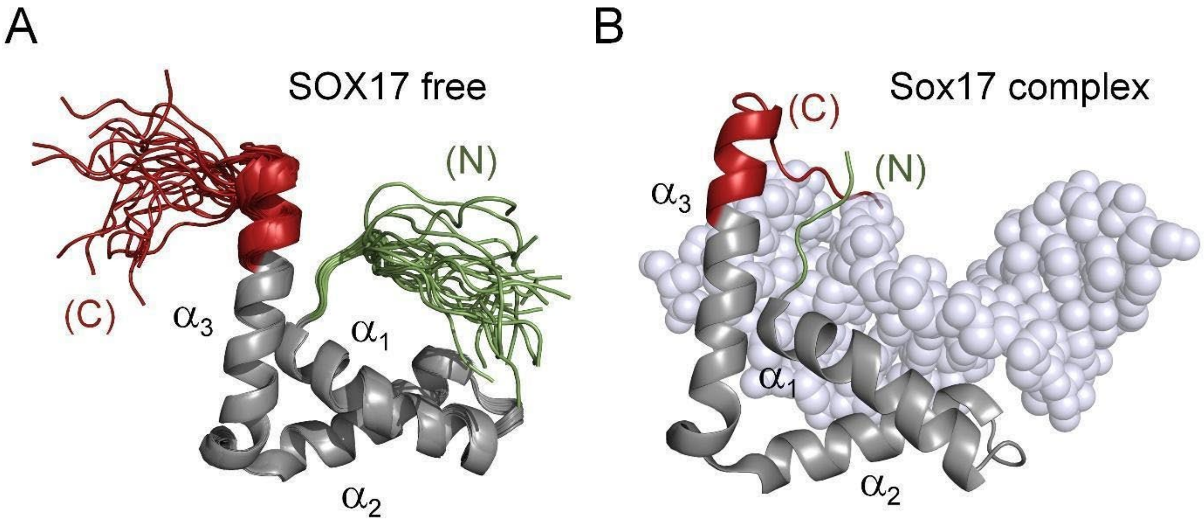

Figure S3 │Conformational differences in free and bound HMG boxes. **(A)** NMR structure of the human SOX17 HMG domain in the absence of DNA, helices are labeled ($\alpha_{1\text{-}3}$). The dynamic N- and C-termini are labeled in green (N) and dark red (C). These regions represent the dynamic minor wing. The well-organized major wing is in grey. (PBD 2YUL) **(B)** Upon DNA binding rearrangement of minor wing occurs and the termini become well structured, participating in the formation of the bent DNA-protein complex. (PDB entry 3F27 (27))

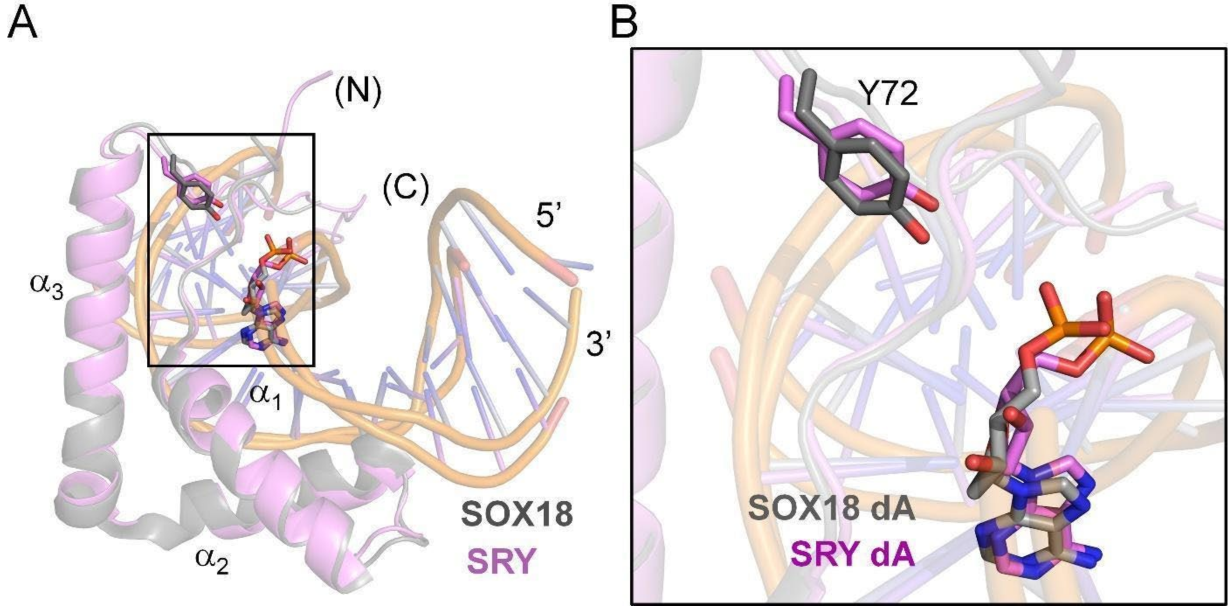

Figure S4 │Alignment of the Sox18 Box-DNA and human SRY HMG box complexes. **(A)** Overview of aligned HMG-DNA complexes; Sox18 is in grey and human SRY HMG is in light purple. The α-helices, termini and the ends of the DNA are labeled. Boxed, side chain of Tyr 72 (consensus numbering) is shown for each domain in the respective color. The DNA base and phosphate backbone at the interface is shown as sticks. **(B)** Expanded view of the boxed region. Positioning of the Y72 side chain in Sox18 and human SRY HMG are similar whereas position of the phosphate in the DNA backbone is different. DNA bases are complementary colored with the protein. Images from PBD 1J46 for human SRY-DNA complex (NMR; ref 1) and PDB entry 4Y60 for Sox18-DNA complex (crystallography; ref 28).

```
                                                                              Tyr of "water-clamp"
     SRY  VKRPMNAFIVWSRDQRRKMALENPRMRNSEISKQLGYQWKMLTEAEKWPFFQEAQKLQAMHREKYPN Y KY
    rSry  VKRPMNAFMVWSRGERRKLAQQNPSMQNSEISKHLGYQWKSLTEAEKRPFFQEAQRLKTLHREKYPN Y KY
    dSry  VRRPMNAFLVWSRDQRRKMALENPQMQNSEISKQLGYQWKMLTEAEKWPFFEEAQRLQAMHREKYPD Y KY
   hSOX4  IKRPMNAFMVWSQIERRKIMEQSPDMHNAEISKRLGKRWKLLKDSDKIPFIREAERLRLKHMADYPD Y KY
  hSOX10  VKRPMNAFMVWAQAARRKLADQYPHLHNAELSKTLGKLWRLLNESDKRPFIEEAERLRMQHKKDHPD Y KY
 dmSox15  IRRPMNAFMVWAKIERKKLADENPDLHNADLSKMLGKKWRSLTPQDRRPYVEEAERLRVIHMTEHPN Y KY
  dmSoxN  VKRPMNAFMVWSRGQRRKMASDNPKMHNSEISKRLGAQWKDLSESEKRPFIDEAKRLRAVHMKEHPD Y KY
  drSox9  VKRPMNAFMVWAQAARRKLADQYPHLHNAELSKTLGKLWRLLNEVEKRPFVEEAERLRVQHKKDHPD Y KY
  drSox2  IKRPMNAFMVWSRGQRRKMAQENPKMHNSEISKRLGAEWKLLSESEKRPFIDEAKRLRALHMKEHPD Y KY
   xSox2  VKRPMNAFMVWAQAARRKLADQYPHLHNAELSKTLGKLWRLLNEGEKRPFVEEAERLRIQHKKDHPD Y KY
   xSox9  VKRPMNAFMVWSRGQRRKMAQENPKMHNSEISKRLGADWKLLSDSDKRPFIDEAKRLRAVHMKDYPD Y KY
  xSox18  IRRPMNAFMVWAKDERKRLAQQNPDLHNAVLSKMLGQSWKNLSSAEKRPFVEEAERLRVQHLQDHPN Y KY
 ecSoxD1  IKRPMNAFMVWAKEERRKILARHPDMHNSNISKILGSKWKTMSNAEKQPYYEEQARLDKAHLEKYPD Y KY
                                                                      Y72
```

Figure S5 │Conservation of Tyr "water-clamp". Representative metazoan HMG domains highlighting conservation of Tyr at the "water-clamp" interface. (abbreviations: r, rabbit; d, dog; h, horse; dm, *drosophila melanogaster* (fruit fly); dr, *danio rerio* (zebrafish); x, *xenopus laevis* (african clawed frog); ec, purple sea urchin)

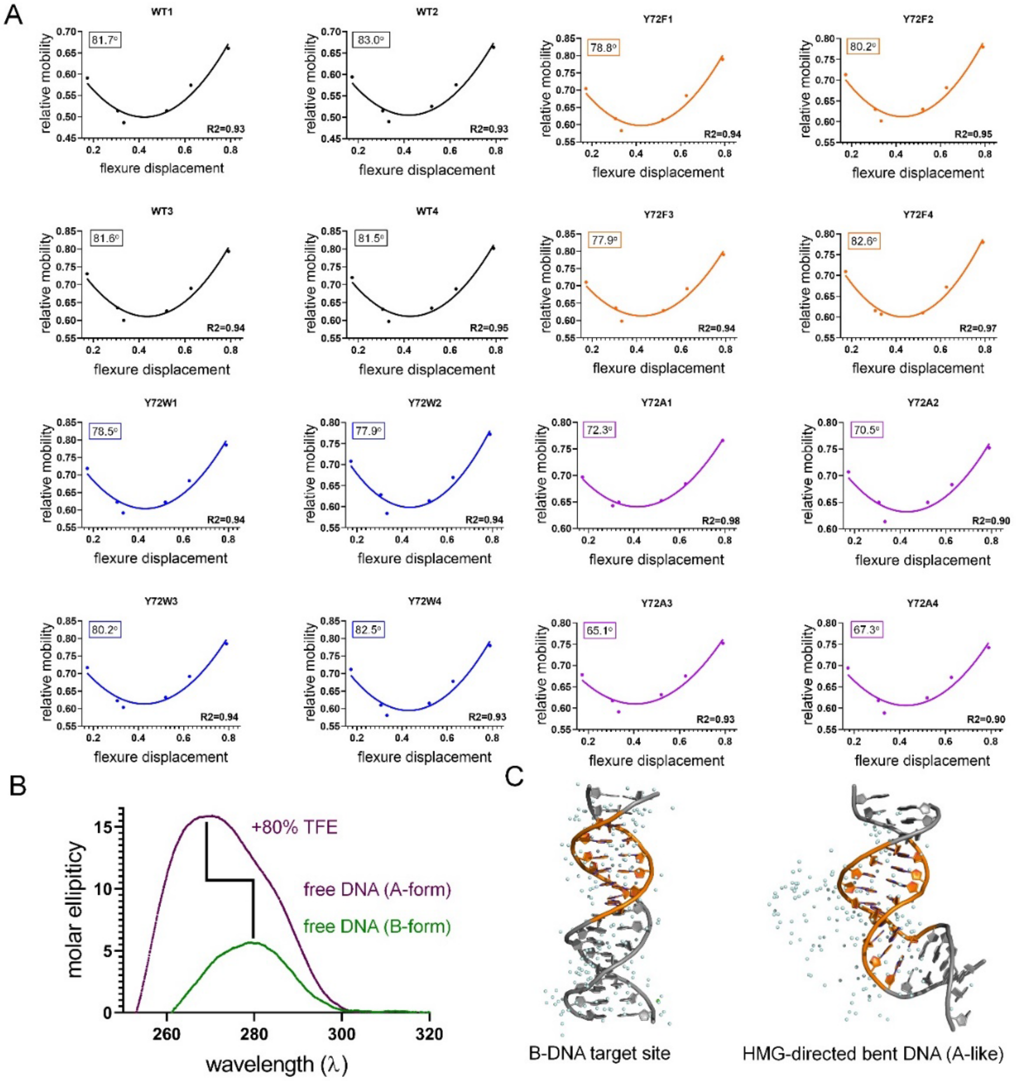

Figure S6

Figure S6 │. DNA bending plots of WT and Y72A variant SRY HMG boxes. (**A**) DNA bend angles reported in main text are derived from four replicates of permutation gel electrophoresis experiments. Plots represent measurements made of each repeat; DNA bend angle calculated from each experiment is boxed in upper left. Variants are listed, top center, but also color coded: WT (black), Y72F (orange), Y72W (blue) and Y72A (light purple). (**B**) Circular dichroism of free DNA in an aqueous buffer exhibiting typical B-DNA CD spectra (green trace), the same DNA in a buffer containing 80% trifluoroethanol to induce A-DNA conformation (purple trace). The broken line highlights the peak maxima shift in the B-to-A transition. (**C**) Structural models of a B-DNA target site (left, PDB entry 2BSE, (101)) with the SRY consensus sequence in orange. Water molecules observed in this structure are in pale cyan. Right, structural model of A-like bent DNA by the Sox18 HMG box. Crystallographic water molecules are in pale cyan (PDB entry 4Y60 (28).

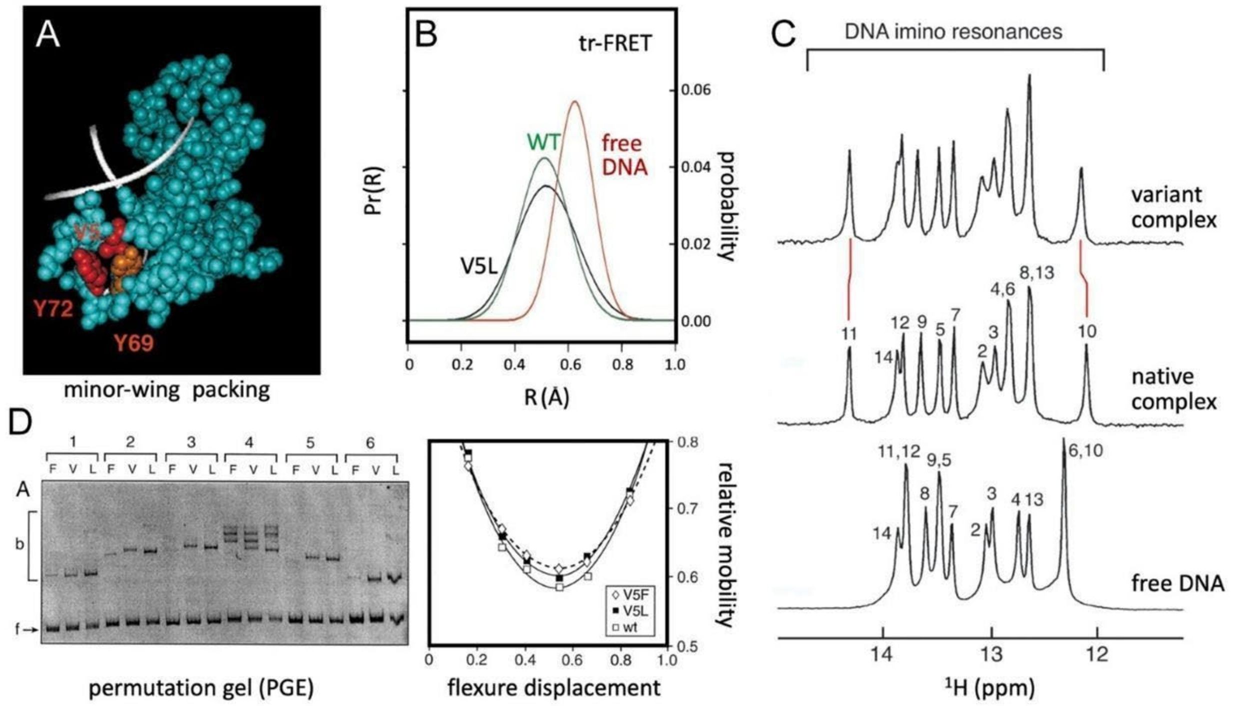

Figure S7 │Minor wing mini-core Y72-related mutation (29). **(A)** Sphere and ribbon representation of the SRY HMG-DNA complex (protein in spheres and DNA in ribbon). Residues of the DNA-dependent hydrophobic mini-core are shown as colored spheres; C-terminal residues Y69 and Y72 labeled and N-terminal V5 all in red spheres. **(B)** time resolved FRET of the mini-core related mutant V5L (black) compared to WT (green) and free double-labeled fluorescent DNA (red). **(C)** 1D spectra of the free DNA (bottom) and spectra of the DNA in specific complex with WT (middle) and the V5L variant (top). Red lines indicate chemical shift differences between the WT and variant complexes. **(D)** Permutation gel electrophoresis of the WT and two different variant domains with subsitions at position Val5. Sets are indicated at the top of the gel as F (V5F), V (WT), and L (V5L). Relative distance of migration of the specific complex (compared to free DNA) is plotted versus the flexure displacement.

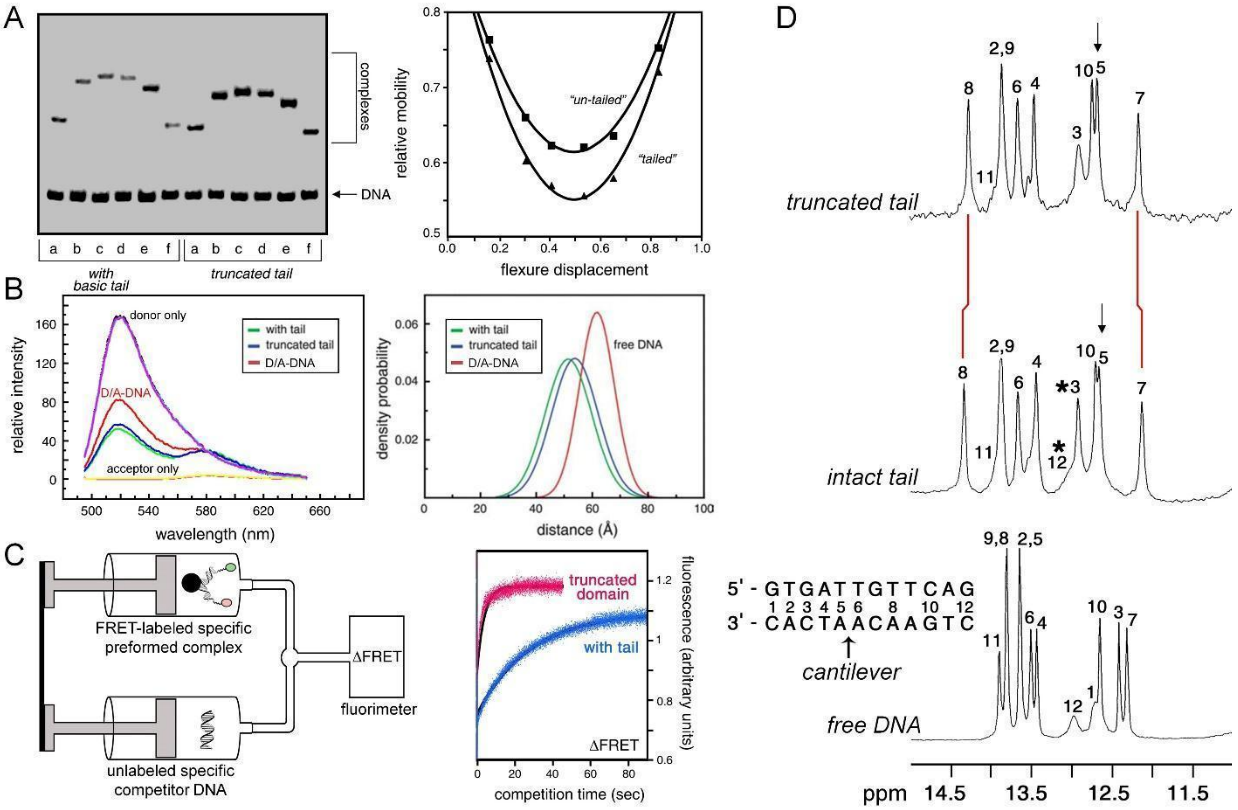

Figure S8 │Kinetically clamped tail of the SRY HMG-DNA complex (30). **(A)** Permutation gel electrophoresis of the WT (basic tail) and C-terminal truncated (truncated tail) HMG domains with plots to the right of the gel. "Tailed" indicated full length HMG box (WT) and "un-tailed" indicates a truncation at the C-terminal end. **(B)** steady state FRET and time resolved FRET comparing the WT (with tail) to the shortened HMG domain (truncated tail). Spectra collected with free DNA is in red, WT in green and truncated domain in blue. **(C)** stopped flow FRET scheme showing a specific HMG-DNA complex in one syringe and a second syringe loaded with unmodified target DNA at 20-fold molar excess. Rapid mixing of an equal volume from each syringe in a chamber, monitoring emission at 520nm, results in loss of the FRET between donor and acceptor and regain of donor mission indicating off rate. Tailed protein (WT) is in blue and the C-terminal truncated proteins in red. **(D)** 1D ^{1}H NMR of the DNA (sequence listed at bottom), free DNA is lower panel, WT complex (intact tail) is middle and truncated protein is at the top. Red lines indicate chemical shift differences between the WT and variant SRY box-DNA complexes.

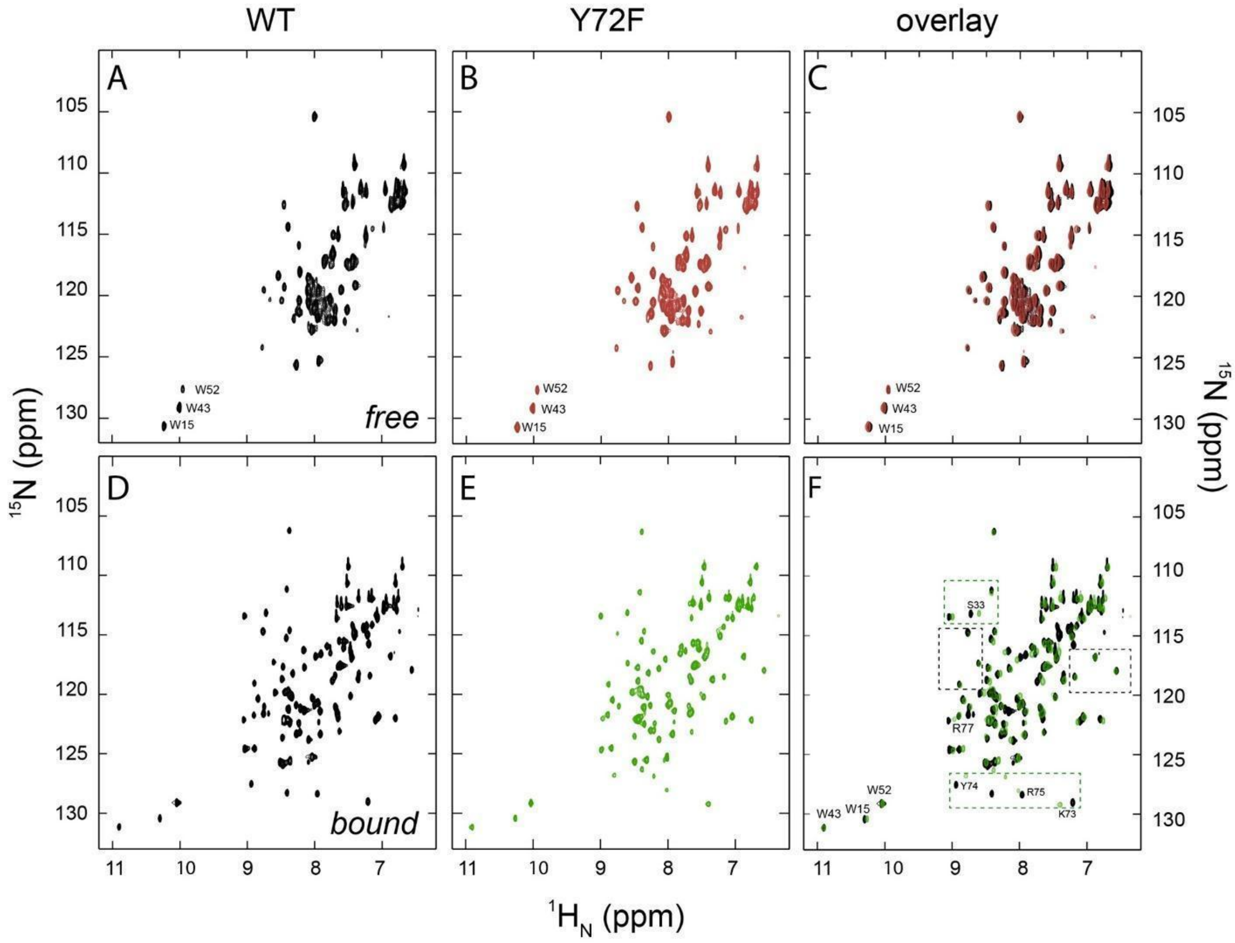

Figure S9 │2D ^{1}H- ^{15}N HSQC footprints of free SRY HMG and protein–DNA complexes. **(A)** Free WT (black) **(B)** free Y72F (maroon) **(C)** spectral overlay of free WT (black), free Y72F (maroon). **(D)** Bound WT (black) **(E)** bound Y72F(green) and **(F)** spectral overlay of bound WT (black), bound Y72F (green). In panel F, the black dotted box represents cross-peaks from both WT-SRY and Y72F that are similar in their respective complexes. Green boxes represent chemical shift changes between WT-SRY and variant cross-peaks. Presumptive assignments are as indicated. Residues Y74, R75, R77, K73 and S33 show significant chemical shift perturbation. For tryptophan, two Trp residues are in the core (W15 and W43) and one exposed (W52) are labeled. All spectra were acquired at 25 °C.

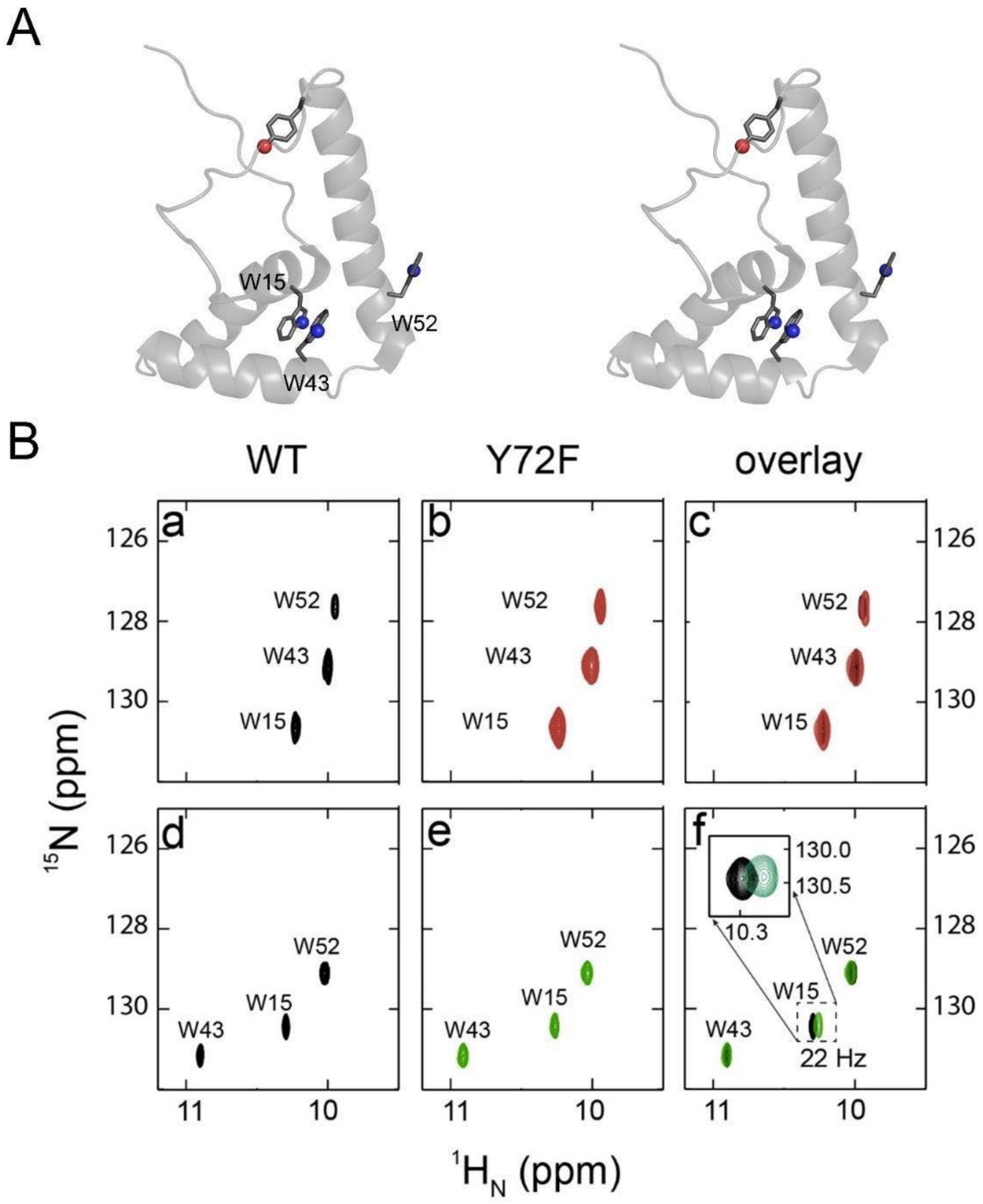

Figure S10 | SRY HMG domain structure and zoomed 2D ^{1}H- ^{15}N HSQC spectra of three cross-peaks corresponding each Trp side-chain indole. **(A)** Stereo-view of the HMG complex (DNA not shown). Side chains of the Trp residues and Y72 are shown as sticks; nitrogen of the indole rings are shown as blue spheres and hydroxyl oxygen of Y72 shown as red sphere. **(B)** Presumptive assignments of three indole (W15, W43 and W52) are as indicated. **(a)** free WT (black) **(b)** free Y72F (maroon) **(c)** spectral overlay of free WT (black), free Y72F (maroon). **(d)** bound WT (black) **(e)** free Y72F (green) **(f)** spectral overlay of bound WT (black), bound Y72F (green). Human SRY HMG box-DNA structure PDB coordinates 1J46 (1). All spectra were acquired at 25 °C.

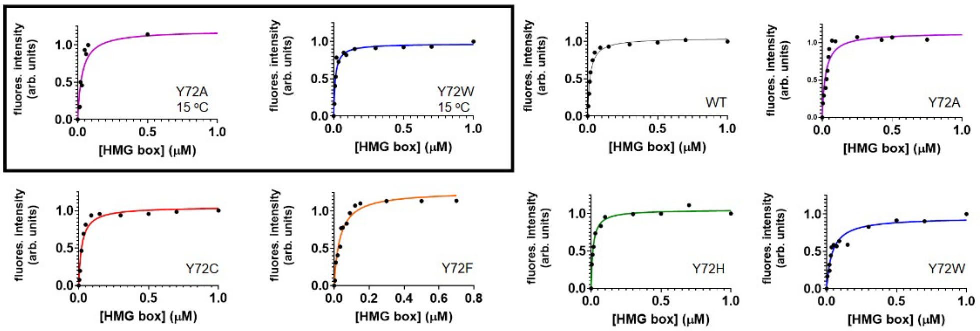

Figure S11 │ DNA binding and bending activities of the WT and variant HMG domains. Equilibrium binding studies of the WT and variant domains; boxed is Y72A and Y72W as measured at 15 ºC; K_d values of Swyer variants at 15 ºC were reported in our companion paper (23). The other plots are measured at physiological (37 ºC) temperature (values reported in **Supplemental Table S1**).

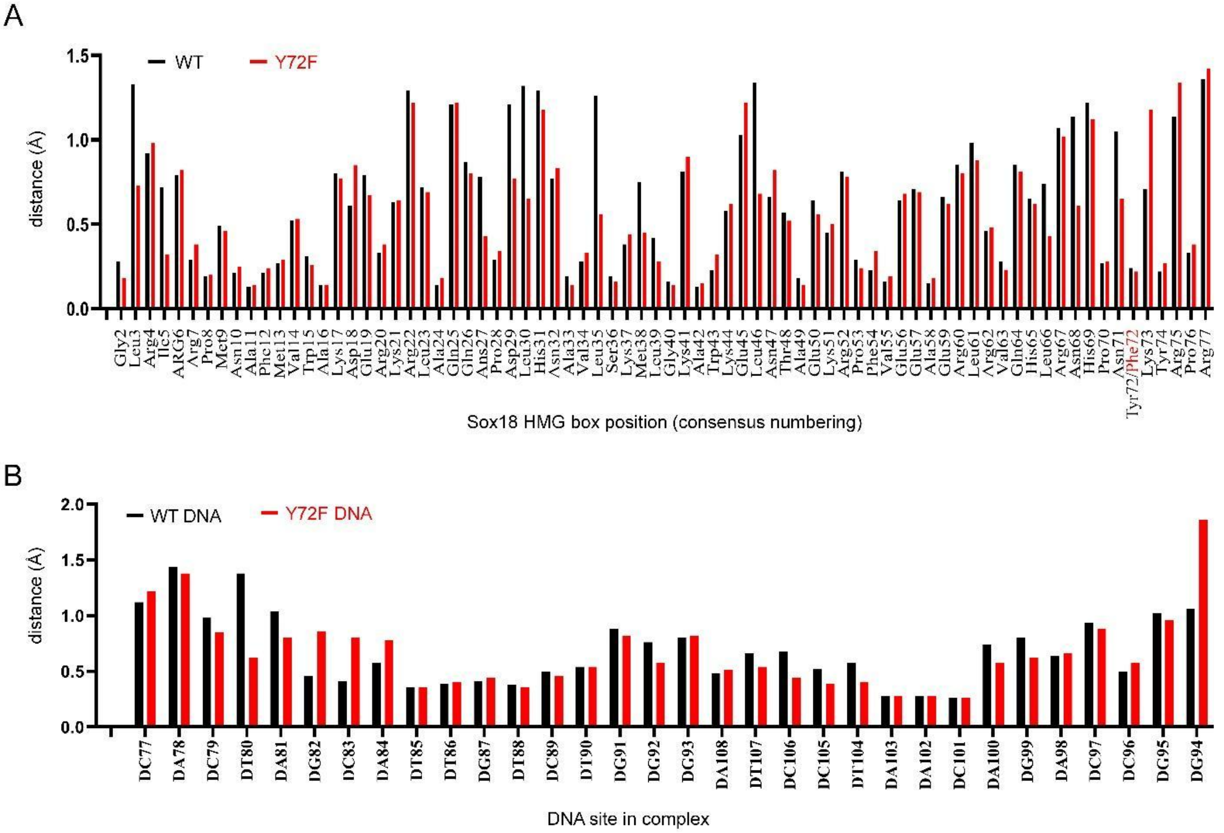

Figure S12 │ Calculated all atom RMSD for DNA and Protein with respect to the starting structure after aligned with α-carbon. (**A**) RMSD histogram for WT SOX18 (black) and variant (red). For all practical purposes, three helices showed similar RMSD, whereas C-terminal tail for variant reveal more fraying. (**B**) DNA RMSD histogram for WT complex (black) and variant (red). Binding site of 5'-ATTGT-3' remain intact for both WT and variant.

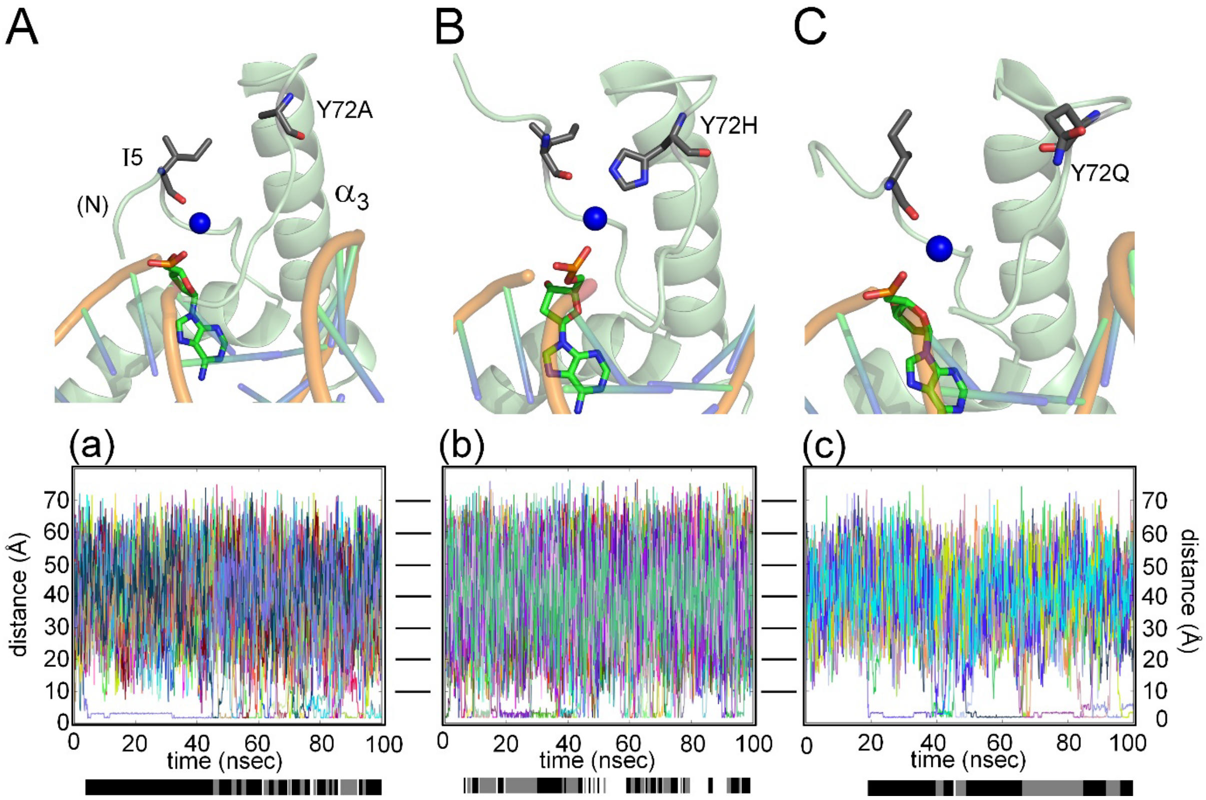

Figure S13

Figure S13 │ Sox18 Y72X variants at the protein-DNA-water interface. (**A**) Structural depiction of the Y72A variant complex at a time point during MD. The protein and DNA-shared water is shown as a blue sphere and is within hydrogen bonding parameters to a phosphate oxygen and the carbonyl of position 5 (both shown as sticks) in the N-terminal region. Alanine at position 72 is labeled. (a) The trajectory of the water molecules that occupy the protein-DNA-water site during the course of the simulation and associated Y72A-water barcode below. Similar structural depiction for Y72H (**B**) and protein-DNA-water trajectories (b). (**C**) Y72Q variant model from an MD timepoint. The Gln side chain appears a similar conformation throughout the simulation. (c) Water trajectory profile for occupancy of water molecules at the protein-DNA site and the barcode below plot.

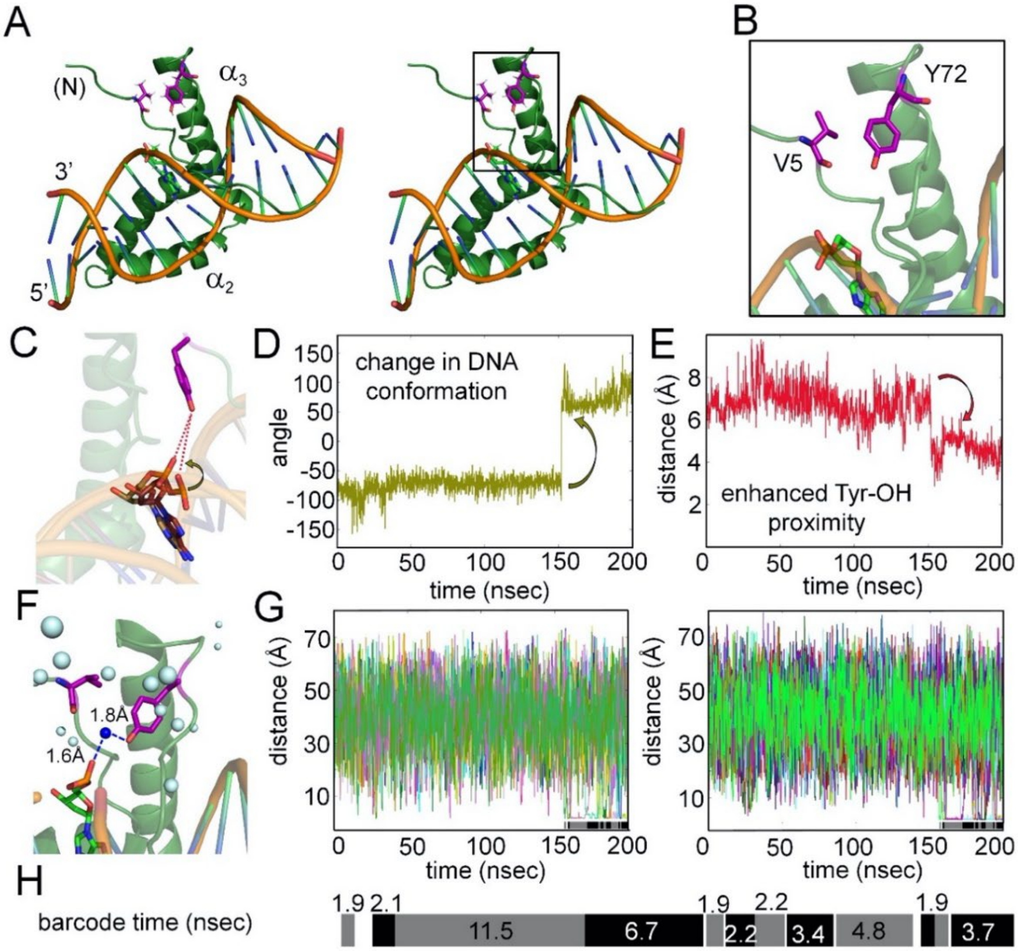

Figure S14

Figure S14 │ Molecular dynamic analysis of the human SRY HMG-DNA NMR structure (PDB entry 1J46 (1))**.** (**A**) Stereo-view of the NMR structure of human SRY HMG-DNA complex. Helices 2 and 3 are labeled, helix 1 underlies the DNA. Residues and DNA backbone phosphate involved in the "water-clamped region" and shown as sticks; Val5 and Tyr72 in purple and the oxygen atoms of the phosphate backbone in red. Boxed region is expanded. (**B**) Expanded view of the region that participates in the water-mediated clamp in Sox18 and other SOX factors. (**C**) Conformational changes in the DNA backbone throughout the course of the MD time course. Arrow indicates the changes in the DNA phosphate position to an appropriate conformation for water-bridged interactions at the protein-DNA interface. Dashed arrows indicate the distance of the oxygen atom form the *para*-OH of Tyr72 in the different conformations. (**D**) Plot of the angle of the phosphate in the DNA backbone, gold arrow highlights the conformational change in the DNA. (E) Trajectory of the para-OH group of Y72 to the oxygen atom (dash lines in (**C**)) *w.r.t.* in the DNA backbone. Red arrow indicates the shift in the DNA conformation that is able to form a water-bridged hydrogen bond with Tyr72. (F) After 160 nanosecond there is a bridging water (in dark blue) that forms hydrogen bonds between Y72 *para*-OH and an oxygen atom of the DNA backbone (bulk waters in pale cyan). (**G**) Trajectories of water molecules in the SRY-DNA MD simulation. Long-lived water molecules at the Y72-DNA interface are observed after ~160ns; "barcode" represents individual waters at this interface and their time of occupancy.

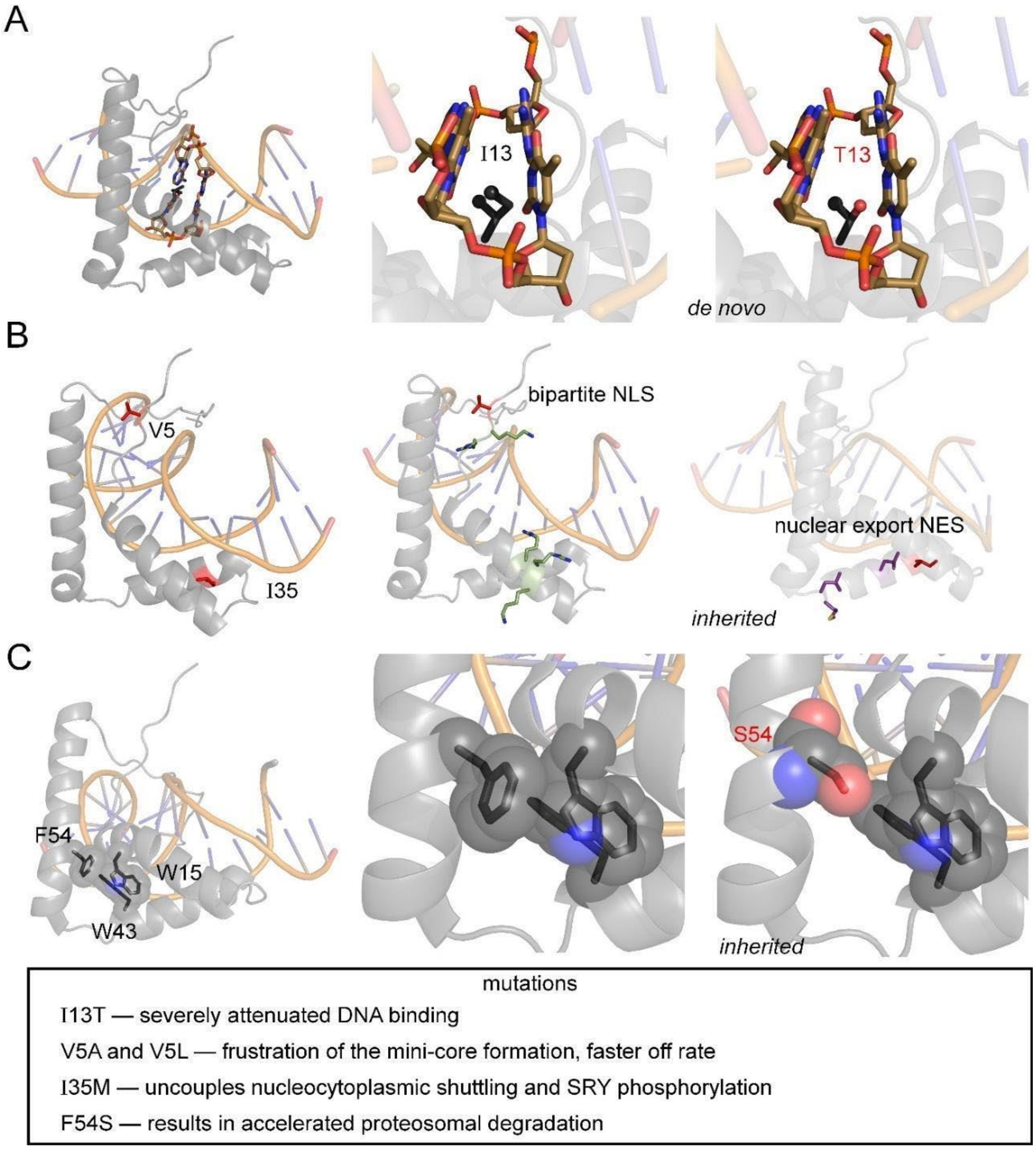

Figure S15 │ Mutations within the HMG box of human SRY HMG box. **(A)** Structure of the SRY HMG-DNA complex; protein in grey and the DNA shown as a cartoon. The intercalative side chain of I13 (consensus numbering) shown as sticks. Partial intercalation splays base stacking with splayed bases shown in sticks. Middle, expanded view of Ile13 side chain intercalation with methyl groups shown as spheres. Right, model of *de novo* mutation I13T with the methyl and hydroxyl groups shown as spheres. **(B)** Side chains of Val5 and I35 shown in red to indicate positions of mutations that affect DNA-dependent structures and nuclear localization (middle) or nucleo-cytoplasmic shuttling (right). **(C)** Aromatic residues of the major wing hydrophobic core shown as sticks outlined in spheres. Middle panels are expanded view of the hydrophobic core. Right, inherited core mutation F54S shown as sticks and outlined in spheres. Box at the bottom summarizes the mechanism of each mutation.

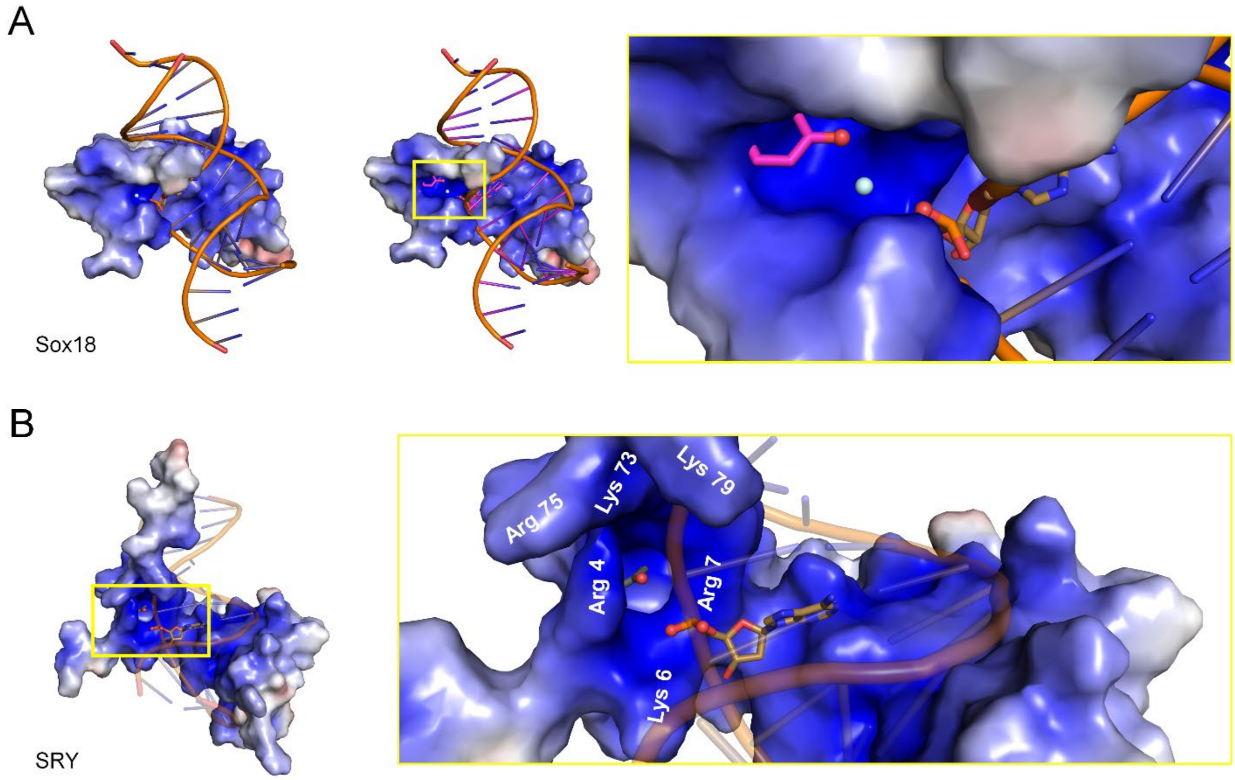

Figure S16 │ Environment of the *para*-hydroxy group of Tyr72 in the protein-DNA complex. (**A**) Electrostatic surface map representation of the Sox18 HMG box with DNA shown as a ribbon. Left panel shows the basic surface of the protein tail in Sox18 at the complex interface the crystallographic bridging water is in pale cyan. Middle panel shows the position of the side chain of Tyr72 (boxed in yellow). Right, expanded view of the protein (Tyr72)-DNA interface and bridging crystallographic water. (**B**) Electrostatic surface map of the HMG box of human SRY complex with the DNA shown as a ribbon. Left is an overview of the structure, Tyr72-related interface is boxed in yellow. Right, expanded view of the interfacial environment of the *para*-hydroxyl of Tyr72 in SRY. Neighboring residues at this site in the human SRY complex are labeled in white. PDB entries: (Sox18) 4Y60, and (SRY), 1J46.

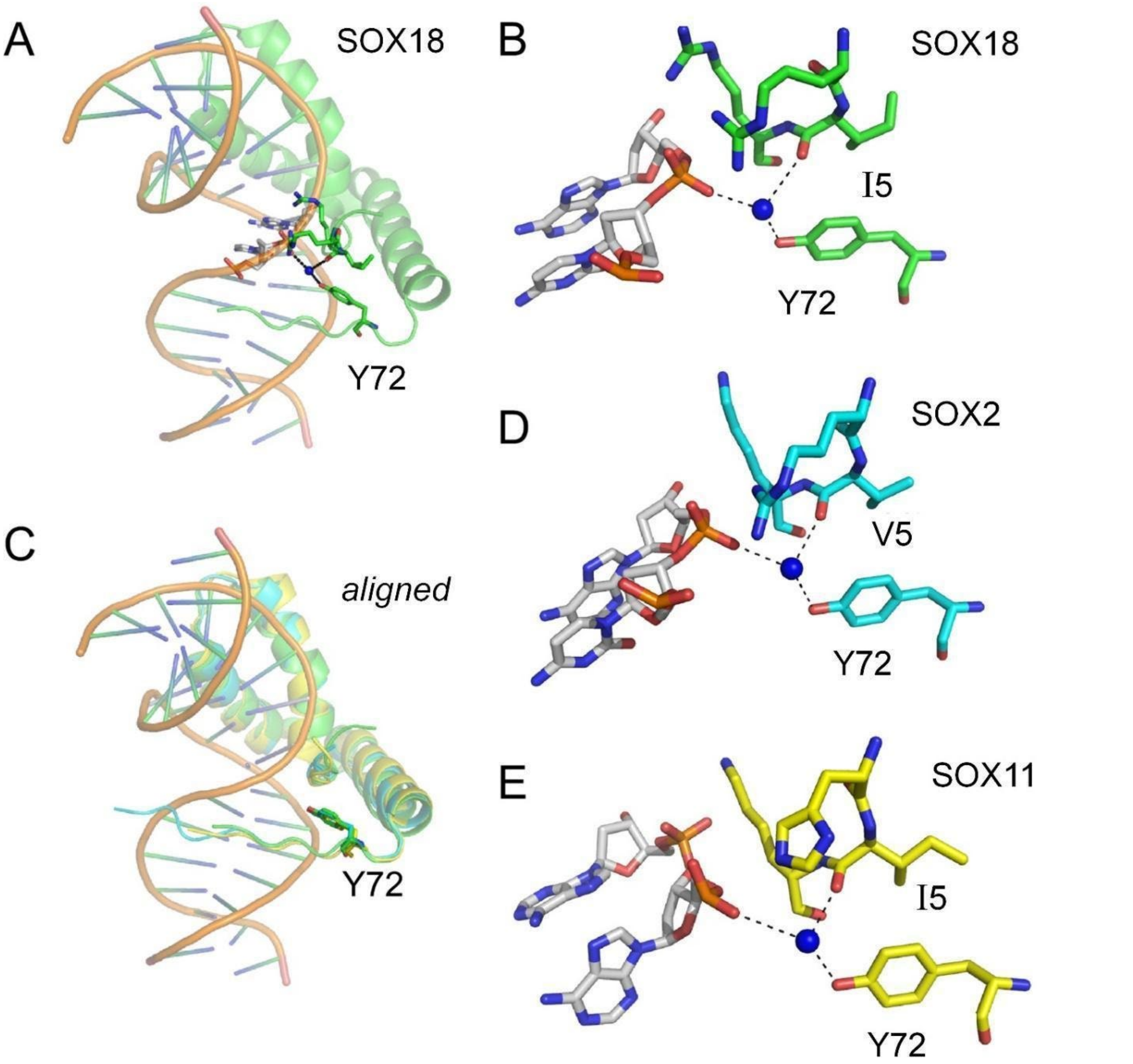

Figure S17 │ Comparison of bridging water molecule in Sox HMG box-DNA structures. **(A)** The structure of SOX18 HMG-DNA is shown in a cartoon rendering with the protein in green and DNA in sand. Tyr72 (consensus numbering) in the SOX18 structure, along with residues 2-4 including I3 are shown in a green stick rendering and two nucleotides are shown in gray (the color refers to C atoms, O are red, nitrogen blue, and P orange). The hydrogen bonds between the bridging water molecule, the protein, and the DNA are shown as black dashed lines. **(B)** A close-up of the hydrogen-bonding network involving the bridging water molecule is shown for SOX18. The water molecule is shown as a blue sphere. **(C)** HMG boxes for SOX18 (green), SOX2 (cyan), and SOX11 (yellow) are shown superimposed in cartoon renderings with the DNA from the SOX18 structure. In these structures the Y72 equivalent residues shown in a stick renderings superimpose exactly. Close-up stick renderings for SOX2 **(D)** and SOX11 (E) show that the bridging water molecule interactions are identical in each of these structures. PDB entries are: (Sox18) 4Y60, (Sox2) 1GT0, and (Sox11) 6T78 as described in references (28, 31, 32).

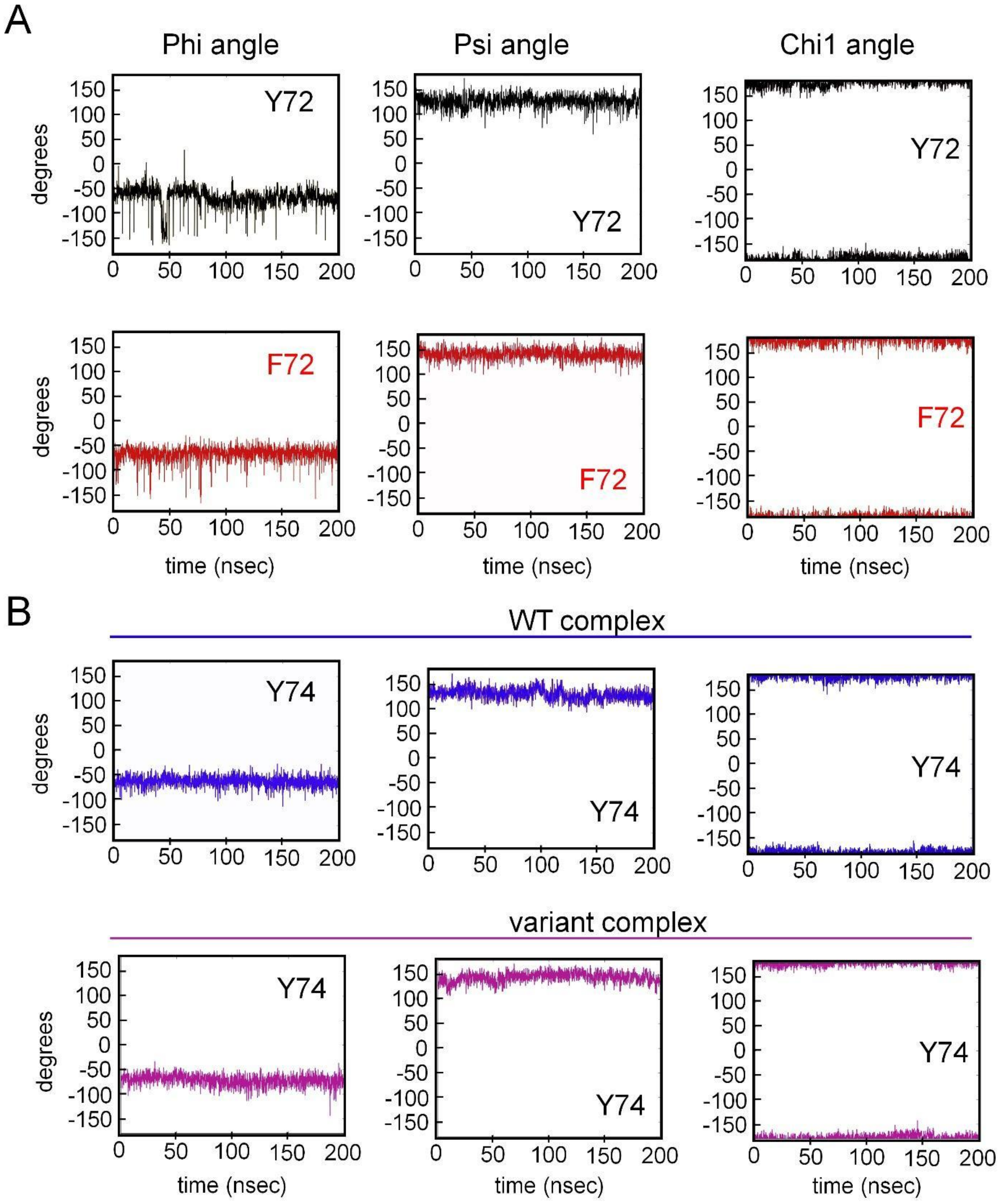

Figure S18 │ Dihedral angle Phi, Psi and Chi1 variation over 200ns MD for Sox18-DNA complex. (**A**) Dihedral angles of the Tyr and Phe at position 72 in respective complexes; WT in black and variant in red. (**B**) Dihedral angles for an anchored tail residue, Y74, in each of the complexes; WT complex (Y74 angles in blue) and for variant complex (Y74 angles in magenta).

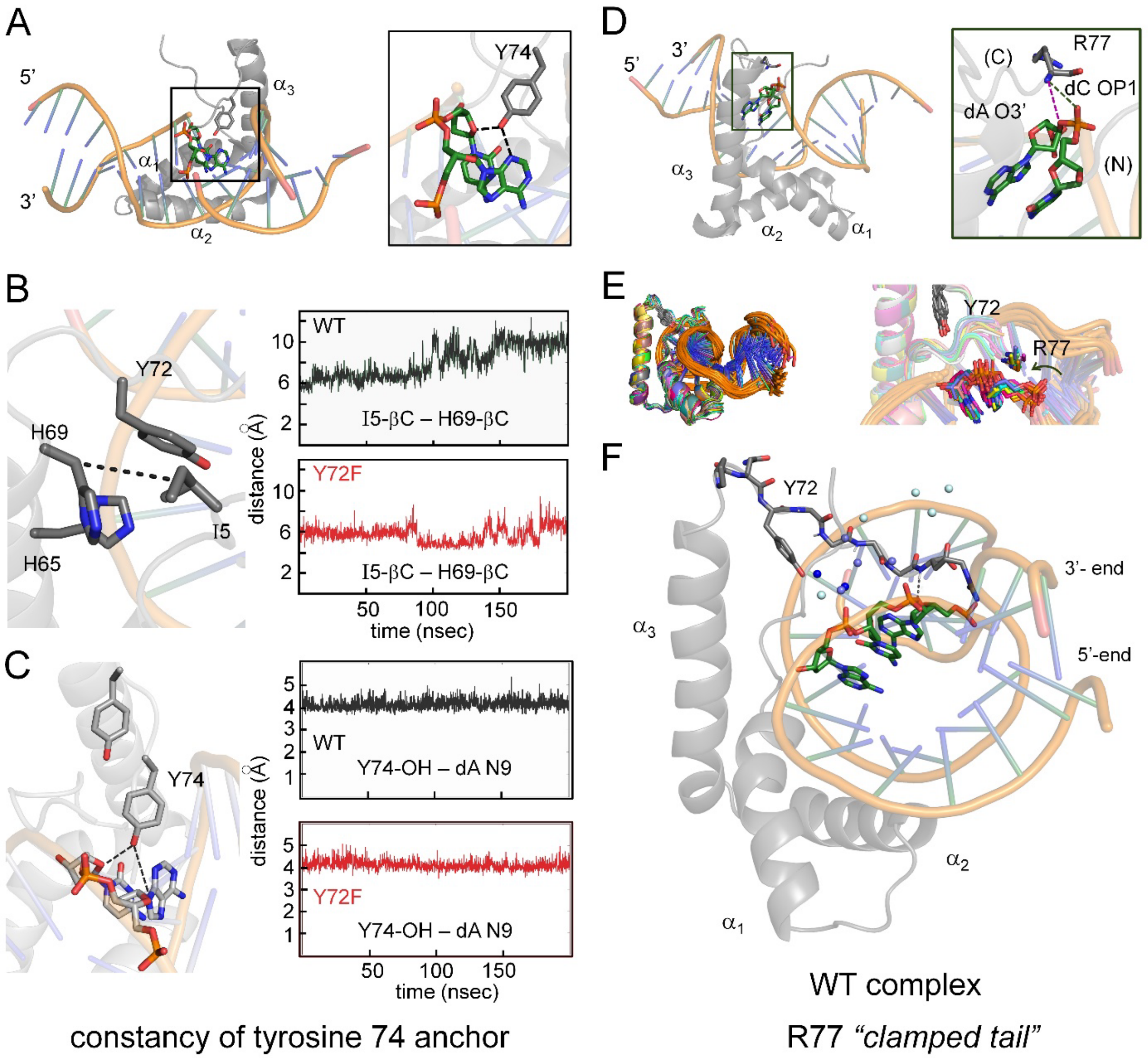

Figure S19 │ Minor-wing and tail fluctuations during MD. (**A**) Crystal structure overview of the Sox18-DNA complex with box highlighting conserved Tyr74 in the C-terminal tail region. The interaction between Tyr74 sidechain and DNA base shown is as sticks, with inset showed expansion of the same. Broken line show polar contact of hydroxyl group of Tyr74 with ribose sugar and nucleobase. (**B**) Hydrophobic mini-core of His69, His65, Tyr72 and Ile5 shown as sticks in Sox18-DNA complex. Relative movement of Ile5 Cβ and His69 Cβ in the mini-core shown for WT (black) and variant (red). WT indicates that Ile5 moves away from His69 during the simulation corresponding to the single water to two water-mediated hydrogen-bond motifs. Though, for variant, Ile5 remains close to the His69. (**C**) Trajectories of the Y74 *para*-hydroxyl to a nitrogen of the adenine nucleobase. In each case the trajectories of the WT (black) and variant (red) are similar, indicating that Y74 remains anchored during the simulation. (**D**) Structural overview of WT C-terminal Arg77 and DNA shown as sticks. Expanded view of the same shown in inset; the

broken line represents a hydrogen bond involving the amide of Arg77 with DNA OP1 (black, designating a phosphate bound oxygen atom) and O3' (purple, designating the sugar O3 atom). (**E**) An ensemble of 190-200 ns with step size of 0.25 ns of the WT-DNA complex. Arg77 amide and DNA backbone of hydrogen bonding partners were shown as sticks for the ensemble with an arrow indicating the relative position. Trajectories for Arg77 amide-OP1 (black) and O3' (purple) with same color code for hydrogen-bond angle during the course of 200 ns MD simulation. (**F**) Structure of the Sox18-DNA WT complex from MD showing a network of waters at the tail-DNA interface. The blue hue of the water molecule indicates its relative occupancy (dark being longer lived). The amide-DNA hydrogen bond of the upper strand of R77 is indicated by a dashed line.

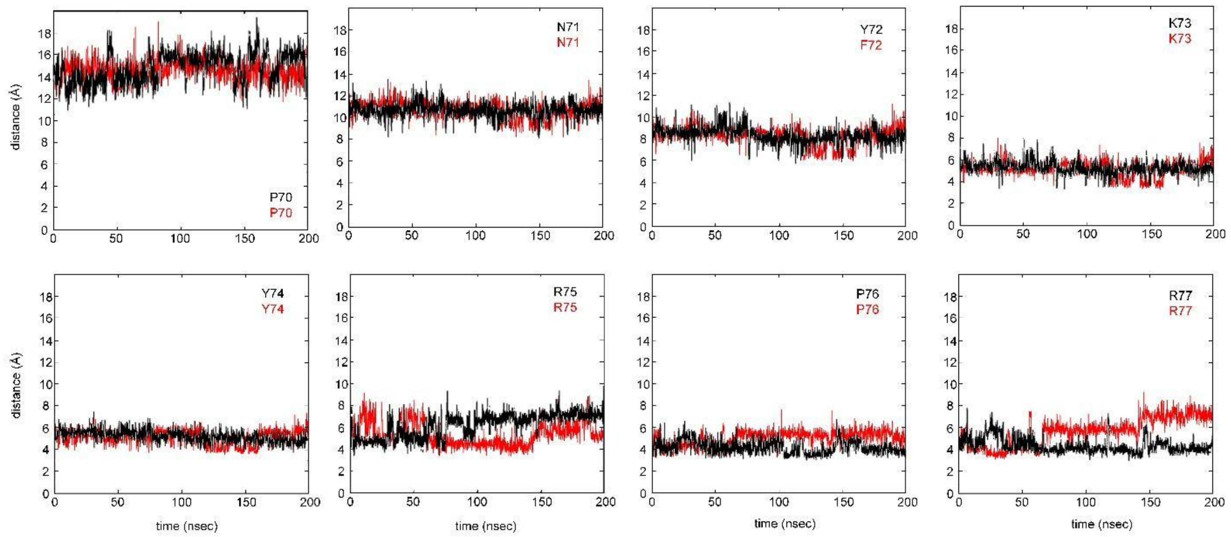

Figure S20 │ Sox18-DNA complex C-terminal tail trajectories over the course of 200ns MD simulation. Each panel represents a plot of the trajectories for the α-carbon of a tail residue to the nearest phosphate group in the protein-DNA complex. In each panel the residue is labeled (WT complex in black and variant complex in red) using consensus HMG numbering.

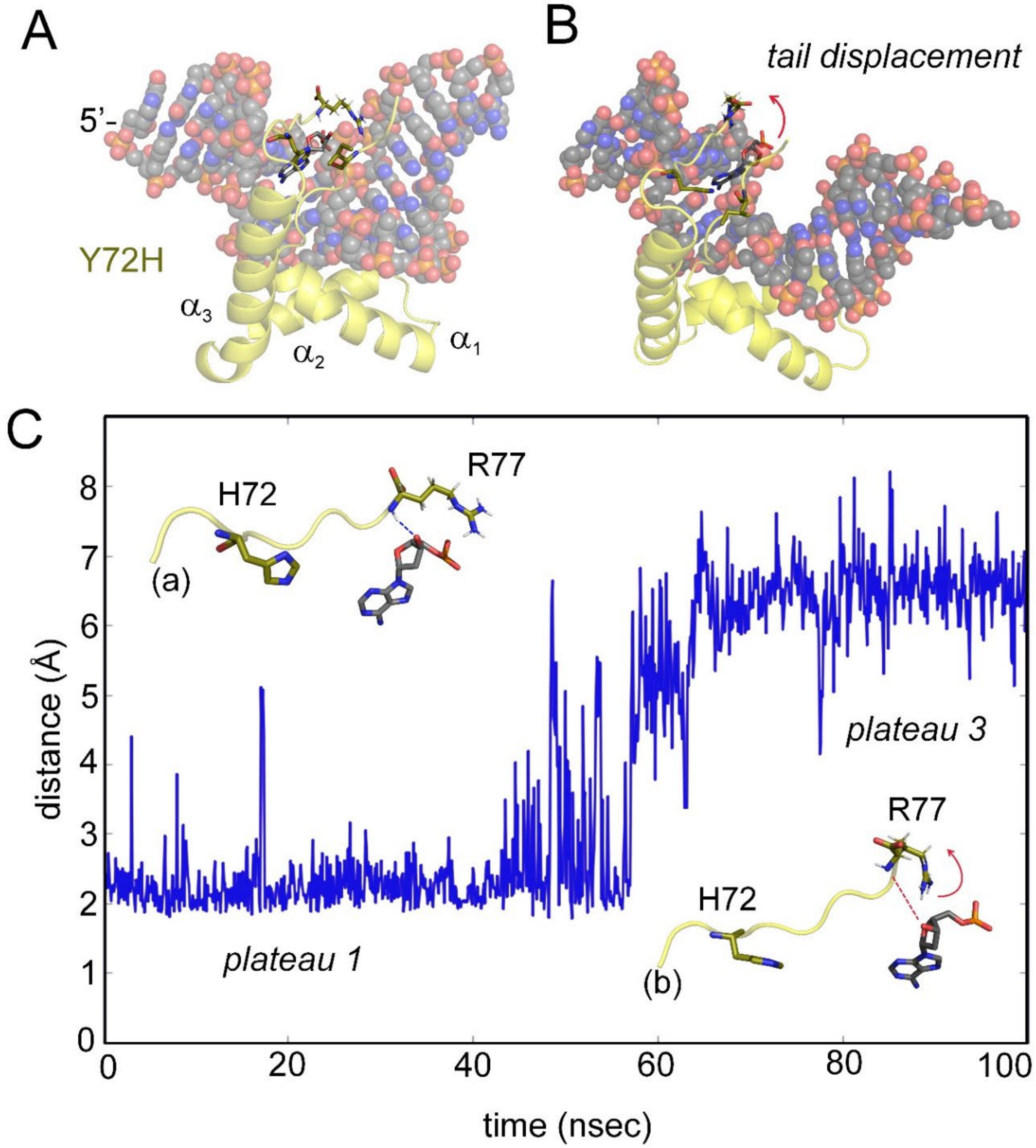

Figure S21 │ Tail displacement of the Y72H variant Sox18-DNA complex. (**A**) Model of the Y72H SOX18 complex at an early time point during the MD time course. The protein domain is in yellow with helices 1-3 labeled, DNA is shown as spheres. Residue side chains of Ile5, His72, and R77 are shown as sticks. (**B**) Representation of a late time end frame point in the MD time course exhibiting displacement of the C-terminal tail, specifically the position of Arg77, indicated by the curved red arrow. (**C**) Trajectory of the R77 amide to the cytosine phosphate on the bottom strand (plateau 1) and displacement away from this site (plateau 3). Insets highlight structural features and tail orientation at each of the plateaus.

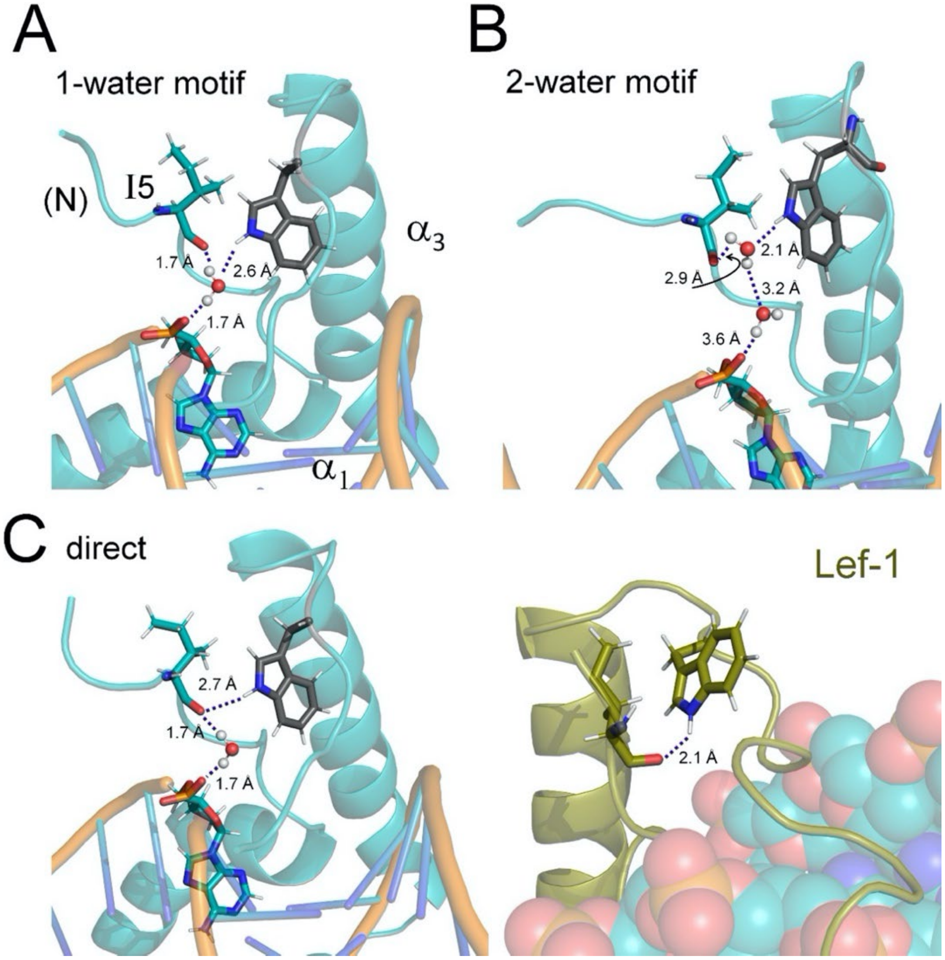

Figure S22 | Molecular dynamic simulation of the Y72W and water occupancy at "magic" site with possible mode of interaction. (A) Structural model of a "1-water motif" of the Y72W-DNA complex. Water molecule is shown as a red and white sphere model, hydrogen bond partners are shown as sticks in the protein and DNA, hydrogen bond lengths are highlighted by broken lines and distances given. (B) Second "2-water motif" for later time point during MD analysis. Residue side chains and DNA contacts are shown as sticks and water molecules as sphere models. Hydrogen bonding pattern and distances are shown. (C) Observation of a direct hydrogen bond between the indole HN of Trp and the carbonyl oxygen atom of Ile 5 shown in broken line. The DNA associated water remains present. (D) NMR structure of the mouse Lef1-DNA complex indicating a hydrogen bond between the indole ring of naturally-occuring Trp residue and the DNA. Lef-1 image was designed using coordinates obtained from PDB entry 2LEF (102) using Pymol molecular graphics.

Table S1. Structural analysis of SRY-related SOX HMG-box structures

PDB	HMG box	Resolution (Å)	B_{ave} Prot (chain)	B_{ave} DNA (chain)	Distance (Å)	# H_20 molecules
4Y60	Sox18	1.75	35.2 (C)	44.4 (A/B)	4.79	194
6T78	Sox11	2.50	82.7 (A)	113.2 (F/G)	5.47	6
3U2B	Sox4	2.40	68.7 (C)	67.3 (A/B)	7.06	8
4S2Q	Sox9	2.70	79.6 (D)	100.0 (A/B)	5.32	0
[a]3F27	Sox17	2.75	24.7 (D)	26.7 (A/B)	5.15	0
1GTO	Sox2	2.60	67.3 (D)	54.2 (A/B)	5.08	60
1J46	Sry	NMR			6.74	

[a] Reported B-factors for this structure are unreasonably low.

Table S2. Molecular dynamic analysis of variant HMG box domains

starting structure	MD length (ns)	retention of long-lived water	retention of Y74 orientation[d]	retention of R77 H bond[e]
PDB:1J46 SRY HMG[a]	0-200	after 160ns	yes	yes
1J46: SRY Model	200-400	yes	yes	yes
PDB:4Y60 Sox18 HMG[b]	200	yes	yes	yes
Sox18 Y72F	200	yes; I5-DNA	yes	no
Sox18 Y72W	100	no	yes	yes
Sox18 Y72W (80ns WT model)[c]	100	yes, sometimes direct H-bond observed for indole NHW72and Ile5 CO	yes	yes
Sox18 Y72H (80ns WT model)	100	no-His; Partly I5-DNA	yes	no
Sox18 Y72A (80ns WT model)	100	yes I5-DNA	yes	yes
Sox18 Y72Q (80ns WT model)	100	no-Gln; yes I5-DNA	yes	yes

[a] coordinates from PDB entry 1J46

[b] coordinates from PDB entry 4Y60

[c] models for these variants were designed using the WT Sox18 model after 80ns in MD simulation

[d] retention of the side chain orientation of Y74 was determined by measuring the distance from the para-hydroxyl oxygen to a nucleobase which forms a hydrogen bond

[e] retained hydrogen bond described here is between the amide group of R77 and a phosphate oxygen on the bottom DNA strand throughout the course of the entire MD simulation

Table S3. PGE DNA bending angles and peak shifts from CD

sample	λ_{max} (nm)	$\Delta\lambda$ (nm)	DNA bend angle (θ)[a]	DNA bend angle (θ)	DNA bend angle (θ)	DNA bend angle (θ)
free DNA	281.7	--	--	--	--	--
WT	270.2	11.5	83.0	81.7	81.6	81.5
Y72F	270.8	10.9	78.8	77.9	82.6	80.8
Y72W	271.3	10.4	78.5	81.4	80.2	82.5
Y72A	272.2	9.5	67.3	65.1	70.5	73.3
free DNA[b]	278.7	--	--	--	--	--
free DNA + TFE	270.4	8.3	--	--	--	--

[a] DNA bend angles from four independent gel electrophoresis experiments for each sample.

[b] Control circular dichroism experiment with free DNA in 10mM sodium phosphate, 0.6 mM EDTA, pH 7.0 and DNA in same buffer with 80% trifluoroenthanol (TFE) to induce A-form DNA CD spectra (103).

Table S4. time resolved FRET (trFRET) values

sample	Distance (Å)	Width[a] (Å)	Energy transfer (%)	Global[b] (χ^2)
DNA alone	60.7<br>(60.4 – 61.1)	11.3<br>(8.1 – 13.8)	45.0	1.05
WT	51.0<br>(50.2 – 51.8	16.7<br>(15.0 – 18.5)	71.0	1.10
Y72F	51.8<br>(50.6 – 52.8)	18.9<br>(15.3 – 20.9)	67.5	1.12

Values are the average of two independent measurements

[a] Width is full width at half-maximum (FWHM)

[b] Global (χ^2) co-optimized global fitting of all FRET data to obtain best single distance distribution.

Table S5. ^{1}H-NMR Imino Chemical Shifts at 25 °C (15mer)

base pair	base	free DNA	SRY-p	Δδ[b]	Y72F SRP-p	Δδ[c]	ΔΔδ[d]
1	G1	--	--	--	--	--	--
2	G2	13.04	13.08	0.04	13.04	0.02	0.02
3	G3	12.99	12.97	-0.02	13.04	0.05	-0.07
4	G4	12.74	12.84	0.1	12.89	0.15	-0.05
5	T5	13.48	13.48	0	13.53	0.05	-0.05
6	G6	12.33	12.84	0.51	12.87	0.54	-0.03
7	T7′	13.37	13.36	-0.01	13.36	-0.02	0.01
8	T8	13.61	12.64	1.03	12.65	-0.96	-0.01
9	T9	13.48	13.66	0.18	13.73	0.25	-0.07
10	G10	12.33	12.12	-0.21	12.21	-0.12	-0.09
11	T11	13.79	14.35	0.56	14.31	0.52	0.04
12	T12	13.79	13.82	0.03	13.84	0.05	-0.02
13	G13′	12.65	12.62	-0.03	12.65	0	-0.03
14	T14′	13.86	13.88	0.02	13.88	0.02	0
15	G15	--	--	--		--	--

[a] The DNA site is 5′-TCGGTGATTGTTCAG-3′ and complement. [b]Δδ and [b]Δδ is defined as the difference between chemical shifts in the wild-type ad variant respectively. Primes in column indicate lower strand SRY-p complex and the free DNA. [d]ΔΔδ is defined as the difference in chemical shifts between wild-type and mutant complexes.

Table S6. ^{1}H chemical shifts of tryptophan indole ring in free and bound form.

Residue	TRP W15		TRP W43		TRP W52	
Chemical shift (ppm)	^{1}H	^{15}N	^{1}H	^{15}N	^{1}H	^{15}N
WT (*free*)	10.23	130.70	10.00	129.13	9.93	127.65
Y72F(free)	10.22	130.66	9.99	129.14	9.94	127.68
WT (bound)	10.31	130.44	10.89	131.18	10.05	129.14
Y72F(bound)	10.26	130.42	10.90	131.17	10.03	129.14

Table S7. Equilibrium and kinetic DNA binding studies of WT and variant HMG domains

HMG box	K_d[a] (nM) 15 ºC	K_d (nM) 37 ºC	k_{off} (s^{-1}) 10 ºC	k_{off} (s^{-1}) 15 ºC	k_{off} (s^{-1}) 25 ºC	k_{off} (s^{-1}) 37 ºC	fold Δ k_{off} 37 ºC
WT	10 ±3	18 ±2	0.056 ±0.0005	0.058 ±0.0006	0.071 ±0.0005	0.13 ±0.0001	--
Y72A	32 ±10	25 ±5	0.94 ±0.04	1.02 ±0.04	1.3 ±0.004	ND	>20x faster
Y72C	21 ±5	23 ±4	1.47 ±0.07	1.7 ±0.05	ND	ND	>20x faster
Y72F	12 ±3	34 ±1	0.26 ±0.0002	0.26 ±0.0004	0.31 ±0.003	0.55 ±0.005	4.2x faster
Y72H	19 ±4	14 ±1	0.80 ±0.01	0.88 ±0.01	1.1 ±0.02	ND	>20x faster
Y72W	12 ±2	40 ±7	0.083 ±0.0004	0.083 ±0.0004	0.099 ±0.04	0.23 ±0.001	1.7x faster

[a] We have reported the values for clinical mutations Y72C, Y72F, Y72H and WT at 15 ºC in our companion paper (23).

Table S8. Calculated lifetimes and inferred *on*-rates

Sample	lifetime (s) 10 °C	15 °C	25 °C	37 °C	k_{on} ($M^{-1}s^{-1}$) (15 °C)	k_{on} ($M^{-1}s^{-1}$) (37 °C)
WT	17.6 ±0.2	17.2 ±0.15	13.9 ±0.44	7.6 ±0.17	5.8 ±0.15	7.3 ±1.3
Y72A	0.98 ±0.13	0.95 ±0.11	0.70 ±0.09	0.23 ±0.03	33.0 ±3.2	--
Y72C	0.67 ±0.03	0.56 ±0.03	0.37 ±0.05	0.19 ±0.03	82.5 ±2.9	--
Y72F	3.8 ±0.05	3.9 ±0.1	3.3 ±0.12	1.8 ±0.1	20.8 ±0.7	21.8 ±0.9
Y72H	1.3 ±0.01	1.1 ±0.01	0.86 ±0.07	0.47 ±0.02	45.6 ±0.5	
Y72W	12.0 ±0.06	12.0 ±0.06	10.1 ±0.04	4.3 ±0.2	6.9 ±1.2	5.8 ±1.0

Table S9. Bridging water interactions in homologous HMG structures

HMG Box[a]	Water	Protein atom (C)	Distance (Å)	DNA atom	Distance (Å)
		Amino acid side chain – H_2O- DNA backbone			
Sox18	106-C	HE2 H29	2.60	OP1 DT 13-B	2.76
	137-C	NZ K35	3.03	OP1 DA 7-A	3.04
	173-C	OH Y70 (72)	2.57	OP1 DA 8-B	2.66
		O I3	3.08		
		Protein main chain –H_2O-DNA backbone			
	119-A	N Y72	2.87	OP1 DT 13-A	2.78
	167-C	O M11	2.78	O4’ DA 9-B	3.02
	171-C	O R5	2.76	OP1 DA 9-B	3.13
	195-C	NH1 R60 (62)	2.99	OP1 DC 12-A	3.16
		Protein main chain/side chain-H_2O-Nucleobase			
	107-A	ND2 N8	3.38	O4’ DT 11-A	2.98
				N2 DG 10-A	2.96
	108-A	N A31	2.91	O2 DC 6-A	2.88
	176-C	NE R18	2.74	N3 DA 9-B	2.87
	184-C	N N30	2.85	N2 DG 11-B	3.16
		Bridging contacts in other Sox structures			
Sox 11	201-A	OH Y116-A (72)	2.49	OP1 DA 13-G	3.26
		O I49	2.59		
Sox 2	2015-B	OH Y70-D (72)	2.90	OP1 DA 44–B	2.74
		O V3	2.83		

[a] The PDB entries for the structures in this analysis are: 4Y60 (Sox18), 6T78 (Sox11) and 1GTO (Sox2)

Table S10. Inherited SRY mutations

mutation	box position	clinical report.	molecular studies
R30I	--	(33)	
V60L	5	(34)	(29)
M64V	9	(35)	
R76S	21	(36)	
I90M	35	(37)	(26)
F109S	54	(38)	(39)
Y127F	72	(40)	(23)
L163X	--	(41)[a]	

[a]A truncated SRY variant was reported as "familial" due to its presence is related XY sex-reversed sisters; however the father was unable for genetic testing. See also footnote 1.

Table S11. SRY tail-related mutations[a]

mutation	box positio n	location	genetics	ref.
Q57R	2	β-strand	*de novo*	(42)
D58E	3	β-strand	*de novo*	(43)
R59G	4	β-strand	*de novo*	(44)
V60A	5	minor wing/tail junction	unknown	(45)
V60L	5	minor wing/tail junction	inherited	(34)
R62G	7	β-strand	*de novo*	(46)
R62P	7	β-strand	*de novo*	(47)
M64I	9	minor wing/tail junction	*de novo*	(34)
M64R	9	minor wing/tail junction	*de novo*	(48)
M64V	9	minor wing/tail junction	Inherited	(35)
P125L	70	minor wing/tail junction	*de novo*	(49)
Y127C	72	minor wing/tail junction	*de novo*	(50)
Y127F	72	minor wing/tail junction	inherited	(40)
Y127H	72	minor wing/tail junction	*de novo*	(51)
K128R	73	tail	*de novo*	(52)
Y129N	74	tail	*de novo*	(53)
R130P	75	tail	*de novo*	(54)
P131H	76	tail	*de novo*	(55)
P131R	76	tail	*de novo*	(56)
R132G	77	tail	*de novo*	(57)

[a]Table generated from Human Genome Mutation Database (HGMD) https://digitalinsights.qiagen.com/products-overview/clinical-insights-portfolio/human-gene-mutation-database/ See also footnote 1.

Table S12. SOX family tail-related mutations[a]

Gene	mutation	box position	location	ref.
SOX2	P44R	8	minor wing/tail junction	(58)
	Y110C	74	tail	(59)
	P112L	76	tail	(60)
SOX3	P142T	8	minor wing/tail junction	(61)
SOX4	I59S	5	minor wing/tail junction	(62)
SOX5	R558C	7	β-strand	(63)
	R558H	7	β-strand	(64)
	M560V	9	minor wing/tail junction	(65)
	Y623C	72	minor wing/tail junction	(65)
	T632N	82	tail	(66)
SOX9	K106E	6	β-strand	(67)
	P108L	8	minor wing/tail junction	(68)
	M109L	9	minor wing/tail junction	(69)
	P170L	70	minor wing/tail junction	(70)
	P170R	70	minor wing/tail junction	(68)
	P170S	70	minor wing/tail junction	(71)
	K173E	73	tail	(72)
	P176L	76	tail	(73)
	R177W	77	tail	(74)
	R178L	78	tail	(75)
SOX10	R106W	7	β-strand	(76)
	P107S	8	minor wing/tail junction	(77)
	M108T	9	minor wing/tail junction	(78)
	P169L	70	minor wing/tail junction	(79)
	Y171H	72	minor wing/tail junction	(80):
	Q174P	75	tail	(81)
	P175A	76	tail	(76)
	P175L	76	tail	(76)
	P175R	76	tail	(76)
	P175S	76	tail	(82)
	R177Q	78	tail	(83)
	A184T	85	tail	(84)
SOX11	G47S	3	β-strand	(85)
	I49S	5	minor wing/tail junction	(86)

	K50N	6	β-strand	(87)
	P52S	8	minor wing/tail junction	(88)
	Y116C	72	minor wing/tail junction	(89)
	P120H	76	tail	(87)
	P120L	76	tail	(90)
SOX17	R70Q	7	β-strand	(91)
	P133A	70	minor wing/tail junction	(91)
	P133L	70	minor wing/tail junction	(92)
	P133S	70	minor wing/tail junction	(93)
	R138P	75	tail	(94)
	P139S	76	tail	(95)
	R140P	77	tail	(93)
	R140W	77	tail	(96)

[a]Table generated from Human Genome Mutation Database (HGMD) https://digitalinsights.qiagen.com/products-overview/clinical-insights-portfolio/human-gene-mutation-database/. See also footnote 1.

Footnotes

[1]Homologous clinical mutations in SRY and SOX genes give rise to different clinical syndromes depending on the respective biological function of the specific family members. Whereas mutations in SRY, for example, yield DSD phenotypes, mutations in the HMG box of SOX10 are associated with Waardenburg syndrome type II and type IV (also designated Waardenburg- Shah syndrome), characterized by impaired hearing with changes in skin, hair, and eye coloring (97). Mutations in the HMG box of SOX9 cause campomelic dysplasia, characterized by abnormalities of cartilage and bone in variable association with XY DSD (98). Developmental abnormalities of bone are also associated with mutations in SOX4 and SOX11 (99). The diverse SOX-associated genetic syndromes are collectively designated "SOXopathies" (100).

SUPPLEMENTAL REFERENCES

1. Murphy EC, Zhurkin VB, Louis JM, Cornilescu G, Clore GM. Structural Basis for Sry-Dependent 46-X,Y Sex Reversal: Modulation of DNA Bending by a Naturally Occuring Point Mutation. *J Mol Biol* (2001) 312(3):481-99.
2. Tuechsen E, Hayes J, Ramaprasad S, Copie V, Woodward C. Solvent Exchange of Buried Water and Hydrogen Exchange of Peptide Nh Groups Hydrogen Bonded to Buried Waters in Bovine Pancreatic Trypsin Inhibitor. *Biochemistry* (1987) 26(16):5163-72.
3. Venu K, Denisov VP, Halle B. Water 1h Magnetic Relaxation Dispersion in Protein Solutions. A Quantitative Assessment of Internal Hydration, Proton Exchange, and Cross-Relaxation. *J Am Chem Soc* (1997) 119(13):3122-34.
4. Otting G, Liepinsh E, Wüthrich K. Protein Hydration in Aqueous Solution. *Science* (1991) 254(5034):974-80.
5. Otting G. Nmr Studies of Water Bound to Biological Molecules. *Prog Nucl Magn Reson Spectrosc* (1997) 31(2-3):259-85.
6. Brunne R, Liepinsh E, Otting G, Wüthrich K, Van Gunsteren W. Hydration of Proteins: A Comparison of Experimental Residence Times of Water Molecules Solvating the Bovine Pancreatic Trypsin Inhibitor with Theoretical Model Calculations. *J Mol Biol* (1993) 231(4):1040-8.
7. Billeter M, Qian YQ, Otting G, Müller M, Gehring W, Wüthrich K. Determination of the Nuclear Magnetic Resonance Solution Structure of an Antennapedia Homeodomain-DNA Complex. *J Mol Biol* (1993) 234(4):1084-97.
8. Clore GM, Bax A, Wingfield PT, Gronenborn AM. Identification and Localization of Bound Internal Water in the Solution Structure of Interleukin 1. Beta. By Heteronuclear Three-Dimensional Proton Rotating-Fram Overhauser Nitrogen-15-Proton Multiple Quantum Coherence Nmr Spectroscopy. *Biochemistry* (1990) 29(24):5671-6.
9. Clore GM, Bax A, Omichinski JG, Gronenborn AM. Localization of Bound Water in the Solution Structure of a Complex of the Erythroid Transcription Factor Gata-1 with DNA. *Structure* (1994) 2(2):89-94. Epub 1994/02/15.
10. Tsui V, Radhakrishnan I, Wright PE, Case DA. Nmr and Molecular Dynamics Studies of the Hydration of a Zinc Finger-DNA Complex. *J Mol Biol* (2000) 302(5):1101-17.
11. Berndt KD, Beunink J, Schroeder W, Wuethrich K. Designed Replacement of an Internal Hydration Water Molecule in Bpti: Structural and Functional Implications of a Gly-to-Ser Mutation. *Biochemistry* (1993) 32(17):4564-70.
12. Billeter M, Guntert P, Luginbuhl P, Wuthrich K. Hydration and DNA Recognition by Homeodomains. *Cell* (1996) 85(7):1057-65. Epub 1996/06/28.
13. Kozono D, Yasui M, King LS, Agre P. Aquaporin Water Channels: Atomic Structure Molecular Dynamics Meet Clinical Medicine. *J Clin Invest* (2002) 109(11):1395-9.
14. Bourg IC, Steefel CI. Molecular Dynamics Simulations of Water Structure and Diffusion in Silica Nanopores. *J Phys Chem C* (2012) 116(21):11556-64.
15. Raghavan K, Foster K, Motakabbir K, Berkowitz M. Structure and Dynamics of Water at the Pt (111) Interface: Molecular Dynamics Study. *The Journal of chemical physics* (1991) 94(3):2110-7.
16. Rodin VV. Nmr Techniques in Studying Water in Biotechnological Systems. *Biophys Rev* (2020) 12(3):683-701. Epub 20200615. doi: 10.1007/s12551-020-00694-5.
17. V Rodin V, A Nikerov V. Nmr-Relaxation and Pfg Nmr Studies of Water Dynamics in Oriented Collagen Fibres with Different Degree of Cross-Linking. *Curr Tissue Eng* (2014) 3(1):47-61.
18. Tang Y, Nilsson L. Interaction of Human Sry Protein with DNA: A Molecular Dynamics Study. *Proteins* (1998) 31:417-33.
19. Clarkson MJ, Harley VR. Sex with Two Sox On: Sry and Sox9 in Testis Development. *Trends Endocrinol Metab* (2002) 13:106-11.

20. Bhandari RK, Haque MM, Skinner MK. Global Genome Analysis of the Downstream Binding Targets of Testis Determining Factor Sry and Sox9. *PLoS One* (2012) 7(9):e43380-e.
21. Josso N, Picard JY, Rey R, di Clemente N. Testicular Anti-Mullerian Hormone: History, Genetics, Regulation and Clinical Applications. *Pediatr Endocrinol Rev* (2006) 3(4):347-58. Epub 2006/07/04.
22. Read CM, Cary PD, Preston NS, Lnenicek-Allen M, Crane-Robinson C. The DNA Sequence Specificity of Hmg Boxes Lies in the Minor Wing of the Structure. *EMBO J* (1994) 13:5639-46. doi: PMC395529.
23. Chen Y-S, Racca JD, Weiss MA. Tenuous Transcriptional Threshold of Human Sex Determination. I. Sry and Swyer Syndrome at the Edge of Ambiguity. *Front Endocrinol* (2022) 13:945030. doi: 10.3389/fendo.2022.945030
24. Hersmus R, de Leeuw BH, Stoop H, Bernard P, van Doorn HC, Bruggenwirth HT, et al. A Novel Sry Missense Mutation Affecting Nuclear Import in a 46,Xy Female Patient with Bilateral Gonadoblastoma. *Eur J Hum Genet* (2009) 17(12):1642-9. Epub 2009/06/11. doi: 10.1038/ejhg.2009.96.
25. Li B, Zhang W, Chan G, Jancso-Radek A, Liu S, Weiss MA. Human Sex Reversal Due to Impaired Nuclear Localization of Sry. A Clinical Correlation. *J Biol Chem* (2001) 276:46480-4.
26. Chen YS, Racca JD, Phillips NB, Weiss MA. Inherited Human Sex Reversal Due to Impaired Nucleocytoplasmic Trafficking of Sry Defines a Male Transcriptional Threshold. *Proc Natl Acad Sci USA* (2013) 110(38):E3567-76. Epub 2013 Sep 3. doi: 10.1073/pnas.1300828110.
27. Palasingam P, Jaunch R, Ng CK, Kolatkar PR. The Structure of Sox17 Bound to DNA Reveals a Conserved Bending Topology but Selective Protein Interaction Platforms. *J Mol Biol* (2009) 388:619-30.
28. Klaus M, Prokoph N, Girbig M, Wang X, Huang YH, Srivastava Y, et al. Structure and Decoy-Mediated Inhibition of the Sox18/Prox1-DNA Interaction. *Nucleic Acids Res* (2016) 44(8):3922-35. Epub 2016/03/05. doi: 10.1093/nar/gkw130.
29. Phillips NB, Racca J, Chen YS, Singh R, Jancso-Radek A, Radek JT, et al. Mammilian Testis-Determining Factor Sry and the Enigma of Inherited Human Sex Reversal. *J Biol Chem* (2011) 286(42):36787-807. Epub 2011/08/19. doi: 10.1074/jbc.M111.260091.
30. Phillips NB, Jancso-Radek A, Ittah V, Singh R, Chan G, Haas E, et al. Sry and Human Sex Determination: The Basic Tail of the Hmg Box Functions as a Kinetic Clamp to Augment DNA Bending. *J Mol Biol* (2006) 358:172-92.
31. Reményi A, Lins K, Nissen LJ, Reinbold R, Schöler HR, Wilmanns M. Crystal Structure of a Pou/Hmg/DNA Ternary Complex Suggests Differential Assembly of Oct4 and Sox2 on Two Enhancers. *Genes Dev* (2003) 17(16):2048-59.
32. Dodonova SO, Zhu F, Dienemann C, Taipale J, Cramer P. Nucleosome-Bound Sox2 and Sox11 Structures Elucidate Pioneer Factor Function. *Nature* (2020) 580(7805):669-72.
33. Assumpção J, Maciel-Guerra A, Guerra-Junior G, Scolfaro M, Mello M. Two Novel Sry Mutations: The Hmgbox N65h Associated with 46, Xy Pure Gonadal Dysgenesis and the Familial Non-Hmgbox R30i Associated with Different Phenotypes. *J Mol Med* (2002) 80:782-90.
34. Berta P, Hawkins JR, Sinclair AH, Taylor A, Griffiths BL, Goodfellow PN, et al. Genetic Evidence Equating Sry and the Testis-Determining Factor. *Nature* (1990) 348:448-50.
35. Vaiani E, Malosetti C, Marino R, Ramirez P, Garrido NP, Berensztain E, et al. Familial 46, Xy Complete Female External Sex Development and Primary Amenorrhea Along with Hidden Gonad Tumors, Secondary to a Novel P. Met64val Sry Gene Mutation. *ESPE Abstracts* (2014) 82.
36. IMAI A, TAKAGI A, TAMAYA T. A Novel Sex-Determining Region on Y (Sry) Missense Mutation Identified in a 46, Xy Female and Also in the Father. *Endocr J* (1999) 46(5):735-9.
37. Hawkins JR, Taylor A, Goodfellow PN, Migeon CJ, Smith KD, Berkovitz GD. Evidence for Increased Prevalence of *Sry* Mutations in Xy Females with Complete Rather Than Partial Gonadal Dysgenesis. *Am J Hum Genet* (1992) S1:979-84. doi: PMC1682856.

38. Jäger RJ, Harley VR, Pfeiffer RA, Goodfellow PN, Scherer G. A Familial Mutation in the Testis-Determining Gene Sry Shared by Both Sexes. *Hum Genet* (1992) 90:350-5.
39. Racca JD, Chen Y-S, Yang Y, Phillips NB, Weiss MA. Human Sex Determination at the Edge of Ambiguity Inherited Xy Sex Reversal Due to Enhanced Ubiquitination and Proteasomal Degradation of a Master Transcription Factor. *J Biol Chem* (2016) 291(42):22173-95.
40. Jordan BK, Jain M, Natarajan S, Frasier SD, Vilain E. Familial Mutation in the Testis-Determining Gene Sry Shared by an Xy Female and Her Normal Father. *J Clin Endocrinol Metab* (2002) 87:3428-32.
41. Tajima T, Nakae J, Shinohara N, Fujieda K. A Novel Mutation Localized in the 3' Non-Hmg Box Region of the Sry Gene in 46,Xy Gonadal Dysgenesis. *Hum Mol Genet* (1994) 3:1187-9.
42. Shahid M, Dhillion VS, Jain N, Hedau S, Diwakar S, Sachdeva P, et al. Two New Novel Point Mutations Localized Upstream and Downstream of the Hmg Box Region of the Sry Gene in Three Indian 46, Xy Females with Sex Reversal and Gonadal Tumour Formation. *Mol Hum Reprod* (2004) 10(7):521-6.
43. Eggers S, Sadedin S, Van Den Bergen JA, Robevska G, Ohnesorg T, Hewitt J, et al. Disorders of Sex Development: Insights from Targeted Gene Sequencing of a Large International Patient Cohort. *Genome Biol* (2016) 17(1):1-21.
44. Fernandez R, Marchal JA, Sanchez A, Pasaro E. A Point Mutation, R59g, within the Hmg-Sry Box in a Female 45, X/46, X, Psu Dic (Y)(Pter→ Q11:: Q11→ Pter). *Hum Genet* (2002) 111(3):242-6.
45. Hiort O, Gramss B, Klauber GT. True Hermaphroditism with 46, Xy Karyotype and a Point Mutation in the Sry Gene. *J Pediatr* (1995) 126(6):1022.
46. Affara NA, Chalmers IJ, Ferguson-Smith MA. Analysis of the Sry Gene in 22 Sex-Reversed Xy Females Identifies Four New Point Mutations in the Conserved DNA Binding Domain. *Hum Mol Genet* (1993) 2(6):785-9.
47. Buonocore F, Clifford-Mobley O, King TF, Striglioni N, Man E, Suntharalingham JP, et al. Next-Generation Sequencing Reveals Novel Genetic Variants (Sry, Dmrt1, Nr5a1, Dhh, Dhx37) in Adults with 46, Xy Dsd. *J Clin Endocrinol Metab* (2019) 3(12):2341-60.
48. Scherer G, Held M, Erdel M, Meschede D, Horst J, Lesniewicz R, et al. Three Novel Sry Mutations in Xy Gonadal Dysgenesis and the Enigma of Xy Gonadal Dysgenesis Cases without Sry Mutations. *Cytogenet Cell Genet* (1998) 80:188-92.
49. Schmitt-Ney M, Thiele H, Kaltwasser P, Bardoni B, Cisternino M, Scherer G. Two Novel Sry Missense Mutations Reducing DNA Binding Identified in Xy Females and Their Mosaic Fathers. *Am J Hum Genet* (1995) 56:862-9. doi: PMC1801192.
50. Poulat F, Soullier S, Goze C, Heitz F, Calas B, Berta P. Description and Functional Implications of a Novel Mutation in the Sex-Determining Gene Sry. *Hum Mutat* (1994) 3:200-4.
51. Tajouri A, Ben Gaied D, Hizem S, Boujelben S, Maazoul F, M'Rad R, et al. Functional Analysis of Mutations at Codon 127 of the Sry Gene Associated with 46,Xy Complete Gonadal Dysgenesis. *Sex Dev* (2017) 11(4):203-9. Epub 2017/08/09. doi: 10.1159/000478718.
52. Hersmus R, van der Zwan YG, Stoop H, Bernard P, Sreenivasan R, Oosterhuis JW, et al. A 46,Xy Female Dsd Patient with Bilateral Gonadoblastoma, a Novel Sry Missense Mutation Combined with a Wt1 Kts Splice-Site Mutation. *PLoS One* (2012) 7(7):e40858. Epub 2012/07/21. doi: 10.1371/journal.pone.0040858.
53. Paris F, Philibert P, Lumbroso S, Baldet P, Charvet JP, Galifer RB, et al. Primary Amenorrhea in a 46,Xy Adolescent Girl with Partial Gonadal Dysgenesis: Identification of a New *Sry* Gene Mutation. *Fertil Steril* (2007) 88(5):1437.e21-.e5.
54. De Sousa S, Kassahn KS, McIntyre LC, Chong C-E, Scott HS, Torpy DJ. Case Report of Whole Genome Sequencing in the Xy Female: Identification of a Novel Sry Mutation and Revision of a Misdiagnosis of Androgen Insensitivity Syndrome. *BMC Endocr Disord* (2016) 16(1):1-7.
55. Yu B-Q, Liu Z-X, Gao Y-J, Wang X, Mao J-F, Nie M, et al. Prevalence of Gene Mutations in a Chinese 46, Xy Disorders of Sex Development Cohort Detected by Targeted Next-Generation Sequencing. *Asian J Androl* (2021) 23(1):69.

56. Lundberg Y, Ritzén M, Harlin J, Wedell A. Novel Missense Mutation (P131r) in the Hmg Box of Sry in Xy Sex Reversal. *Hum Mutat* (1998) Suppl. 1:S328.
57. Shahid M, Dhillion VS, Jain N, Hedau S, Diwakar S, Sachdeva P, et al. Two New Novel Point Mutations Localized Upstream and Downstream of the Hmg Box Region of the *Sry* Gene in Three Indian 46,Xy Females with Sex Reversal and Gonadal Tumour Formation. *Mol Human Reprod* (2004) 10:521-6.
58. Schneider A, Bardakjian T, Reis LM, Tyler RC, Semina EV. Novel Sox2 Mutations and Genotype–Phenotype Correlation in Anophthalmia and Microphthalmia. *Am J Med Genet* (2009) 149(12):2706-15.
59. Takagi M, Narumi S, Asakura Y, Muroya K, Hasegawa Y, Adachi M, et al. A Novel Mutation in Sox2 Causes Hypogonadotropic Hypogonadism with Mild Ocular Malformation. *Horm Res Paediatr* (2014) 81(2):133-8.
60. Dennert N, Engels H, Cremer K, Becker J, Wohlleber E, Albrecht B, et al. De Novo Microdeletions and Point Mutations Affecting Sox2 in Three Individuals with Intellectual Disability but without Major Eye Malformations. *Am J Med Genet A* (2017) 173(2):435-43. Epub 20161114. doi: 10.1002/ajmg.a.38034.
61. Li J, Zhong Y, Guo T, Yu Y, Li J. Case Report: A Novel Point Mutation of Sox3 in a Subject with Growth Hormone Deficiency, Hypogonadotrophic Hypogonadism, and Borderline Intellectual Disability. *Front Endocrinol (Lausanne)* (2022) 13:810375. Epub 20220228. doi: 10.3389/fendo.2022.810375.
62. Zawerton A, Yao B, Yeager JP, Pippucci T, Haseeb A, Smith JD, et al. De Novo Sox4 Variants Cause a Neurodevelopmental Disease Associated with Mild Dysmorphism. *Am J Hum Genet* (2019) 104(4):777. doi: 10.1016/j.ajhg.2019.01.014.
63. Innella G, Greco D, Carli D, Magini P, Giorgio E, Galesi O, et al. Clinical Spectrum and Follow-up in Six Individuals with Lamb-Shaffer Syndrome (Sox5). *Am J Med Genet A* (2021) 185(2):608-13. Epub 20201209. doi: 10.1002/ajmg.a.62001.
64. Martinez-Granero F, Blanco-Kelly F, Sanchez-Jimeno C, Avila-Fernandez A, Arteche A, Bustamante-Aragones A, et al. Comparison of the Diagnostic Yield of Acgh and Genome-Wide Sequencing across Different Neurodevelopmental Disorders. *NPJ Genom Med* (2021) 6(1):25. Epub 20210325. doi: 10.1038/s41525-021-00188-7.
65. Zawerton A, Mignot C, Sigafoos A, Blackburn PR, Haseeb A, McWalter K, et al. Widening of the Genetic and Clinical Spectrum of Lamb-Shaffer Syndrome, a Neurodevelopmental Disorder Due to Sox5 Haploinsufficiency. *Genet Med* (2020) 22(3):524-37. Epub 20191003. doi: 10.1038/s41436-019-0657-0.
66. Cherot E, Keren B, Dubourg C, Carre W, Fradin M, Lavillaureix A, et al. Using Medical Exome Sequencing to Identify the Causes of Neurodevelopmental Disorders: Experience of 2 Clinical Units and 216 Patients. *Clin Genet* (2018) 93(3):567-76. Epub 20171004. doi: 10.1111/cge.13102.
67. Gentilin B, Forzano F, Bedeschi MF, Rizzuti T, Faravelli F, Izzi C, et al. Phenotype of Five Cases of Prenatally Diagnosed Campomelic Dysplasia Harboring Novel Mutations of the *Sox9* Gene. *Ultrasound Obstet Gynecol* (2010) 36(3):315-23. doi: 10.1002/uog.7761.
68. Meyer J, Sudbeck P, Held M, Wagner T, Schmitz ML, Bricarelli FD, et al. Mutational Analysis of the Sox9 Gene in Campomelic Dysplasia and Autosomal Sex Reversal: Lack of Genotype/Phenotype Correlations. *Hum Mol Genet* (1997) 6(1):91-8. doi: 10.1093/hmg/6.1.91.
69. Baker SW, Murrell JR, Nesbitt AI, Pechter KB, Balciuniene J, Zhao X, et al. Automated Clinical Exome Reanalysis Reveals Novel Diagnoses. *J Mol Diagn* (2019) 21(1):38-48. doi: 10.1016/j.jmoldx.2018.07.008.
70. Wada Y, Nishimura G, Nagai T, Sawai H, Yoshikata M, Miyagawa S, et al. Mutation Analysis of Sox9 and Single Copy Number Variant Analysis of the Upstream Region in Eight Patients with Campomelic Dysplasia and Acampomelic Campomelic Dysplasia. *Am J Med Genet A* (2009) 149A(12):2882-5. doi: 10.1002/ajmg.a.33107.

71. Retterer K, Juusola J, Cho MT, Vitazka P, Millan F, Gibellini F, et al. Clinical Application of Whole-Exome Sequencing across Clinical Indications. *Genet Med* (2016) 18(7):696-704. Epub 20151203. doi: 10.1038/gim.2015.148.
72. Thong MK, Scherer G, Kozlowski K, Haan E, Morris L. Acampomelic Campomelic Dysplasia with Sox9 Mutation. *Am J Med Genet* (2000) 93(5):421-5.
73. Michel-Calemard L, Lesca G, Morel Y, Boggio D, Plauchu H, Attia-Sobol J. Campomelic Acampomelic Dysplasia Presenting with Increased Nuchal Translucency in the First Trimester. *Prenat Diagn* (2004) 24(7):519-23. doi: 10.1002/pd.935.
74. Wei H, Lai A, Tan ES, Koh MJA, Ng I, Ting TW, et al. Genetic Landscape of Congenital Disorders in Patients from Southeast Asia: Results from Sequencing Using a Gene Panel for Mendelian Phenotypes. *Arch Dis Child* (2021) 106(1):38-43. Epub 20200925. doi: 10.1136/archdischild-2020-319177.
75. Cui Y, Zhao H, Liu Z, Liu C, Luan J, Zhou X, et al. A Systematic Review of Genetic Skeletal Disorders Reported in Chinese Biomedical Journals between 1978 and 2012. *Orphanet J Rare Dis* (2012) 7:55. Epub 20120822. doi: 10.1186/1750-1172-7-55.
76. Chaoui A, Watanabe Y, Touraine R, Baral V, Goossens M, Pingault V, et al. Identification and Functional Analysis of Sox10 Missense Mutations in Different Subtypes of Waardenburg Syndrome. *Hum Mutat* (2011) 32(12):1436-49. Epub 20110919. doi: 10.1002/humu.21583.
77. Rojas RA, Kutateladze AA, Plummer L, Stamou M, Keefe DL, Jr., Salnikov KB, et al. Phenotypic Continuum between Waardenburg Syndrome and Idiopathic Hypogonadotropic Hypogonadism in Humans with Sox10 Variants. *Genet Med* (2021) 23(4):629-36. Epub 20210113. doi: 10.1038/s41436-020-01051-3.
78. Pingault V, Bodereau V, Baral V, Marcos S, Watanabe Y, Chaoui A, et al. Loss-of-Function Mutations in Sox10 Cause Kallmann Syndrome with Deafness. *Am J Hum Genet* (2013) 92(5):707-24. doi: 10.1016/j.ajhg.2013.03.024.
79. Pingault V, Faubert E, Baral V, Gherbi S, Loundon N, Couloigner V, et al. Sox10 Mutations Mimic Isolated Hearing Loss. *Clin Genet* (2015) 88(4):352-9. Epub 20141106. doi: 10.1111/cge.12506.
80. Ren S, Chen X, Kong X, Chen Y, Wu Q, Jiao Z, et al. Identification of Six Novel Variants in Waardenburg Syndrome Type Ii by Next-Generation Sequencing. *Mol Genet Genomic Med* (2020) 8(3):e1128. Epub 20200120. doi: 10.1002/mgg3.1128.
81. Barnett CP, Mendoza-Londono R, Blaser S, Gillis J, Dupuis L, Levin AV, et al. Aplasia of Cochlear Nerves and Olfactory Bulbs in Association with Sox10 Mutation. *Am J Med Genet A* (2009) 149A(3):431-6. doi: 10.1002/ajmg.a.32657.
82. Powis Z, Farwell Hagman KD, Speare V, Cain T, Blanco K, Mowlavi LS, et al. Exome Sequencing in Neonates: Diagnostic Rates, Characteristics, and Time to Diagnosis. *Genet Med* (2018) 20(11):1468-71. Epub 20180322. doi: 10.1038/gim.2018.11.
83. Cassatella D, Howard SR, Acierno JS, Xu C, Papadakis GE, Santoni FA, et al. Congenital Hypogonadotropic Hypogonadism and Constitutional Delay of Growth and Puberty Have Distinct Genetic Architectures. *Eur J Endocrinol* (2018) 178(4):377-88. Epub 20180201. doi: 10.1530/EJE-17-0568.
84. Yavarna T, Al-Dewik N, Al-Mureikhi M, Ali R, Al-Mesaifri F, Mahmoud L, et al. High Diagnostic Yield of Clinical Exome Sequencing in Middle Eastern Patients with Mendelian Disorders. *Hum Genet* (2015) 134(9):967-80. Epub 20150616. doi: 10.1007/s00439-015-1575-0.
85. Hanker B, Gillessen-Kaesbach G, Huning I, Ludecke HJ, Wieczorek D. Maternal Transmission of a Mild Coffin-Siris Syndrome Phenotype Caused by a Sox11 Missense Variant. *Eur J Hum Genet* (2022) 30(1):126-32. Epub 20210331. doi: 10.1038/s41431-021-00865-2.
86. Brunet T, Jech R, Brugger M, Kovacs R, Alhaddad B, Leszinski G, et al. De Novo Variants in Neurodevelopmental Disorders-Experiences from a Tertiary Care Center. *Clin Genet* (2021) 100(1):14-28. Epub 20210301. doi: 10.1111/cge.13946.
87. Hempel A, Pagnamenta AT, Blyth M, Mansour S, McConnell V, Kou I, et al. Deletions and De Novo Mutations of Sox11 Are Associated with a Neurodevelopmental Disorder with Features of

Coffin-Siris Syndrome. *J Med Genet* (2016) 53(3):152-62. Epub 20151105. doi: 10.1136/jmedgenet-2015-103393.
88. Sekiguchi F, Tsurusaki Y, Okamoto N, Teik KW, Mizuno S, Suzumura H, et al. Genetic Abnormalities in a Large Cohort of Coffin-Siris Syndrome Patients. *J Hum Genet* (2019) 64(12):1173-86. Epub 20190917. doi: 10.1038/s10038-019-0667-4.
89. Tsurusaki Y, Koshimizu E, Ohashi H, Phadke S, Kou I, Shiina M, et al. De Novo Sox11 Mutations Cause Coffin-Siris Syndrome. *Nat Commun* (2014) 5:4011. Epub 20140602. doi: 10.1038/ncomms5011.
90. Bertoli-Avella AM, Beetz C, Ameziane N, Rocha ME, Guatibonza P, Pereira C, et al. Successful Application of Genome Sequencing in a Diagnostic Setting: 1007 Index Cases from a Clinically Heterogeneous Cohort. *Eur J Hum Genet* (2021) 29(1):141-53. Epub 20200828. doi: 10.1038/s41431-020-00713-9.
91. Hiraide T, Kataoka M, Suzuki H, Aimi Y, Chiba T, Kanekura K, et al. Sox17 Mutations in Japanese Patients with Pulmonary Arterial Hypertension. *Am J Respir Crit Care Med* (2018) 198(9):1231-3. doi: 10.1164/rccm.201804-0766LE.
92. Zhu N, Welch CL, Wang J, Allen PM, Gonzaga-Jauregui C, Ma L, et al. Rare Variants in Sox17 Are Associated with Pulmonary Arterial Hypertension with Congenital Heart Disease. *Genome Med* (2018) 10(1):56. Epub 20180720. doi: 10.1186/s13073-018-0566-x.
93. Graf S, Haimel M, Bleda M, Hadinnapola C, Southgate L, Li W, et al. Identification of Rare Sequence Variation Underlying Heritable Pulmonary Arterial Hypertension. *Nat Commun* (2018) 9(1):1416. Epub 20180412. doi: 10.1038/s41467-018-03672-4.
94. Eichstaedt CA, Sassmannshausen Z, Shaukat M, Cao D, Xanthouli P, Gall H, et al. Gene Panel Diagnostics Reveals New Pathogenic Variants in Pulmonary Arterial Hypertension. *Respir Res* (2022) 23(1):74. Epub 20220327. doi: 10.1186/s12931-022-01987-x.
95. Shieh JT, Penon-Portmann M, Wong KHY, Levy-Sakin M, Verghese M, Slavotinek A, et al. Application of Full-Genome Analysis to Diagnose Rare Monogenic Disorders. *NPJ Genom Med* (2021) 6(1):77. Epub 20210923. doi: 10.1038/s41525-021-00241-5.
96. Zhu Z, Zhu X, Liu CL, Shi H, Shen S, Yang Y, et al. Shared Genetics of Asthma and Mental Health Disorders: A Large-Scale Genome-Wide Cross-Trait Analysis. *Eur Respir J* (2019) 54(6). Epub 20191219. doi: 10.1183/13993003.01507-2019.
97. Thongpradit S, Jinawath N, Javed A, Jensen LT, Chunsuwan I, Rojnueangnit K, et al. Novel Sox10 Mutations in Waardenburg Syndrome: Functional Characterization and Genotype-Phenotype Analysis. *Front genet* (2020):1519.
98. Meyer J, Südbeck P, Held M, Wagner T, Schmitz ML, Dagna Bricarelli F, et al. Mutational Analysis of the Sox9 Gene in Campomelic Dysplasia and Autosomal Sex Reversal: Lack of Genotype/Phenotype Correlations. *Hum Mol Genet* (1997) 6(1):91-8.
99. Lefebvre V. Roles and Regulation of Sox Transcription Factors in Skeletogenesis. *Curr Top Dev Biol* (2019) 133:171-93.
100. Angelozzi M, Lefebvre V. Soxopathies: Growing Family of Developmental Disorders Due to Sox Mutations. *Trends Genet* (2019) 35(9):658-671. Epub 2019 Jul 6. doi: 10.1016/j.tig.2019.06.003.
101. Narayana, Narendra, and Michael A. Weiss. "Crystallographic analysis of a sex-specific enhancer element: sequence-dependent DNA structure, hydration, and dynamics." *Journal of molecular biology* 385.2 (2009): 469-490.
102. Love, John J., et al. "Structural basis for DNA bending by the architectural transcription factor LEF-1." *Nature* 376.6543 (1995): 791-795.
103. Kypr, Jaroslav, et al. "Aqueous trifluorethanol solutions simulate the environment of DNA in the crystalline state." *Nucleic acids research* 27.17 (1999): 3466-3473.
